# Sex-stratified Genome-wide Association Studies Including 270,000 Individuals Show Sexual Dimorphism in Genetic Loci for Anthropometric Traits

**DOI:** 10.1371/journal.pgen.1003500

**Published:** 2013-06-06

**Authors:** Joshua C. Randall, Thomas W. Winkler, Zoltán Kutalik, Sonja I. Berndt, Anne U. Jackson, Keri L. Monda, Tuomas O. Kilpeläinen, Tõnu Esko, Reedik Mägi, Shengxu Li, Tsegaselassie Workalemahu, Mary F. Feitosa, Damien C. Croteau-Chonka, Felix R. Day, Tove Fall, Teresa Ferreira, Stefan Gustafsson, Adam E. Locke, Iain Mathieson, Andre Scherag, Sailaja Vedantam, Andrew R. Wood, Liming Liang, Valgerdur Steinthorsdottir, Gudmar Thorleifsson, Emmanouil T. Dermitzakis, Antigone S. Dimas, Fredrik Karpe, Josine L. Min, George Nicholson, Deborah J. Clegg, Thomas Person, Jon P. Krohn, Sabrina Bauer, Christa Buechler, Kristina Eisinger, DIAGRAM Consortium, Amélie Bonnefond, Philippe Froguel, MAGIC Investigators, Jouke-Jan Hottenga, Inga Prokopenko, Lindsay L. Waite, Tamara B. Harris, Albert Vernon Smith, Alan R. Shuldiner, Wendy L. McArdle, Mark J. Caulfield, Patricia B. Munroe, Henrik Grönberg, Yii-Der Ida Chen, Guo Li, Jacques S. Beckmann, Toby Johnson, Unnur Thorsteinsdottir, Maris Teder-Laving, Kay-Tee Khaw, Nicholas J. Wareham, Jing Hua Zhao, Najaf Amin, Ben A. Oostra, Aldi T. Kraja, Michael A. Province, L. Adrienne Cupples, Nancy L. Heard-Costa, Jaakko Kaprio, Samuli Ripatti, Ida Surakka, Francis S. Collins, Jouko Saramies, Jaakko Tuomilehto, Antti Jula, Veikko Salomaa, Jeanette Erdmann, Christian Hengstenberg, Christina Loley, Heribert Schunkert, Claudia Lamina, H. Erich Wichmann, Eva Albrecht, Christian Gieger, Andrew A. Hicks, Åsa Johansson, Peter P. Pramstaller, Sekar Kathiresan, Elizabeth K. Speliotes, Brenda Penninx, Anna-Liisa Hartikainen, Marjo-Riitta Jarvelin, Ulf Gyllensten, Dorret I. Boomsma, Harry Campbell, James F. Wilson, Stephen J. Chanock, Martin Farrall, Anuj Goel, Carolina Medina-Gomez, Fernando Rivadeneira, Karol Estrada, André G. Uitterlinden, Albert Hofman, M. Carola Zillikens, Martin den Heijer, Lambertus A. Kiemeney, Andrea Maschio, Per Hall, Jonathan Tyrer, Alexander Teumer, Henry Völzke, Peter Kovacs, Anke Tönjes, Massimo Mangino, Tim D. Spector, Caroline Hayward, Igor Rudan, Alistair S. Hall, Nilesh J. Samani, Antony Paul Attwood, Jennifer G. Sambrook, Joseph Hung, Lyle J. Palmer, Marja-Liisa Lokki, Juha Sinisalo, Gabrielle Boucher, Heikki Huikuri, Mattias Lorentzon, Claes Ohlsson, Niina Eklund, Johan G. Eriksson, Cristina Barlassina, Carlo Rivolta, Ilja M. Nolte, Harold Snieder, Melanie M. Van der Klauw, Jana V. Van Vliet-Ostaptchouk, Pablo V. Gejman, Jianxin Shi, Kevin B. Jacobs, Zhaoming Wang, Stephan J. L. Bakker, Irene Mateo Leach, Gerjan Navis, Pim van der Harst, Nicholas G. Martin, Sarah E. Medland, Grant W. Montgomery, Jian Yang, Daniel I. Chasman, Paul M. Ridker, Lynda M. Rose, Terho Lehtimäki, Olli Raitakari, Devin Absher, Carlos Iribarren, Hanneke Basart, Kees G. Hovingh, Elina Hyppönen, Chris Power, Denise Anderson, John P. Beilby, Jennie Hui, Jennifer Jolley, Hendrik Sager, Stefan R. Bornstein, Peter E. H. Schwarz, Kati Kristiansson, Markus Perola, Jaana Lindström, Amy J. Swift, Matti Uusitupa, Mustafa Atalay, Timo A. Lakka, Rainer Rauramaa, Jennifer L. Bolton, Gerry Fowkes, Ross M. Fraser, Jackie F. Price, Krista Fischer, Kaarel KrjutÅ¡kov, Andres Metspalu, Evelin Mihailov, Claudia Langenberg, Jian'an Luan, Ken K. Ong, Peter S. Chines, Sirkka M. Keinanen-Kiukaanniemi, Timo E. Saaristo, Sarah Edkins, Paul W. Franks, Göran Hallmans, Dmitry Shungin, Andrew David Morris, Colin N. A. Palmer, Raimund Erbel, Susanne Moebus, Markus M. Nöthen, Sonali Pechlivanis, Kristian Hveem, Narisu Narisu, Anders Hamsten, Steve E. Humphries, Rona J. Strawbridge, Elena Tremoli, Harald Grallert, Barbara Thorand, Thomas Illig, Wolfgang Koenig, Martina Müller-Nurasyid, Annette Peters, Bernhard O. Boehm, Marcus E. Kleber, Winfried März, Bernhard R. Winkelmann, Johanna Kuusisto, Markku Laakso, Dominique Arveiler, Giancarlo Cesana, Kari Kuulasmaa, Jarmo Virtamo, John W. G. Yarnell, Diana Kuh, Andrew Wong, Lars Lind, Ulf de Faire, Bruna Gigante, Patrik K. E. Magnusson, Nancy L. Pedersen, George Dedoussis, Maria Dimitriou, Genovefa Kolovou, Stavroula Kanoni, Kathleen Stirrups, Lori L. Bonnycastle, Inger Njølstad, Tom Wilsgaard, Andrea Ganna, Emil Rehnberg, Aroon Hingorani, Mika Kivimaki, Meena Kumari, Themistocles L. Assimes, Inês Barroso, Michael Boehnke, Ingrid B. Borecki, Panos Deloukas, Caroline S. Fox, Timothy Frayling, Leif C. Groop, Talin Haritunians, David Hunter, Erik Ingelsson, Robert Kaplan, Karen L. Mohlke, Jeffrey R. O'Connell, David Schlessinger, David P. Strachan, Kari Stefansson, Cornelia M. van Duijn, Gonçalo R. Abecasis, Mark I. McCarthy, Joel N. Hirschhorn, Lu Qi, Ruth J. F. Loos, Cecilia M. Lindgren, Kari E. North, Iris M. Heid

**Affiliations:** 1 Wellcome Trust Sanger Institute, Hinxton, Cambridge, United Kingdom; 2 Wellcome Trust Centre for Human Genetics, University of Oxford, Oxford, United Kingdom; 3 Department of Genetic Epidemiology, Institute of Epidemiology and Preventive Medicine, University of Regensburg, Regensburg, Germany; 4 Department of Medical Genetics, University of Lausanne, Lausanne, Switzerland; 5 Swiss Institute of Bioinformatics, Lausanne, Switzerland; 6 Division of Cancer Epidemiology and Genetics, National Cancer Institute, National Institutes of Health, Department of Health and Human Services, Bethesda, Maryland, United States of America; 7 Department of Biostatistics, Center for Statistical Genetics, University of Michigan, Ann Arbor, Michigan, United States of America; 8 Department of Epidemiology, School of Public Health, University of North Carolina at Chapel Hill, Chapel Hill, North Carolina, United States of America; 9 MRC Epidemiology Unit, Institute of Metabolic Science, Addenbrooke's Hospital, Cambridge, United Kingdom; 10 Estonian Genome Center, University of Tartu, Tartu, Estonia; 11 Institute of Molecular and Cell Biology, University of Tartu, Tartu, Estonia; 12 Department of Epidemiology, Tulane School of Public Health and Tropical Medicine, New Orleans, Louisiana, United States of America; 13 Department of Nutrition, Harvard School of Public Health, Boston, Massachusetts, United States of America; 14 Department of Genetics, Washington University School of Medicine, St. Louis, Missouri, United States of America; 15 Department of Genetics, University of North Carolina, Chapel Hill, North Carolina, United States of America; 16 Department of Medical Epidemiology and Biostatistics, Karolinska Institutet, Stockholm, Sweden; 17 Institute for Medical Informatics, Biometry and Epidemiology (IMIBE), University Hospital of Essen, University of Duisburg-Essen, Essen, Germany; 18 Divisions of Genetics and Endocrinology and Program in Genomics, Children's Hospital, Boston, Massachusetts, United States of America; 19 Metabolism Initiative and Program in Medical and Population Genetics, Broad Institute, Cambridge, Massachusetts, United States of America; 20 Department of Genetics, Harvard Medical School, Boston, Massachusetts, United States of America; 21 Genetics of Complex Traits, Peninsula College of Medicine and Dentistry, University of Exeter, Exeter, United Kingdom; 22 Department of Epidemiology, Harvard School of Public Health, Boston, Massachusetts, United States of America; 23 Department of Biostatistics, Harvard School of Public Health, Boston, Massachusetts, United States of America; 24 deCODE Genetics, Reykjavik, Iceland; 25 Department of Genetic Medicine and Development, University of Geneva Medical School, Geneva, Switzerland; 26 Biomedical Sciences Research Center Al. Fleming, Vari, Greece; 27 Oxford Centre for Diabetes, Endocrinology and Metabolism, University of Oxford, Oxford, United Kingdom; 28 Department of Statistics, University of Oxford, Oxford, United Kingdom; 29 MRC Harwell, Harwell, United Kingdom; 30 University of Texas Southwestern Medical Center, Dallas, Texas, United States of America; 31 Regensburg University Medical Center, Innere Medizin I, Regensburg, Germany; 32 CNRS UMR8199-IBL-Institut Pasteur de Lille, Lille, France; 33 Department of Genomics of Common Disease, School of Public Health, Imperial College London, London, United Kingdom; 34 Department of Biological Psychology, VU University Amsterdam, Amsterdam, The Netherlands; 35 Hudson Alpha Institute for Biotechnology, Huntsville, Alabama, United States of America; 36 Laboratory of Epidemiology, Demography, Biometry, National Institute on Aging, National Institutes of Health, Bethesda, Maryland, United States of America; 37 Icelandic Heart Association, Kopavogur, Iceland; 38 University of Iceland, Reykjavik, Iceland; 39 Department of Medicine, University of Maryland School of Medicine, Baltimore, Maryland, United States of America; 40 Geriatrics Research and Education Clinical Center, Baltimore Veterans Administration Medical Center, Baltimore, Maryland, United States of America; 41 School of Social and Community Medicine, University of Bristol, Bristol, United Kingdom; 42 Clinical Pharmacology and Barts and The London Genome Centre, William Harvey Research Institute, Barts and The London School of Medicine and Dentistry, Queen Mary University of London, London, United Kingdom; 43 Department of OB/GYN and Medical Genetics Institute, Cedars-Sinai Medical Center, Los Angeles, California, United States of America; 44 Department of Medicine, David Geffen School of Medicine at University of California, Los Angeles, California, United States of America; 45 Cardiovascular Health Research Unit, University of Washington, Seattle, Washington, United States of America; 46 Service of Medical Genetics, Centre Hospitalier Universitaire Vaudois (CHUV) University Hospital, Lausanne, Switzerland; 47 Faculty of Medicine, University of Iceland, Reykjavík, Iceland; 48 Department of Public Health and Primary Care, Institute of Public Health, University of Cambridge, Cambridge, United Kingdom; 49 Department of Epidemiology, Erasmus MC, Rotterdam, The Netherlands; 50 Department of Clinical Genetics, Erasmus MC, Rotterdam, The Netherlands; 51 Centre for Medical Systems Biology & Netherlands Consortium on Healthy Aging, Leiden, The Netherlands; 52 Netherlands Genomics Initiative (NGI)-sponsored Netherlands Consortium for Healthy Aging (NCHA), Leiden, The Netherlands; 53 Department of Biostatistics, Boston University School of Public Health, Boston, Massachusetts, United States of America; 54 Department of Neurology, Boston University School of Medicine, Boston, Massachusetts, United States of America; 55 National Institute for Health and Welfare, Unit for Child and Adolescent Psychiatry, Helsinki, Finland; 56 Finnish Twin Cohort Study, Department of Public Health, University of Helsinki, Helsinki, Finland; 57 Institute for Molecular Medicine Finland (FIMM), University of Helsinki, Helsinki, Finland; 58 National Institute for Health and Welfare, Department of Chronic Disease Prevention, Unit of Public Health Genomics, Helsinki, Finland; 59 Genome Technology Branch, National Human Genome Research Institute, NIH, Bethesda, Maryland, United States of America; 60 South Karelia Central Hospital, Lappeenranta, Finland; 61 Red RECAVA Grupo RD06/0014/0015, Hospital Universitario, La Paz, Madrid, Spain; 62 Centre for Vascular Prevention, Danube-University Krems, Krems, Austria; 63 National Institute for Health and Welfare, Diabetes Prevention Unit, Helsinki, Finland; 64 South Ostrobothnia Central Hospital, Seinajoki, Finland; 65 National Institute for Health and Welfare, Department of Chronic Disease Prevention, Population Studies Unit, Turku, Finland; 66 National Institute for Health and Welfare, Department of Chronic Disease Prevention, Chronic Disease Epidemiology and Prevention Unit, Helsinki, Finland; 67 Nordic Center of Cardiovascular Research (NCCR), Lübeck, Germany; 68 Universität zu Lübeck, Medizinische Klinik II, Lübeck, Germany; 69 Institut für Medizinische Biometrie und Statistik, Universität zu Lübeck, Universitätsklinikum Schleswig-Holstein, Campus Lübeck, Lübeck, Germany; 70 Deutsches Herzzentrum München and DZHK (German Center for Cardiovascular Research), partner site Munich Heart Alliance, Munich, Germany; 71 Division of Genetic Epidemiology, Department of Medical Genetics, Molecular and Clinical Pharmacology, Innsbruck Medical University, Innsbruck, Austria; 72 Institute of Epidemiology I, Helmholtz Zentrum München - German Research Center for Environmental Health, Neuherberg, Germany; 73 Institute of Medical Informatics, Biometry and Epidemiology, Chair of Epidemiology, Ludwig-Maximilians-Universität, and Klinikum Grosshadern, Munich, Germany; 74 Institute of Genetic Epidemiology, Helmholtz Zentrum München - German Research Center for Environmental Health, Neuherberg, Germany; 75 Center for Biomedicine, European Academy Bozen/Bolzano (EURAC), Bolzano/Bozen, Italy, Affiliated Institute of the University of Lübeck, Lübeck, Germany; 76 Department of Immunology, Genetics and Pathology, Uppsala University, Uppsala, Sweden; 77 Uppsala Clinical Research Center, Uppsala University Hospital, Uppsala, Sweden; 78 Department of Neurology, General Central Hospital, Bolzano, Italy; 79 Department of Neurology, University of Lübeck, Lübeck, Germany; 80 Cardiovascular Research Center and Cardiology Division, Massachusetts General Hospital, Boston, Massachusetts, United States of America; 81 Center for Human Genetic Research, Massachusetts General Hospital, Boston, Massachusetts, United States of America; 82 Program in Medical and Population Genetics, Broad Institute of Harvard and Massachusetts Institute of Technology, Cambridge, Massachusetts, United States of America; 83 Center for Computational Medicine and Bioinformatics, University of Michigan, Ann Arbor, Michigan, United States of America; 84 Department of Internal Medicine, Division of Gastroenterology, University of Michigan, Ann Arbor, Michigan, United States of America; 85 Department of Psychiatry, University Medical Centre Groningen, Groningen, The Netherlands; 86 Department of Clinical Sciences/Obstetrics and Gynecology, University of Oulu, Oulu, Finland; 87 Department of Epidemiology and Biostatistics, School of Public Health, Faculty of Medicine, Imperial College London, London, United Kingdom; 88 Institute of Health Sciences, University of Oulu, Oulu, Finland; 89 Biocenter Oulu, University of Oulu, Oulu, Finland; 90 National Institute for Health and Welfare, Oulu, Finland; 91 Centre for Population Health Sciences, University of Edinburgh, Edinburgh, United Kingdom; 92 Cardiovascular Medicine, University of Oxford, Wellcome Trust Centre for Human Genetics, Oxford, United Kingdom; 93 Department of Internal Medicine, Erasmus MC, Rotterdam, The Netherlands; 94 Department of Internal Medicine, VU University Medical Centre, Amsterdam, The Netherlands; 95 Department of Epidemiology, Biostatistics and HTA, Radboud University Nijmegen Medical Centre, Nijmegen, The Netherlands; 96 Department of Urology, Radboud University Nijmegen Medical Centre, Nijmegen, The Netherlands; 97 Comprehensive Cancer Center East, Nijmegen, The Netherlands; 98 Istituto di Neurogenetica e Neurofarmacologia del CNR, Monserrato, Cagliari, Italy; 99 Department of Oncology, University of Cambridge, Cambridge, United Kingdom; 100 Interfaculty Institute for Genetics and Functional Genomics, Ernst-Moritz-Arndt-University Greifswald, Greifswald, Germany; 101 Institute for Community Medicine, Ernst-Moritz-Arndt-University Greifswald, Greifswald, Germany; 102 Interdisciplinary Centre for Clinical Research, University of Leipzig, Leipzig, Germany; 103 University of Leipzig, IFB Adiposity Diseases, Leipzig, Germany; 104 Department of Medicine, University of Leipzig, Leipzig, Germany; 105 Department of Twin Research and Genetic Epidemiology, King's College London, London, United Kingdom; 106 MRC Human Genetics Unit, Institute for Genetics and Molecular Medicine, Western General Hospital, Edinburgh, United Kingdom; 107 Division of Cardiovascular and Neuronal Remodelling, Multidisciplinary Cardiovascular Research Centre, Leeds Institute of Genetics, Health and Therapeutics, University of Leeds, Leeds, United Kingdom; 108 Department of Cardiovascular Sciences, University of Leicester, Glenfield Hospital, Leicester, United Kingdom; 109 Leicester NIHR Biomedical Research Unit in Cardiovascular Disease, Glenfield Hospital, Leicester, United Kingdom; 110 Department of Haematology, University of Cambridge, Cambridge, United Kingdom; 111 NHS Blood and Transplant, Cambridge Centre, Cambridge, United Kingdom; 112 School of Medicine and Pharmacology, The University of Western Australia, Nedlands, Western Austrailia, Australia; 113 Busselton Population Medical Research Foundation Inc., Sir Charles Gairdner Hospital, Nedlands, Western Australia, Australia; 114 Genetic Epidemiology and Biostatistics Platform, Ontario Institute for Cancer Research, Toronto, Canada; 115 Prosserman Centre for Health Research, Samuel Lunenfeld Research Institute, Toronto, Canada; 116 Transplantation Laboratory, Haartman Institute, University of Helsinki, Helsinki, Finland; 117 Division of Cardiology, Cardiovascular Laboratory, Helsinki University Central Hospital, Helsinki, Finland; 118 Montreal Heart Institute, Montreal, Quebec, Canada; 119 Institute of Clinical Medicine, Department of Internal Medicine, University of Oulu, Oulu, Finland; 120 Department of Internal Medicine, Institute of Medicine, Sahlgrenska Academy, University of Gothenburg, Gothenburg, Sweden; 121 Department of General Practice and Primary Health Care, University of Helsinki, Helsinki, Finland; 122 National Institute for Health and Welfare, Helsinki, Finland; 123 Helsinki University Central Hospital, Unit of General Practice, Helsinki, Finland; 124 University of Milan, Department of Medicine, Surgery and Dentistry, Milano, Italy; 125 Unit of Genetic Epidemiology and Bioinformatics, Department of Epidemiology, University Medical Center Groningen, University of Groningen, Groningen, The Netherlands; 126 LifeLines Cohort Study, University Medical Center Groningen, University of Groningen, Groningen, The Netherlands; 127 Department of Endocrinology, University Medical Center Groningen, University of Groningen, Groningen, The Netherlands; 128 University of Chicago, Chicago, Illinois, United States of America; 129 Northshore University Healthsystem, Evanston, Ilinois, United States of America; 130 Core Genotyping Facility, SAIC-Frederick, Inc., NCI-Frederick, Frederick, Maryland, United States of America; 131 Department of Internal Medicine, University Medical Center Groningen, University of Groningen, Groningen, The Netherlands; 132 Department of Cardiology, University Medical Center Groningen, University of Groningen, Groningen, The Netherlands; 133 Department of Genetics, University Medical Center Groningen, University of Groningen, Groningen, The Netherlands; 134 Genetic Epidemiology Laboratory, Queensland Institute of Medical Research, Queensland, Australia; 135 Molecular Epidemiology Laboratory, Queensland Institute of Medical Research, Queensland, Australia; 136 Queensland Statistical Genetics Laboratory, Queensland Institute of Medical Research, Queensland, Australia; 137 Division of Preventive Medicine, Brigham and Women's Hospital, Boston, Massachusetts, United States of America; 138 Harvard Medical School, Boston, Massachusetts, United States of America; 139 Department of Clinical Chemistry, University of Tampere and Tampere University Hospital, Tampere, Finland; 140 Research Centre of Applied and Preventive Cardiovascular Medicine, University of Turku, Turku, Finland; 141 The Department of Clinical Physiology, Turku University Hospital, Turku, Finland; 142 Division of Research, Kaiser Permanente Northern California, Oakland, California, United States of America; 143 Department of Vascular Medicine, Academic Medical Center, Amsterdam, The Netherlands; 144 Centre For Paediatric Epidemiolgy and Biostatistics/MRC Centre of Epidemiology for Child Health, University College of London Institute of Child Health, London, United Kingdom; 145 Telethon Institute for Child Health Research, West Perth, Western Australia, Australia; 146 Centre for Child Health Research, The University of Western Australia, Perth, Australia; 147 PathWest Laboratory of Western Australia, Department of Molecular Genetics, QEII Medical Centre, Nedlands, Western Australia, Australia; 148 School of Pathology and Laboratory Medicine, University of Western Australia, Nedlands, Western Australia, Australia; 149 School of Population Health, The University of Western Australia, Nedlands, Western Austrailia, Australia; 150 Medizinische Klinik II, Universität zu Lübeck, Lübeck, Germany; 151 Department of Medicine III, University of Dresden, Medical Faculty Carl Gustav Carus, Dresden, Germany; 152 Department of Public Health and Clinical Nutrition, University of Eastern Finland, Kuopio, Finland; 153 Research Unit, Kuopio University Hospital, Kuopio, Finland; 154 Institute of Biomedicine/Physiology, University of Eastern Finland, Kuopio Campus, Kuopio, Finland; 155 Kuopio Research Institute of Exercise Medicine, Kuopio, Finland; 156 Department of Clinical Physiology and Nuclear Medicine, Kuopio University Hospital, Kuopio, Finland; 157 Department of Epidemiology and Public Health, University College London, London, United Kingdom; 158 MRC Unit for Lifelong Health & Ageing, London, United Kingdom; 159 Faculty of Medicine, Institute of Health Sciences, University of Oulu, Oulu, Finland; 160 Unit of General Practice, Oulu University Hospital, Oulu, Finland; 161 Finnish Diabetes Association, Tampere, Finland; 162 Pirkanmaa Hospital District, Tampere, Finland; 163 Department of Clinical Sciences, Genetic and Molecular Epidemiology Unit, Skåne University Hospital Malmö, Lund University, Malmö, Sweden; 164 Department of Nutrition, Harvard School of Public Health, Boston, Massachusetts, United States of America; 165 Department of Public Health & Clinical Medicine, Umeå University,Umeå, Sweden; 166 Department of Odontology, Umeå University, Umea, Sweden; 167 Medical Research Institute, University of Dundee, Ninewells Hospital and Medical School, Dundee, United Kingdom; 168 Clinic of Cardiology, West German Heart Centre, University Hospital of Essen, University Duisburg-Essen, Essen, Germany; 169 Institute of Human Genetics, University of Bonn, Bonn, Germany; 170 Department of Genomics, Life & Brain Center, University of Bonn, Bonn, Germany; 171 HUNT Research Centre, Department of Public Health and General Practice, Norwegian University of Science and Technology, Levanger, Norway; 172 Atherosclerosis Research Unit, Department of Medicine, Solna, Karolinska Institutet, Karolinska University Hospital, Stockholm, Sweden; 173 Cardiovascular Genetics, British Heart Foundation Laboratories, Rayne Building, University College London, London, United Kingdom; 174 Department of Pharmacological Sciences, University of Milan, Monzino Cardiology Center, IRCCS, Milan, Italy; 175 Unit for Molecular Epidemiology, Helmholtz Zentrum München - German Research Center for Environmental Health, Neuherberg, Germany; 176 Institute of Epidemiology II, Helmholtz Zentrum München - German Research Center for Environmental Health, Neuherberg, Germany; 177 Hannover Unified Biobank, Hannover Medical School, Hannover, Germany; 178 Department of Internal Medicine II – Cardiology, University of Ulm Medical Center, Ulm, Germany; 179 Department of Medicine I, University Hospital Grosshadern, Ludwig-Maximilians-Universität, Munich, Germany; 180 Institute of Medical Informatics, Biometry and Epidemiology, Chair of Genetic Epidemiology, Ludwig-Maximilians-Universität, Munich, Germany; 181 Division of Endocrinology and Diabetes, Department of Medicine, University Hospital, Ulm, Germany; 182 LURIC Study nonprofit LLC, Freiburg, Germany; 183 Mannheim Institute of Public Health, Social and Preventive Medicine, Medical Faculty of Mannheim, University of Heidelberg, Mannheim, Germany; 184 Synlab Academy, Mannheim, Germany; 185 Cardiology Group, Frankfurt-Sachsenhausen, Germany; 186 Department of Medicine, University of Kuopio and Kuopio University Hospital, Kuopio, Finland; 187 Department of Epidemiology and Public Health, Faculty of Medicine, Strasbourg, France; 188 Department of Clinical Medicine, University of Milano-Bicocca, Monza, Italy; 189 Centre for Public Health, Queen's University, Belfast, United Kingdom; 190 Department of Medical Sciences, Uppsala University, Akademiska Sjukhuset, Uppsala, Sweden; 191 Division of Cardiovascular Epidemiology, Institute of Environmental Medicine, Karolinska Institutet, Stockholm, Sweden; 192 Department of Dietetics-Nutrition, Harokopio University, Athens, Greece; 193 1st Cardiology Department, Onassis Cardiac Surgery Center, Athens, Greece; 194 Department of Community Medicine, Faculty of Health Sciences, University of Tromsø, Tromsø, Norway; 195 Department of Medicine, Stanford University School of Medicine, Stanford, California, United States of America; 196 University of Cambridge Metabolic Research Labs, Institute of Metabolic Science Addenbrooke's Hospital, Cambridge, United Kingdom; 197 Division of Intramural Research, National Heart, Lung and Blood Institute, Framingham Heart Study, Framingham, Massachusetts, United States of America; 198 Lund University Diabetes Centre, Department of Clinical Sciences, Lund University, Malmö, Sweden; 199 Medical Genetics Institute, Cedars-Sinai Medical Center, Los Angeles, California, United States of America; 200 Channing Laboratory, Department of Medicine, Brigham and Women's Hospital and Harvard Medical School, Boston, Massachusetts, United States of America; 201 Department of Epidemiology and Population Health, Albert Einstein College of Medicine, Bronx, New York, United States of America; 202 Laboratory of Genetics, National Institute on Aging, Baltimore, Maryland, United States of America; 203 Division of Community Health Sciences, St George's, University of London, London, United Kingdom; 204 Center of Medical Systems Biology, Leiden University Medical Center, Leiden, The Netherlands; 205 Oxford National Institute for Health Research Biomedical Research Centre, Churchill Hospital, Oxford, United Kingdom; 206 Genetics of Obesity and Related Metabolic Traits Program,The Charles Bronfman Institute of Personalized Medicine, Child Health and Development Institute, Mount Sinai School of Medicine, New York, New York, United States of America; Georgia Institute of Technology, United States of America

## Abstract

Given the anthropometric differences between men and women and previous evidence of sex-difference in genetic effects, we conducted a genome-wide search for sexually dimorphic associations with height, weight, body mass index, waist circumference, hip circumference, and waist-to-hip-ratio (133,723 individuals) and took forward 348 SNPs into follow-up (additional 137,052 individuals) in a total of 94 studies. Seven loci displayed significant sex-difference (FDR<5%), including four previously established (near *GRB14/COBLL1*, *LYPLAL1/SLC30A10*, *VEGFA*, *ADAMTS9*) and three novel anthropometric trait loci (near *MAP3K1*, *HSD17B4*, *PPARG*), all of which were genome-wide significant in women (P<5×10^−8^), but not in men. Sex-differences were apparent only for waist phenotypes, not for height, weight, BMI, or hip circumference. Moreover, we found no evidence for genetic effects with opposite directions in men versus women. The *PPARG* locus is of specific interest due to its role in diabetes genetics and therapy. Our results demonstrate the value of sex-specific GWAS to unravel the sexually dimorphic genetic underpinning of complex traits.

## Author Summary

Men and women differ substantially regarding height, weight, and body fat. Interestingly, previous work detecting genetic effects for waist-to-hip ratio, to assess body fat distribution, has found that many of these showed sex-differences. However, systematic searches for sex-differences in genetic effects have not yet been conducted. Therefore, we undertook a genome-wide search for sexually dimorphic genetic effects for anthropometric traits including 133,723 individuals in a large meta-analysis and followed promising variants in further 137,052 individuals, including a total of 94 studies. We identified seven loci with significant sex-difference including four previously established (near *GRB14/COBLL1*, *LYPLAL1/SLC30A10*, *VEGFA*, *ADAMTS9*) and three novel anthropometric trait loci (near *MAP3K1*, *HSD17B4*, *PPARG*), all of which were significant in women, but not in men. Of interest is that sex-difference was only observed for waist phenotypes, but not for height or body-mass-index. We found no evidence for sex-differences with opposite effect direction for men and women. The *PPARG* locus is of specific interest due to its link to diabetes genetics and therapy. Our findings demonstrate the importance of investigating sex differences, which may lead to a better understanding of disease mechanisms with a potential relevance to treatment options.

## Introduction

Height, fat mass, and fat distribution differ substantially between men and women, and these differences may, in part, explain the sex-specific susceptibilities to certain diseases [1], [2]. A subtle sexual dimorphism in body composition is already apparent during childhood, and emerges more prominently during adolescence as boys start exceeding girls with regard to height and muscle mass, while girls accumulate more fat mass [3]–[5]. These considerable differences in anthropometry may reflect sex-specific differences in steroid hormone regulation, adipogenesis, lipid storage, muscle metabolism, composition, and contractile speed, skeletal growth and maturation, or lipolysis, and suggest a genetic underpinning [1], [2], [6]–[10].

While direct measures of height or weight are easily obtained, measures of fat mass and fat distribution are more invasive and less frequently assessed in large-scale epidemiological studies. Instead, body mass index (BMI, computed as weight/height^2^) is used to assess overall adiposity, whereas waist-to-hip ratio (WHR) is a measure of fat distribution. Increased WHR is suggestive of more preferential accumulation of fat around the waist versus the hip. Obesity (defined as a BMI≥30 kg/m^2^) is a well-established risk factor for type 2 diabetes, cardiovascular disease, cancer and mortality [11]–[18]. Also the independent effect of WHR – derived by computing WHR adjusted for BMI - on morbidity and mortality has been demonstrated [19], [20]. Thus, anthropometric measures depict not only body size, but also fat distribution and consequently various facets of chronic disease risk.

Genome-wide association studies (GWAS) have successfully identified many genetic loci robustly associated with height [21]–[25], body mass index (BMI) [26]–[29], and waist-to-hip ratio (WHR) [30], . So far, all GWAS for anthropometric traits have been performed in men and women combined. However, in our most recent GWAS of WHR within the Genetic Investigation of ANthropometric Traits (GIANT) consortium, we found that seven of the 14 novel loci displayed more pronounced effects in women than in men, when we subsequently stratified analyses by sex [31]. In contrast, our GWAS for BMI or height genetic effects with GIANT, no sex-differences in the newly identified loci were noted [25], [29]. However, these GWAS did not specifically aim to identify genetic loci with sex-specific effects such that a systematic search for such sexually dimorphic loci was warranted.

Thus, given the obvious difference in physical appearance between men and women in body size and shape, together with the strong evidence of sex-specific effects of the recently identified WHR loci, we performed a systematic search for sex-specific loci for anthropometric traits. GWAS conducted separately in men and women not only improve power to identify sex-sensitive associations, but also allow testing for sex differences. Within the Genetic Investigation of ANthropometric Traits (GIANT) consortium, we performed sex-specific GWAS for six anthropometric traits involving a total of 270,775 subjects from 94 studies in order to investigate the extent and nature of sex-specific genetic effects on anthropometry.

## Results

### Discovery meta-analysis of sex-specific GWAS for anthropometric traits

In the discovery stage, sex-specific GWA analyses were conducted in 46 studies (Table S1), including up to 60,586 men and 73,137 women, testing ∼2.8 million autosomal SNPs for association with six anthropometric traits that are well established to represent body size and shape: i.e. height, weight, BMI, waist circumference (WC), hip circumference (HIP), and WHR. In order to capture body fat distribution independent of overall adiposity, the latter three traits were also analyzed with adjustment for BMI (WCadjBMI, HIPadjBMI, WHRadjBMI) yielding nine phenotypes n total (Methods). Study-specific information has been described previously [25], [29], [31] and details on study-specific analyses are given in Methods. All study participants were of European and European American descent. We performed an inverse-variance weighted fixed-effects meta-analysis for each of the 18 analyses (9 phenotypes, 2 sexes; Methods) yielding meta-analyzed sex-specific P-values for association (*P-men*, *P-women*) and corresponding sex-specific effect estimates. In order to account for multiple testing across SNPs genome-wide as well as across phenotypes, we applied a false-discovery-rate (FDR) approach [32].

Generally, in order to establish a sexually dimorphic association, we require both a significant SNP association with an anthropometric trait at least in one sex (*P-men* or *P-women* at 5% FDR across all SNPs and phenotypes tested) and a significant sex-difference of a SNP (P-value testing for difference in sex-specific effect estimates, *P-diff*, at 5% FDR). Sexually dimorphic SNPs could either show (i) concordant effect direction (CED), if the association is present in one sex (*P-men* or *P-women* at 5% FDR) and at least nominally significant and directionally concordant in the other (*P-women* or *P-men*<0.05, respectively), (ii) single sex effect (SSE), if the association is present in one sex and not significant in the other, or (iii) opposite effect direction (OED), if the association is present in one sex and at least nominally significant in the opposite direction in the other sex. We aimed to identify genetic loci with CED or SSE, which are biologically most plausible. Nevertheless, in this exploratory effort, we also searched for OED loci – which are biologically unlikely, but current lack of knowledge of such signals could be due to the fact that current GWAS of men and women combined cannot detect such signals.

We evaluated the power of two genome-wide approaches to screen for sex-sensitive genetic loci: (a) a scan for sex-specific association P-values in men and women separately (*P-men*, *P-women*, sex-specific scan) and (b) a scan for P-values testing for sex difference between effects of men and women (*P-diff*, sex-difference scan; details in Methods). Power calculations showed that the sex-specific scan had a higher probability to select SNPs with true underlying CED or SSE signal into follow-up, while the sex-difference scan had a higher probability to select SNPs with true underlying OED effect (details in Text S1). We thus conducted both scans.

**Figure 1:**
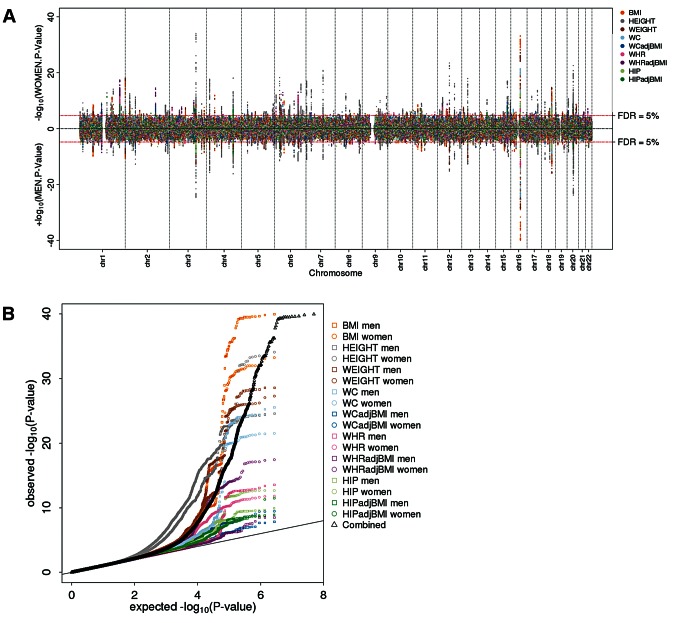
Genome-wide scan for sex-specific genome-wide association highlights numerous loci. (a) Manhattan plot showing the men-specific (upward, up to 60,586 men) and women-specific (downward, up to 73,137 women) association P-values from the discovery with the 619 selected loci colored by the phenotype for which the locus was selected; (b) QQ-plot showing the sex-specific association P-values as observed against those expected under the null overall phenotypes (black) and for each phenotypes separately (colored).

**Figure 2:**
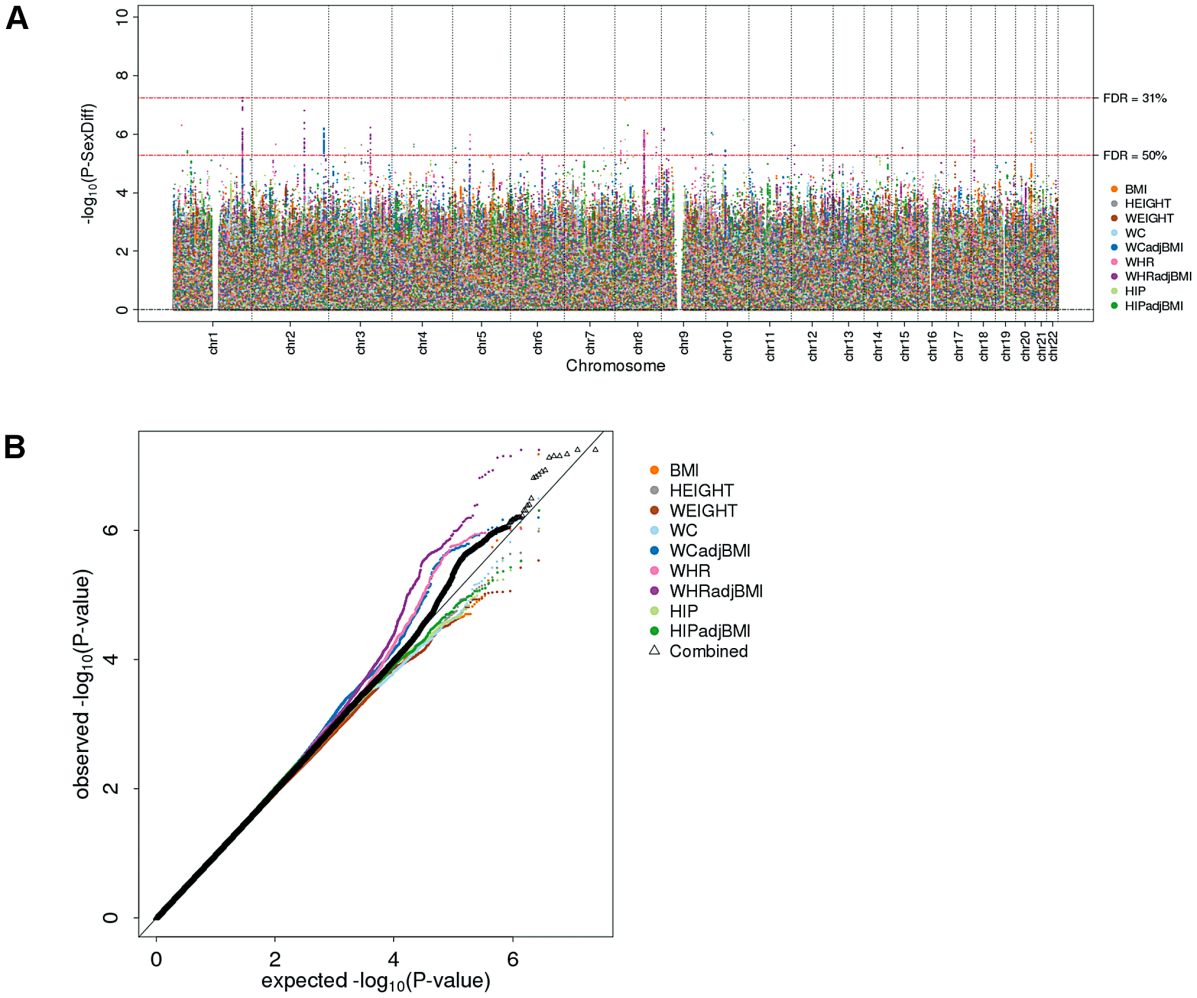
Genome-wide sex-difference scan fails to pinpoint loci. (a) Manhattan plot showing sex-difference P-values, (b) QQ plot for sex-difference P-values overall phenotypes (black) and for each phenotype separately (colored).

The sex-specific scan showed an excess of small P-values (Figure 1a, b). Controlling for 5% FDR (across all SNPs, nine phenotypes, two sexes; corresponding to a P-value of 2×10^−5^), this scan yielded 619 independent SNPs associated with at least one of the phenotypes in at least one of the sexes. Including a rough filter for sex-difference (nominal significant, *P-diff*<0.05), we took 348 out of these 619 SNPs forward for follow-up (73 SNPs for height, 28 for weight, 32 for BMI, 31 for WC, 46 for WCadjBMI, 33 for HIP, 38 for HIPadjBMI, 28 for WHR, and 39 for WHRadjBMI; Table S2). The sex-difference scan did not identify any SNPs at 5% FDR, despite the fact that the QQ-plot for all phenotypes combined as well as for phenotype-specific traits indicated some deviation of the observed *P-diff* distribution from the expected (under the null hypothesis of no sex-difference) for the waist phenotypes (WHRadjBMI, WHR, WCadjBMI) (Figure 2a,b). Indeed, even if we were to carry forward SNPs at 30% FDR, we would not have identified any significant OED effects. As such, no SNPs were taken forward from this second scan for follow-up.

### Follow-up of 348 SNPs reveals seven sexually dimorphic anthropometric trait loci

In the follow up, we examined the 348 SNPs for the phenotype that the SNP was selected for in 18 studies with *in-silico* genotype information (up to 20,340 men and 41,872 women) and in 30 studies with Metabochip data (up to 42,055 men and 32,785 women; which contained assays for 43% of selected SNPs prioritized for follow-up). Study-specific information is given in Tables S1, S3, S4A,S4B, S5 and Methods. Meta-analyses of the follow-up studies as well as jointly with discovery studies were conducted for each sex separately (*P-women*, *P-men*) and both combined (*P*, Methods).

As all 348 SNPs were derived from the sex-specific discovery scan, the follow-up was then used to establish unbiased estimates of sex-difference in an independent data set (Methods). We filtered SNPs with a main effect (P-value for association combined in men and women <0.01; Methods). This yielded 74 SNPs, which were subsequently tested for sex-difference. Seven of these 74 SNPs reached a significant sex-difference at 5% FDR (six for WHRadjBMI, one for WCadjBMI, Table 1). For these seven SNPs, the *P-diff jointly* for the discovery and follow-up ranged from 2.7×10^−4^ to 6.2×10^−16^ and the joint discovery and follow-up association P-value in the predominant sex – interestingly, all in women – was genome-wide significant (*P-women*<5×10^−8^). Effect sizes were similar when we restricted our follow-up analyses to population-based studies or control-only series in order to eliminate a potential bias by patient groups (Figure S1).

**Table 1:** Seven SNPs show sex difference.

				Discovery			Follow-up			Joint				
				MEN	WOMEN	Sex-Diff	MEN	WOMEN	Sex-Diff	MEN	WOMEN	Sex-Diff	MEN	WOMEN
SNP	Trait^a^	Sex^a^	Gene^b^	P	P	P	P^c^	P^c^	P^c^	P	P	P	N	N
rs6717858	WHRadjBMI	W	*GRB14/COBLL1*	0.309	2.78E-15	6.49E-07	0.965	3.64E-16	1.08E-11	0.613	1.99E-29	6.18E-16	76,594	98,321
rs2820443	WHRadjBMI	W	*LYPLAL1/SLC30A10*	0.191	3.69E-18	1.25E-07	0.532	9.15E-21	2.60E-10	0.374	4.62E-37	6.95E-16	76,625	98,352
rs1358980	WHRadjBMI	W	*VEGFA*	0.110	1.11E-13	3.02E-05	0.112	1.38E-19	4.53E-08	0.048	2.41E-31	2.46E-11	75,703	97,269
rs11743303	WCadjBMI	W	*MAP3K1*	0.974	2.27E-06	6.24E-04	0.172	7.15E-07	5.35E-05	0.570	2.69E-11	8.41E-07	85,136^d^	107,403^d^
rs2371767	WHRadjBMI	W	*ADAMTS9*	0.196	1.63E-08	1.85E-03	6.08E-03	8.55E-17	2.14E-04	8.34E-03	7.07E-23	1.91E-06	72,649	95,325
rs10478424	WHRadjBMI	W	*HSD17B4*	0.399	1.02E-05	9.84E-03	0.864	3.81E-05	1.67E-03	0.761	3.45E-09	2.66E-04	43,852^e^	73,066^e^
rs4684854	WHRadjBMI	W	*PPARG*	0.955	2.36E-08	6.46E-05	0.132	1.48E-07	4.22E-03	0.411	4.17E-14	4.04E-06	74,652	96,472

a Trait and sex for which the SNP was selected;

b Gene labels state the nearest gene or the gene as published previously; details on all genes near the association signal can be found in the Figure S2;

c One-sided P-Values.

d larger sample size due to one additional study that did not have hip circumference, and therefore could not contribute to WHRadjBMI.

e smaller sample size as this SNP was not on Metabochip.

Shown are the seven SNPs with significant (at 5% false discovery rate) sex difference in the follow-up data. These seven SNPs exhibit genome-wide significant association in women (joint discovery and follow-up *P_women*<5×10−8) and only two of these show nominally significant association in men (joint *P_men*<0.05). The three loci MAP3K1, HSD17B4, and PPARG are shown here for the first time for their anthropometric trait association as well as for sex-difference.

### The seven confirmed sex-difference loci include three novel signals

We found that three of these seven identified loci describe novel associations with WCadjBMI (near *MAP3K1*) or WHRadjBMI (near *HSD17B4* and *PPARG*) that were genome-wide significant in women (joint *P-women*: 3.4×10^−9^ to 4.2×10^−14^), but not in men (joint *P-men*: 0.41 to 0.76), whereas the remaining four had been established previously (ref). These three novel loci would have been missed by sex-combined scans at 5% FDR (equivalent to P>5.8×10^−5^).

Of particular interest is the *PPARG* region, which we identified for the first time as a locus for anthropometric traits (WHRadjBMI) in the context of a genome-wide study and with evidence for a women-specific association. *PPARG* is of considerable importance due to its function as a nuclear hormone receptor with specific known interaction with sex hormones, for example with estrogen receptors [33], and due to its role in type 2 diabetes development and therapy.

**Figure 3:**
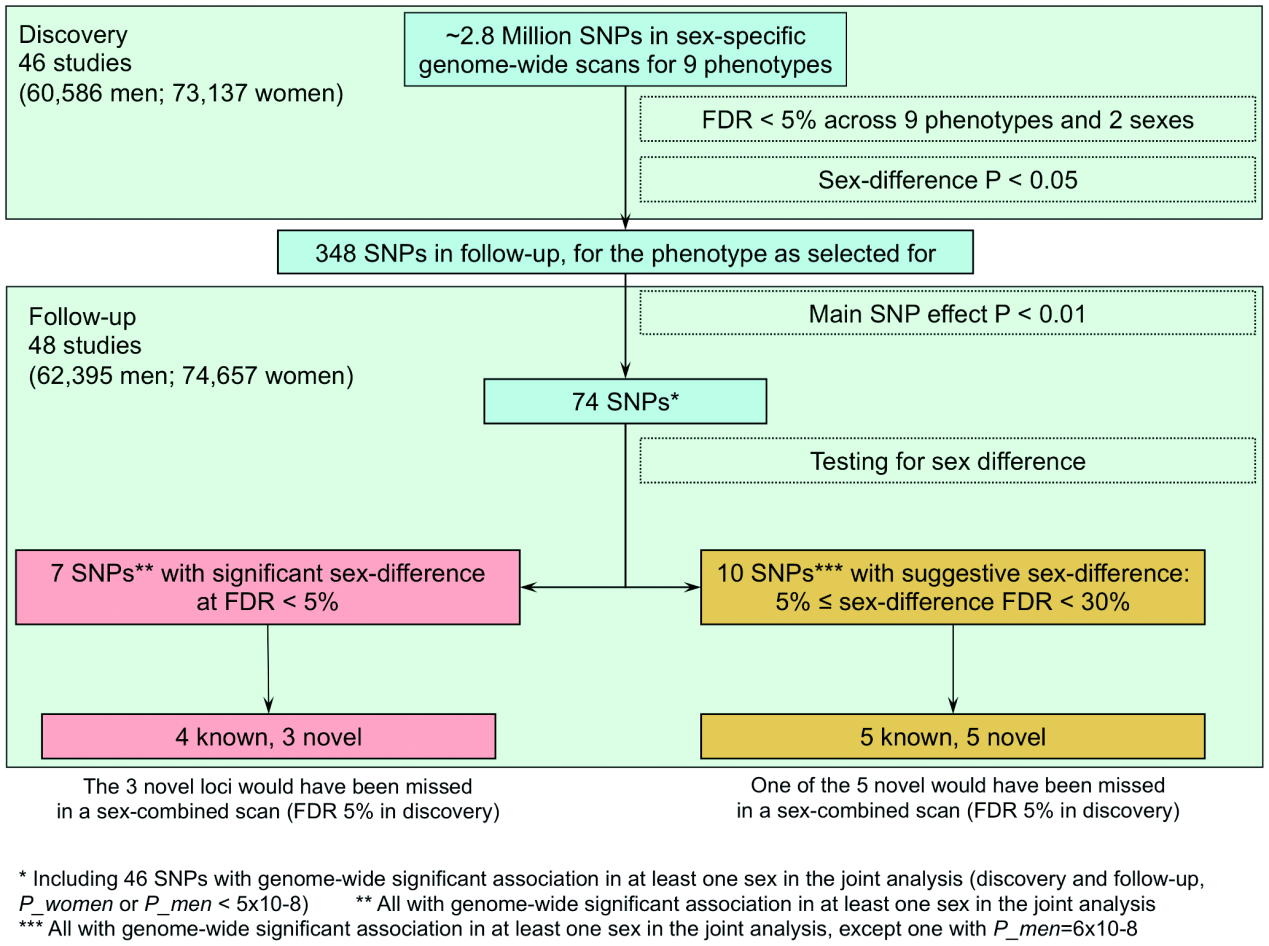
Overview of design and findings. Among the 7 identified loci, we defined those close to (<1 cM) published hits [25], [29], [31] as *near published hit*s and *novel* otherwise. Novel loci with sex-combined discovery P-value<5.8×10^−5^, which is the P-value cut-off corresponding to 5% FDR, were declared as loci that *could have been discovered also with sex-combined analysis*, and otherwise that these *would have been missed without the sex-stratified analyses*. FDR = false discovery rate.

The remaining four loci were near (<1 cM) previously established sexually dimorphic loci for WHRadjBMI (*GRB14/COBLL1*, *LYPLAL1/SLC30A10*, *VEGFA*, and *ADAMTS9*; see Table 2) [31]. The further sexually dimorphic WHRadjBMI loci previously reported in that work were included among the ten additional SNPs at 30% FDR in our data (*RSPO3*, *HOXC13*, *ITPR2-SSPN*, see Table S6), which illustrates the pay-off between our power gain from this sex-specific approach and larger sample size with the increased multiple testing burden of interrogating nine phenotypes in comparison to one phenotype in our previous work. An overview of the SNP selection and findings is given in Figure 3 and the genes surrounding the seven signals are depicted in the region plots (Figure S2).

**Table 2:** Seven identified SNPs compared to previously published loci.

SNP	CHR	POS_B36	Effect allele	Other allele	EAF^a^	Discovery sex combined P	Published Gene^b^	Published SNP	Position of published SNP	Trait as published	Previous evidence for sex difference
rs6717858	2	165,247,907	t	c	0.581	5.85E-10	*GRB14*	rs10195252	165221337	WHRADJ [31]	Yes
rs2820443	1	217,820,132	t	c	0.718	2.69E-12	*LYPLAL1; LYPLAL1*	rs11118346; rs4846567	217810342; 217817340	HEIGHT [25]; WHRADJ [31]	Yes
rs1358980	6	43,872,529	t	c	0.474	1.42E-10	*VEGFA*	rs6905288	43866851	WHRADJ [31]	Yes
rs11743303	5	55,895,709	g	a	0.208	1.34E-03	*-*	-	-	-	No
rs2371767	3	64,693,298	g	c	0.720	2.33E-06	*ADAMTS9*	rs6795735	64680405	WHRADJ [31]	Yes
rs10478424	5	118,816,919	a	t	0.784	1.58E-04	-	-	-	-	No
rs4684854	3	12,463,882	c	g	0.418	1.05E-04	-	-	-	-	No

a The Effect allele refers to a positive effect direction in the discovery stage for the trait and gender, the SNP was selected for;

b Gene near this SNP which was published previously from sex-combined analyses.

The seven SNPs with sex difference are considered to depict a known locus, if the index SNP is close to a published top SNP (<1 cM). These include four of the previously reported sexually dimorphic WHR loci (Heid et al., Nat Genet 2010).

Although identifying sex-differences was our primary goal, we note that among the 348 SNPs chosen for follow-up, 46 SNPs exhibited genome-wide significant association in either men or women in the joint analysis of discovery and follow-up data (*P-men* or *P-women*≤5×10^−8^, 27 SNPs for height, 12 for WHRadjBMI, three for weight, three for BMI, one for WCadjBMI, zero for WC, HIP, HIPadjBMI, or WHR). Detailed information regarding P-values and effect estimates of these 46 SNPs are included in Table S2.

### No opposite effect direction, but enrichment for genetic effects in women

**Figure 4:**
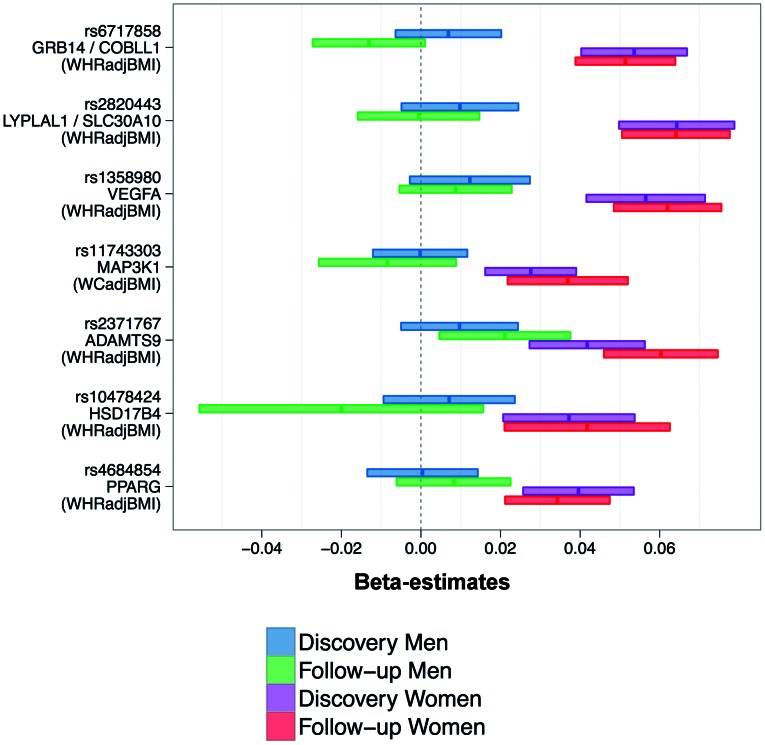
Consistently higher effect sizes for women for all seven loci. Shown are beta-estimates and 95% confidence intervals for the seven identified SNPs (also stating the phenotype for which the SNP was selected for).

When examining the sex-specific effect estimates for the seven SNPs (Figure 4), we found that effect sizes were consistent in discovery and follow-up and that none of the seven loci showed OED. Furthermore, the associations for six of the seven SNPs were observed in women only (SEE), whereas for one SNP (*ADAMTS9*) we observed CED in both sexes, but the effect was more pronounced in women than in men. The absence of loci with OED together with the observation that the sex-difference scan did not detect any sex-difference, even at 30% FDR, our data does not support the existence of genetic loci that have opposite effect in men versus women.

When comparing the effect sizes of the 46 SNPs with genome-wide significant sex-specific associations between women and men, we found again significant enrichment for larger effects in women for WHRadjBMI (Binomial test *P* = 1.1×10^−4^, Methods), but not for other phenotypes (*P* = 0.08, 0.08, 0.11, 0.16, for BMI, weight, height, or WCadjBMI, respectively). This underscores that our data does provide evidence for sexual dimorphism in the genetics, and thus biology, underlying WHRadjBMI, but not for height or BMI. This is consistent with the fact that the seven loci with confirmed sex-difference were for waist phenotypes only. Nevertheless, it should be noted that we identified suggestive sexually dimorphic genetic signals for height and BMI when applying a 30% FDR threshold (Table S6).

### Age-stratified analyses and association with other traits for the seven SNPs

Hormonal changes during menopause affect a woman's body shape and composition, generally resulting in a more android body type. Therefore, we examined whether any of the seven confirmed sexually dimorphic loci showed evidence of age-specific effects (Methods). More specifically, we performed association analyses for the seven loci stratified by age with a cut-off at 50 years (i.e. average age of onset of menopause) and by sex. None of the loci showed evidence for age-specific effect among women (or men) (*P* for difference between age groups >0.135, Table S7).

When extending the investigation of the seven SNPs from the phenotype for which the SNP was selected (six for WHRadjBMI, one for WCadjBMI) to the other anthropometric phenotypes (Tables S8A–C), we found no nominally significant association with height (joint discovery and follow-up *P-women* and *P-men* from 0.065 to 0.86), except for one SNP (rs2820443, *P-women* = 2.8×10^−6^, *P-men* = 6.0×10^−4^). Four of the seven associations showed some evidence of BMI association (*P-women* or *P-men* 3.2×10^−4^ to 6.0×10^−3^). More specifically, we found – in women only – decreased HIPadjBMI (*P-women* from 2.7×10^−27^ to 0.015) and increased WCadjBMI (*P-women* from 7.6×10^−22^ to 3.82×10^−4^) for all WHRadjBMI increasing alleles, whereas no association with HIPadjBMI (*P-women* = 0.32) was observed for the SNP selected for WCadjBMI. This underscores that the seven sexually dimorphic SNPs are primarily waist- and WHR-related.

Using data from other GWAS consortia [34]–[36], we evaluated whether the seven SNPs showed associations with other metabolic traits consistent with the observed association with WHRadjBMI or WCadjBMI and whether the similar sex-specific pattern of association was also observed (Methods). We did indeed find directionally consistent enrichment (binomial *P*<0.05) for women-specific associations (*P-women*<0.05) with lipids, fasting insulin, type 2 diabetes, and HOMA-B (binomial *P* from 1.2×10^−5^ to 5.9×10^−3^; Tables S8D–G). Remarkable was the consistent women-specific association for the index SNP near the *GRB14/COBLL1* with HDL-cholesterol, triglycerides, insulin, and type 2 diabetes (here though for a different SNP, but correlated with our index SNP, D′ = 1.0, r^2^ = 0.735) and for our SNP near *MAP3K1* with triglycerides. Three of our novel SNP findings localize near well-known loci for type 2 diabetes (*ADAMTS9*, *VEGFA*, *PPARG*), although only our SNP near *ADAMTS9* displayed a strong correlation with the published type 2 diabetes index SNP (rs4607103, r^2^ = 0.9, ∼0.001 cM), while the other two SNPs were uncorrelated with the reported type 2 diabetes SNPs (rs9472138, *VEGFA*, r^2^ = 0.008, ∼0.23 cM distant from our lead SNP; rs17036101, *PPARG*, r^2^ = 0.024, ∼0.15 cM). It should be noted that many of the studies that participated in GIANT also participated in the other consortia and given the correlation between the phenotypes, the sex-specific consistency is likely somewhat inflated. Taken together, our findings suggest common genetic influences on body fat distribution, lipids, and type 2 diabetes, particularly for women.

### Pathway analyses

In order to summarize the biological pathways that are primarily depicted by our data on sex-difference, we examined whether the genes harbored by the seven confirmed loci showed enrichment for particular pathways or other units of the molecular networks (processes, functions) using MAGENTA (Methods). We found that PPARG Signaling, post-Golgi vesicle-mediated transport and kinase- and annexin-related molecular functions showed enrichment at 5% FDR (Table S9).

### Potential functional or biological role of the seven loci

Regarding the biological role of the SNPs and genes in the proximity of the seven sex-specific SNPs, we searched literature and functional annotation data bases and catalogues (Methods). The genes inflicted in the seven regions of interest generally highlighted genes with a reported role in insulin sensitivity (*PPARG*, *VEGFA*, *ADAMTS9*, *GRB14*) and lipid-related traits (fatty liver: *LYPLAL1*; triglyceride concentrations: *MAP3K1*, HDL-C: *GRB14*). Among the index SNPs or their proxies (pairwise correlation, r^2^>0.8) in the immediate region (49 SNPs altogether), we found one SNP (rs10478424; r^2^ = 1 with lead SNP at *HSD17B4*) that was a predicted transcription factor binding site (TFBS). Interestingly, one of the transcription factors predicted to bind at this TFBS is *PPARG*, which itself is located near one of our other association signals. None of the other 48 SNPs tagged any known copy-number variant, was a non-synonymous coding variant, or was present in any of the other predicted functional classes. When extending this search to SNPs that were more moderately correlated (r^2^>0.5) with the lead index SNPs (146 SNPs altogether), these included several proxies of rs2820443 (near *LYPLAL1*/*SLC30A10*) that annotated as TFBSs as well as proxies of rs10478424 that disrupt predicted microRNA binding sites. These findings may indicate potential involvement in the regulation of gene transcription near those loci.

A specific description of potentially functionally elements in the association regions as indicated by UCSC and Ensembl genome browsers and more details from the literature and functional annotation data base searches can be found in the Text S1.

### Effect of the seven sexually dimorphic loci on expression in relevant tissues

To localize the potentially causal gene at each locus, we examined the evidence for sex specific *cis* expression quantitative trait loci (eQTL) for genes near the seven identified SNPs in different types of human subcutaneous adipose tissue, lymphocytes, and whole blood (Methods). Although there was evidence for gene expression association of two (*GRB14*, *ADAMTS9*, Table S10) of the seven SNPs (SNP highly correlated with the peak SNP of the transcript, r^2^>0.8, the association of the peak SNP with the transcript expression vanished when adjusted for our SNP), the associations were not sex-specific (*P-diff*>0.05).

We also examined whether genes harbored by the seven sex-specific loci showed sex-specific expression in various tissues of mouse models using real-time PCR expression data (brown fat, inguinal and gonadal fat, and liver) and Illumina expression data (liver, inguinal and gonadal fat; Methods). We found significantly (at a significance level of 0.05/19 = 0.003) lower expression of *GRB14* in brown fat of female mice (*P-diff* = 0.001); due to the role of brown fat in triglyceride catabolism [37], this is in-line with the previously described sexually dimorphic association of this SNP with HDL-C and triglycerides. For female compared to male mice, we found significantly lower expression of *VEGFA* (*P-diff* = 1×10^−5^) in inguinal fat, whereas in liver, we found higher expression for three genes (*LYPLAL1*, *PPARG*, *MKRN1*; *P-diff* from 0.002 to 0.003) with the latter two genes being located in the *PPARG* locus (Table S11).

## Discussion

In our genome-wide search for sexually dimorphic associations including over 270,000 individuals from 94 studies from the GIANT consortium, we found evidence for seven loci with significant sex-difference including three novel anthropometric trait loci (near *MAP3K1*, *HSD17B4*, *PPARG*). Importantly, for all seven loci, the associations were observed for waist phenotypes with more prominent effects in women. These findings are consistent with our previous reports for sex-differences in the genetics of WHR [30], [31].

The waist phenotypes used in this study are well established proxy measures of body fat distribution. Women have more subcutaneous body fat, which is part of the skin, that is preferentially deposited at the hips and thighs whereas men have more visceral fat, which is fat in and around the inner organs and accumulates particularly around the waist [38]–[40]. It is well known that hormonal levels are associated with differences in fat distribution in men and women, distinctions that emerge early in childhood and subsequently amplify during puberty [41], [42]. Moreover, fat distribution in women changes as estrogen levels drop during menopause, leading to a more android shape, with greater visceral fat accumulation [43]. Subcutaneous and visceral fat has distinct morphological and functional properties that account in part for clinically relevant sex differences in a variety of metabolic phenotypes [40], [44].

Our findings of sex-specific genetic effects on waist-hip-ratio as a measure of fat distribution are consistent with a study of families in whom *MC4R* mutations segregate that demonstrated larger effects on obesity in female compared to male mutation carriers [45]. In animal models, microarray experiments have consistently demonstrated that adipose tissue mass, function, and distribution is regulated by networks of sexually dimorphic genes which are likely regulated by sex hormones [46]. In addition, sex-differences in mRNA and miRNA expression in abdominal and gluteal adipose tissue have been noted in humans [47], [48]. Lastly, animal studies demonstrate that exposure to sex hormones early in development is associated with long term, lifelong changes in adipose tissue distribution and function [49], [50]. Taken together, sex-differences in body shape appear to be determined by a complex interplay of genetic and hormonal factors.

Our data did not provide statistically significant evidence for sex-differences in the genetics of BMI and height. This is in-line with previous reports in a large twin heritability study [51], which shows differential heritability between sexes for waist-hip-ratio, but not for BMI or height. Interestingly, phenotypic differences in BMI between men and women are consistently smaller than those for waist-related traits and perhaps sex differences in BMI genetics are more subtle. This was not very surprising as our expectations for sex-differences were highest for waist-hip-ratio, given the previous report on sex-differences in waist-hip-ratio genetics, the strong link of the phenotype to body fat distribution, the change of body fat distribution by hormones, and the sex-specificity of fat distribution.

The lack of signals with opposite effect direction for men and women in our data is particularly intriguing. Given that no systematic search for sexually dimorphic associations with anthropometric traits has been done before in a large enough genome-wide effort, it was not really clear whether OED signals would exist. While our systematic search scanning the P-values testing for sex-difference between the sex-specific effect estimates was specifically designed to detect OED associations, we did not detect any. This is in-line with the prior believe that genetic variants do not affect anthropometry in one direction in men and in the opposite direction in women.

The seven loci shed new light on regions containing genes with a reported role for type 2 diabetes (*PPARG*, *ADAMTS9*, *VEGFA*), lipids (*GRB14*, *MAP3K1*) and hormone metabolism (*HSD17B4*). Particularly intriguing due to its relevance for type 2 diabetes and therapy is the *PPARG*, which showed association with WHR in women, but not in men. This is in-line with small candidate gene-by-environment interaction studies e.g. of saturated fat intake with *PPARG* variants for obesity-related traits [52], including differential effects by sex [53]. Although the index SNP is independent of the polymorphisms previously reported for type 2 diabetes [54]–[56], *PPARG* is a particularly interesting candidate as its encoded protein, PPAR*γ*, is a nuclear hormone receptor that serves as a master regulator of adipocyte-specific genes contributing to adipocyte differentiation, susceptibility to obesity, and insulin sensitivity [33]. PPARg-agonists are used to treat type 2 diabetes by redistributing adipose tissue from abdominal visceral to subcutaneous compartment, which is thought to be preferable and improve insulin sensitivity. Interestingly, sex-differences in pioglitazone response have been described for nondiabetic overweight persons [57].

Furthermore, growth factor receptor-bound protein 14 (GRB14) binds to insulin receptors and inhibits their catalytic activity. GRB14 is a prerequisite for the development of insulin-sensitizing molecules to pathological states as obesity and type 2 diabetes [58]. Our current and previously reported findings [31], [34] suggest that the rs6717858 near *GRB14* has a female-specific effect on insulin as well as on central obesity and lipids. Additionally, we highlight a region including the mitogen-activated protein kinase kinase kinase 1 (near *MAP3K1*), a locus known to be associated with triglyceride levels in sex-combined analyses [59], whereas we found this locus to be associated with WHR in women only. The *MAP3K1* plays a pivotal role in a network of phosphorylating enzymes integrating cellular responses to a number of mitogenic and metabolic stimuli, including insulin and many growth factors [60]. Interestingly, mutations in *MAP3K1* have recently been demonstrated to result in a 46XY disorder of sex development with varying manifestations of gonadal dysgenesis [61]. Thus this gene is implicated in normal sex development, which may be related to our observed sexually dimorphic genetic effect. Finally, the locus near *HSD17B4* is of particular interest, because HSD17B4 is a multifunctional peroxisomal enzyme involved in steroid metabolism and fatty acid oxidation [62], [63]. It converts the more active hormone, estradiol, to the less active estrone. Sex differences in *HSD17B4* expression with estradiol supplementation have been noted in zebra fish [64]. In addition, the position of our lead marker at a predicted transcription factor binding site located just upstream of a putative protein coding splice variant of *HSD17B4* indicates a potential function for the association signal observed at this locus and may warrant additional follow-up work. Interestingly, among the genes that bind to this transcription factor binding site is PPARG.

A major strength of our study is that we were uniquely positioned to perform the analyses described, taking advantage of the highly efficient collaborative environment of the many study partners within the GIANT consortium, which allowed us to conduct the largest possible sex-difference GWAS for anthropometric phenotypes ever reported. As a consequence, we were well-powered (about 80% power) to detect sex-sensitive genetic effects of the same magnitude as those observed previously for WHR or to detect genetic effects as previously observed for height, but assuming these to appear only in one sex [25], [31]. Nevertheless, our statistical power to detect subtle sex differences in genetic effects was limited. Notably, we used a conservative approach to avoid false positive claims: (i) we used ranks instead of the absolute phenotypic values of anthropometric traits in order to avoid artefacts due to outliers, (ii) we applied double genomic control correction in order to avoid any artefact from possible population stratification, and (iii) we established sex-difference using our follow-up as opposed to the combined discovery+follow-up data sets to avoid overestimated sex-differences through winner's curse. It is a further strength that we were able to show associations of our sex-specific anthropometric trait signals also with other metabolic traits such as lipids, glucose and type 2 diabetes; however, we need to note the limitation that these associations were not adjusted for the anthropometric traits, so that some of the observed metabolic trait associations were expected due to the correlation of anthropometric traits with lipids, glucose and type 2 diabetes.

Overall, we found women-specific SNP effects for anthropometric traits, particularly for waist-related phenotypes. Our study findings lend support to distinct genetic effects on body shape by sex and argue for the importance of further integrative studies of sex differences of body shape. While the actual underlying genes and their mechanisms of action remain elusive, we hypothesize that such differences are hormonally regulated. Moreover, because body fat has a prominent endocrinological function and body fat distribution has a critical relevance for many metabolic pathways, understanding these differences could help improve our understanding of metabolic disease processes. Particularly the established sex-difference for the SNP near the therapy-relevant *PPARG* could impact treatment options. In the era of personalized medicine, which attempts to tailor treatment to fit the individual, a better differentiation between men and women in research and patient treatment could be an important start.

### Summary and conclusion

While our data underscores a lack of genetic association in opposing direction in men versus women, we have highlighted female-specific effects in waist phenotypes. Our investigation underscores the importance of considering sex-differences when interrogating the genetic architecture of anthropometric traits. For those traits with strong a priori evidence for sex differences, the routine analysis of sex-specific genome-wide analyses may allow for numerous options for meta-analysis including a sex-combined scan optimally powered to detect the general association as well as sex-specific scans when searching for sexually dimorphic signals. Although our study focused on sex-differences for anthropometric traits, sex differences in genetic effects likely exist for other traits and diseases and should be taken into consideration in future genetic as well as translational studies.

## Methods

### Anthropometric phenotypes

The anthropometry of men and women differ in various aspects: Average height, waist circumference, and WHR, is higher for men than for women, whereas average BMI is similar. Variability for all phenotypes is similar for men and women, which can be seen on the example of the KORA study (Table S12) and specifically for WHR adjusted for BMI for all studies (Figure S3).

The anthropometric traits examined are height (cm), weight (kg), body mass index (BMI, kg/m^2^) computed as weight divided by meter of height squared, waist circumference (WC, cm), hip circumference (HIP, cm), and waist-hip-ratio (WHR). The last three traits were analyzed without and with adjustment for BMI, yielding nine phenotypes in total (height, weight, BMI, WC, HIP, WHR, WCadjBMI, HIPadjBMI, WHRadjBMI). For the further analyses, the traits were all transformed at the study-level by calculating age-adjusted residuals (including age and age^2^ into the regression model for trait creation) for men and women separately and adding BMI into the adjustment as indicated above; then – for all traits except height – the values were ranked and an inverse normal transformation was applied, whereas a z-score transformation was performed for height.

### Study-specific analyses for discovery and follow-up

For discovery stage, we included 46 studies (up to 60,586 men, 73,137 women) on height, weight and BMI, 34 studies (up to 36,231 men, 45,192 women) on WC, 33 studies (up to 34,942 men, 43,316 women) on HIP and 32 studies (up to 34,629 men, 42,969 women) on WHR. Each study was genotyped using either Affymetrix or Illumina arrays. To enable meta-analyses across different SNP panels, each group performed genotype imputation using HapMap II CEU (build 21 or 22) via MACH [65], IMPUTE [66] or BimBam [67]. Details are given in Tables S1, S3, S4, S5 and Text S2.

For follow-up, we included (i) 30 studies (up to 42,055 men, 32,785 women) for height, weight and BMI and 27 studies (up to 36,671 men, 28,326 women) for WC, WCadjBMI, HIP, HIPadjBMI, WHR and WHRadjBMI that were genotyped using the custom iSELECT Metabochip array containing ∼195K SNPs designed to support large-scale follow-up of putative associations with metabolic and cardiovascular traits, and (ii) 18 studies (20,340 men, 41,872 women) for height, weight, and BMI and 14 studies (11,225 men, 32,610 women) for WC, WCadjBMI, HIP, HIPadjBMI, WHR and WHRadjBMI genotyped using genome-wide SNP chips with subsequent imputation for *in silico* follow up.

In each study, association was tested separately for men and women. The additive genetic effect for each SNP on each phenotype was estimated using a normal linear regression model using MACH2QTL [68], SNPTEST [66], ProbABEL [69], GenABEL [70], Merlin [71], or PLINK [72]. For studies with a case-control design, cases and controls were analyzed separately. Study-specific information was described previously [25], [29], [31] for discovery studies and in Tables S1, S3, S4, S5 for follow-up studies.

All involved studies were conducted according to the principles expressed in the Declaration of Helsinki. The studies were approved by the local Review Boards and all study participants provided written informed consent for the collection of samples and subsequent analysis.

### Sex-specific discovery meta-analysis

All discovery study-specific files were processed in the meta-analysis centers through a standardized cleaning script that included checks of allele frequencies, compliance with Hapmap alleles, file completeness, number of markers, and ranges of test-statistics. We excluded monomorphic SNPs, SNPs with MAF*N≤3 (minor allele frequency multiplied by sample size) and SNPs with poor imputation quality, i.e. r2_hat <0.3 in MACH; observed/expected dosage variance <0.3 in BIMBAM; proper_info <0.4 in IMPUTE; information <0.8 in PLINK [65], [66], [72], [73].

Sex-specific standard errors and P-values from each participating study were genomic-control (GC) corrected [74] using the lambda factors as published [25], [29], [31], then beta-estimates were meta-analyzed using the inverse-variance weighted fixed effect model as implemented in METAL [75]. A sensitivity analysis using the sample-size weighted Z-score meta-analysis approach yielded the same results; only fixed effect model results are shown. The 2,971,914 SNPs in each of 18 analyses (nine phenotypes in two sexes) reduced to 2,846,694 SNPs with available chromosome and position annotation in dbSNP. The genetic position (cM) was extracted from HapMap release 22 (http://hapmap.ncbi.nlm.nih.gov/downloads/recombination/2008-03_rel22_B36/rates/) or - if unavailable - approximated by the inverse-distance weighted average of the genetic positions of the nearest HapMap SNPs (release 22) on each side.

### SNP selection strategy

We conducted two types of genome-wide searches in the discovery stage: (a) In the *sex-specific scan*, we computed sex-specific association P-values for each SNP, concatenated these for all nine phenotypes totaling 50,586,560 P-values (i.e. 2 sexes×9 phenotypes×2.85 Million SNPs), and selected 20,215 SNPs at 5% FDR [32]. Pruning this list to independent SNPs (starting with the 20,215 SNPs sorted by increasing P-value and deleting SNPs within 0.2 cM of any of the SNPs above) yielded 619 independent SNPs. For each SNP and for the phenotype that the SNP was selected for, we also computed P-values (*P-diff*) testing for difference between the meta-analyzed men-specific and women-specific beta-estimates 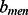, 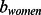 with corresponding standard errors 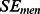 and 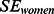 using the t statistic
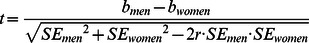
The correlation 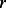 between 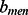 and 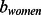, computed as the Spearman rank correlation coefficient across all SNPs for each phenotype, ranged from 0.04 to 0.18 across phenotypes. From these 619 SNPs, we selected the 348 SNPs with nominally significant sex-difference (*P-diff*<0.05) to ensure some level of sex-difference in the discovery. Whether the sex-difference was significant was then evaluated in the follow-up stage (see below). (b) In the *sex-difference scan*, *w*e computed *P-diff* for each of the ∼2.85 Million SNPs and each of the nine phenotypes and concatenated the totaling 25,293,280 P-values. We had planned to select SNPs for follow-up at an FDR of 5% across all SNPs and phenotypes, but there were none. Power considerations are provided in the Text S1 and Figure S4A.

### Follow-up and joint meta-analysis, establishing sex-difference

Study-specific follow-up data were quality-controlled in a similar manner as discovery data with increased attention towards strand-issues. We conducted sex-specific follow-up meta-analyses using the same statistical models as for the discovery. We combined (i) *in silico* studies and (ii) metabochip studies, and then combined results of (i) and (ii) implying a double genomic control correction (Text S1). Additionally, we conducted a joint meta-analysis combining the sex-specific association results of discovery and follow-up.

For SNPs selected for their small P-values of sex-specific association, the sex-difference estimates and corresponding P-values in the same data set would be inflated (see Figure S5) as the two tests are not independent. We therefore derived sex-difference estimates and corresponding P-values in the follow-up data alone.

As none of our selected SNPs stemmed from the scan targeted for OED signals (i.e. the sex-difference scan) or showed any evidence of OED in the discovery, we targeted our follow-up analysis for CED or SSE signals. We filtered for a main effect (*P* for both sexes combined <0.01) prior to testing for sex-difference (*P-diff*, as described above), since this increased the power to detect SSE and CED signals (Figure S4B, Text S1), while this filter did not introduce a bias such as a sex-stratified association filter would. SNPs with a *P-diff* in the follow-up at 5% FDR were considered as SNPs with *significant sex difference*.

### Establishing genome-wide significance of anthropometric trait association and enrichment for female or male genetic effects

We considered a joint (discovery and follow-up combined) association *P-men* or *P-women*<5×10^−8^ as *genome-wide significant*. For each phenotype, we tested whether there were more male-specific or more female-specific associations among the associations with established genome-wide significance in at least one sex compared to the expected binomial distribution.

### Age-stratified sex-specific meta-analysis and association with metabolic traits

For the identified signals with sex-difference, study partners of the discovery and the *in silico* follow-up re-analyzed their data stratified by sex and age group (≥50 years, <50 years) using the same models as described above. Age difference was tested within each sex using the same t statistic as applied for the sex-difference testing.

Sex-specific associations of the identified signals with metabolic traits were derived requesting a sex-stratified re-analysis from the Global Lipids Genetics Consortium (GLGC, Triglycerides, HDL-, LDL-, and Total cholesterol) [34], the Meta-Analyses of Glucose and Insulin-related traits Consortium (MAGIC, fasting insulin, fasting glucose, HOMA-B, HOMA-IR) [35], [76]; and, the DIAGRAM consortium (type 2 diabetes) [36]. We tested the overall number of SNPs with consistent nominally significant association for the sex that the SNP was selected for (*P_women* or *P_men* <0.05) compared to a binomial draw with an event rate of 0.05. It needs to be noted that this test does not account for the correlation between the traits nor for the fact that the consortia involve an overlap of studies.

### Pathway analyses

In order to explore whether certain pathways are enriched among the genes depicted by loci with evidence for sex-difference, we applied MAGENTA [77]. Briefly, MAGENTA calculates gene-specific scores (for ∼18,000 genes) by combining the p-values (here: the sex-difference P-values from our discovery stage for a specific anthropometric phenotype) of SNPs in and around the genes (40 kb down-, 100 kb upstream). The genetic score is corrected for potential confounders, such as gene size, number of independent SNPs, LD pattern, length in genetic distance, and number of recombination hotspots. These scores are ranked and the genes within the top 5% of these scores are tested for enrichment in certain pathways (separately for each phenotype) as given by different databases (GO: http://www.geneontology.org/, KEGG: http://www.genome.jp/kegg/, Ingenuity: http://www.ingenuity.com/products/pathways_analysis.html, and PANTHER: http://www.pantherdb.org/). MAGENTA determines whether the genes among the 5% top scores link to certain pathways more often than expected by chance. FDR is controlled at 5% via 10,000 permutations (using a random set of genes with the same number of genes as those observed).

### Search for biological and functional knowledge of the seven association regions

For the seven confirmed sex-difference loci (defined as the regions depicted by SNPs within 1.0 cM of the respective lead SNP showing a certain level of association, P≤100 * P_leadSNP_), we searched several catalogues and data bases to depict potential biologically relevant links or functional entities. We extended the regions of interest to +−500 kB around the lead SNP if the regions were very small and no gene was inflicted (as for the PPARG, VEGFA, MAP3K1 loci).

First, we performed an automated search for reported genes or variants in our regions in PubMed (http://www.pubmed.com) and OMIM (http://www.ncbi.nlm.nih.gov/omim) using Snipper (http://csg.sph.umich.edu/boehnke/snipper) or manually inspected UCSC (PMID: 22086951) and Ensembl (PMID: 21045057) genome browsers as well as the NHGRI GWAS catalog [78], [79]. Second, we explored whether SNPs known to provide reliable tags for Copy-Number-Variations (CNVs) in European-descent samples (combining four catalogues including 60167 CNV-tagging SNPs as described previously [31]) correlated with our lead SNPs. Third, we performed several online database searches to establish whether known variants within 500 kb of each lead SNP, that are correlated (r^2^>0.8 or 0.5) with our lead SNPs (using SNAP Proxy search [80]), might have putative or predicted function. (i) We searched the SIFT database [81] to determine whether any of these SNPs was predicted to affect protein function. (ii) We used SNPinfo [82] to investigate predicted and putative function in several functional classes, including splicing regulation, stop codons, polyphen predictions, SNPs3D predictions, transcription factor binding site (TFBS) prediction, and miRNA binding site prediction.

### Expression QTL analyses in human and mouse tissue

We examined transcript expression of genes near each of the seven identified SNP. For human eQTL, we explored four different tissues (subcutaneous adipose tissue, whole blood, and lymphoblastoid cells; details on methodology and tissue samples in the Text S1). We computed sex-specific association including conditional analyses and r^2^ measures to identify *cis* eQTL signals that were likely to be coincident with the anthropometric trait signal. For mouse eQTL, we had four types of tissues (inguinal fat, gonadal fat, liver, brown fat) with expression derived by real-time RT-PCR as well as three types of tissue (liver, inguinal fat, gonadal fat) with Illumina assays (details in Text S1). The sex-specific association and a test for sex-differences were computed.

## Supporting Information

Figure S1 Sensitivity Analysis excluding patient groups shows consistent results. Shown are sex-specific beta-estimates of the seven identified SNPs in the follow-up data without (original analysis) and with exclusion of patient groups. It can be seen that the results are robust and patient groups do not trigger the observed sex-differences.
(TIF)

Figure S2 Region plot of the 7 identified loci showing the (a) association P-value in women, (b) association P-value in men, and (c) the sex difference P-value.
(PDF)

Figure S3 Distribution of waist-to-hip ratio adjusted for BMI. Shown are the 5^th^, 25^th^, 50^th^ (median), 75^th^, and 95^th^ percentiles of the residuals of waist-hip-ratio (before inverse normal transform) adjusted for BMI (therefore zero mean) for each contributing study. It can be seen that the variability of the phenotype is symmetric and to a similar extent in men and women.
(TIFF)

Figure S4 Power comparison. (A) Discovery: Shown is the power for selecting a sex-sensitive SNP (42969 women, 34629 men; assuming a signal as *PPARG* in women, MAF = 0.42, R^2^_women_ = 0.00057, various effects for men) into follow-up at α = 2×10^−5^ for the sex-specific (orange) scan, the sex-difference scan (magenta), or the sex-combined scan (black). (B) Follow-up: Power (60936 women, 47896 men; assuming a signal such as *PPARG* as above in women, various effects for men) to establish sex-difference among the 348 SNPs in the follow-up by (i) testing all 348 SNPs for sex-difference (no prior filter for a main effect; blue) at 5% FDR (corresponding to a *P-diff* of 9.9×10^−4^; blue), or by (ii) testing first for a main effect (P-value combined for men and women <0.01) and then testing the remaining 74 SNPs for sex-difference at 5% FDR (here corresponding to a *P-diff* of 4.2×10^−3^; red).
(TIF)

Figure S5 Inflated P-values of the sex-difference test due to the selection on sex-specific association. We have simulated 1 Million SNPs under the null hypothesis of no sex-difference (and no association), selected 348 SNPs with the most extreme sex-specific association, and plotted the observed P-values of the sex-difference test compared to the expected. It can be seen that the observed sex-difference P-values are inflated (i.e. do not lie on the identity line), which indicates that the sex-difference test is not independent from the sex-specific association selection.
(TIFF)

Table S1 List of studies including sample sizes.
(XLS)

Table S2 Association results of the 348 loci put forward to follow-up, showing the results from 46 discovery studies including 60,556 men and 73,133 women and 48 follow-up studies including 62,397 men and 74,651 women. These include 46 SNPs with genome-wide significant association in men or women in the joint analysis of discovery and follow-up studies.
(XLS)

Table S3 Study-specific designs and references. (follow-up stage only).
(XLS)

Table S4 Study-specific methods. (follow-up stage only) (A) insilico studies, (B) metabochip studies.
(XLS)

Table S5 Study-specific descriptives. (follow-up stage only).
(XLS)

Table S6 Further ten SNP show sex-difference at 30% FDR. Shown are the SNPs with sex-difference in the follow-up according to 30% FDR additional to those shown in Table 1 **.** These include the further three loci previously reported for sexually dimorph waist-hip ratio associations, *HOXC13*, *ITPR2/SSPN*, and *RPO3/C6orf173* [31].
(XLS)

Table S7 No difference in any of the 7 associations between age groups. Age-stratified analyses results were conducted from 59 discovery and *in silico* follow-up studies for the seven identified SNPs.
(XLS)

Table S8 Sex-specific association of the 7 SNPs with other traits.(A) BMI, height and weight: From the joint discovery and follow-up including 122,907 men and 147,746 women for height, 97,482 and 97,062 for weight, and 120,975 and 142,332 for BMI. It should be noted that results in Table 1 and Table S7 are only for the phenotype and sex for which the SNP was selected. (B) hip circumference (HIP), waist circumference (WC), waist-hip-ratio (WHR): Including 75,102 men and 96,383 women for hip, 86,196 and 108,303 for waist circumference, and 76,838 and 98,747 for waist-hip-ratio. (C) hip circumference adjusted for BMI (HIPadjBMI), waist circumference adjusted for BMI (WCadjBMI), waist-hip-ratio adjusted for BMI (WHRadjBMI). Including 74,949 men and 96,353 women for HIPadjBMI, 86,036 and 108,052 for WCadjBMI, and 76,625 and 98,352 for WHRadjBMI. (D) lipid traits: From the Global Lipids Consortium (Teslovich et al., 2010) including 39,104 men and 64,235 women. (E) glycaemic traits: From the MAGIC consortium (Prokopenko et al., 2009) including 54,046 men and 60,450 women. (F) type 2 diabetes: From the DIAGRAM consortium (Zeggini et al., 2008) including 18,786 men (4,451 cases and 14,335 controls) and 28,332 women (3,680 cases and 24,652 controls). (G) Summary of enrichment statistics: for sex-specific association of the 7 SNPs with the metabolic traits.
(XLS)

Table S9 Pathway analysis reveals enrichment of PPAR signaling among genes with evidence for sex-difference. Shown are P-values for phenotype-specific enrichment using MAGENTA to determine whether certain pathways/biological processes/molecular functions are enriched among genes harboring SNPs with evidence for sex-difference. Only categories with false discovery rate (FDR)<10% are reported in the table and those with FDR<5% are considered statistically significant (bold). In short, for a certain phenotype in the discovery data, MAGENTA assigns a gene-specific score based on the sex-difference P-values of SNPs in or nearby (40 kb down-, 100 kb upstream) the respective gene. The score is calculated for each o the ∼18 000 annotated genes and corrected for gene-size, number of independent SNPs, LD pattern, length in genetic distance, and number of recombination hotspots. Then MAGENTA determines whether the genes with a score in the top 5% (∼900 genes for each phenotype) overlap with certain pathways more than expected by chance (i.e. as compared to a random gene set including the same number of genes).
(XLS)

Table S10 Associations with cis gene expression (eQTL) in human subcutaneous adipose tissue (SAT) and lymphocytes. Transcripts of genes near each of the identified seven SNPs were examined in four differenet data sets (DeCode, MoIOBB, childhood asthma study, HapMap, details in Text S1). Sex-specific associations with the transcript are shown for the identified SNP with and without conditioning on the most significant SNP for that transcript (peak SNP) and also for the peak SNP with and without conditioning on the identified SNP, if FDR<5% (DeCode) or FDR<1% (MolOBB, childhood asthma study, HapMap) in one sex. For *GRB14* and *ADAMTS9*, our identified SNP was highly correlated (r^2^>0.8) with the peak transcript SNP in men. No SNP showed nominally significant sex difference (*P_diff_*>0.05).
(XLS)

Table S11 Gene expression in mice. Shown are expression levels in female and male mice, if expression was nominally significant in at least on sex, and the P-value testing for sex difference (*P-diff*<0.05) from the mouse experiments in three different center: (i) 21 male and 21 female mice with Illumina array for inguinal or gonadal fat (Houston, H), (ii) 139 male and 133 female mice with Illumina array for liver (Oxford, O), (iii) 7 male and 7 female mice with PCR analysis for brown fat, inguinal or gonadal fat, and liver (Regensburg, R). Examined genes are listed in Table S13.
(XLS)

Table S12 Sex-specific phenotype description. Shown are mean and standard deviation (std.) of the investigated traits in a German general population study (KORA-S3 and KORA-S4). Age range is 25–75 years of age with mean of 53.6 for men and 52.9 for women. All phenotypes are adjusted for age and age^2^ as in the genetic association analyses.
(XLS)

Table S13 Genes included in mouse eQTL investigations. (A) Regensburg mouse eQTL. Also shown are primers used for analysis of gene expression. (B) Oxford and Houston mouse eQTL. We explored all genes containing SNPs with at least some level of association in the initial discovery (*P_men* or *P_women* smaller than hundred times the index SNP P-value), but at least 3 genes and at maximum all genes within ±1 cM of the index SNP (annotation using genome browser Ensembl build 54).
(XLS)

Text S1 Supplementary note.
(PDF)

Text S2 Extended acknowledgments.
(PDF)
