# Supplementary figures and images for: Sex-stratified Genome-wide Association Studies Including 270,000 Individuals Show Sexual Dimorphism in Genetic Loci for Anthropometric Traits
Source: PLoS Genet. 2013 Jun 6;9(6):e1003500. doi: 10.1371/journal.pgen.1003500 (PMC3674993; doi:10.1371/journal.pgen.1003500)

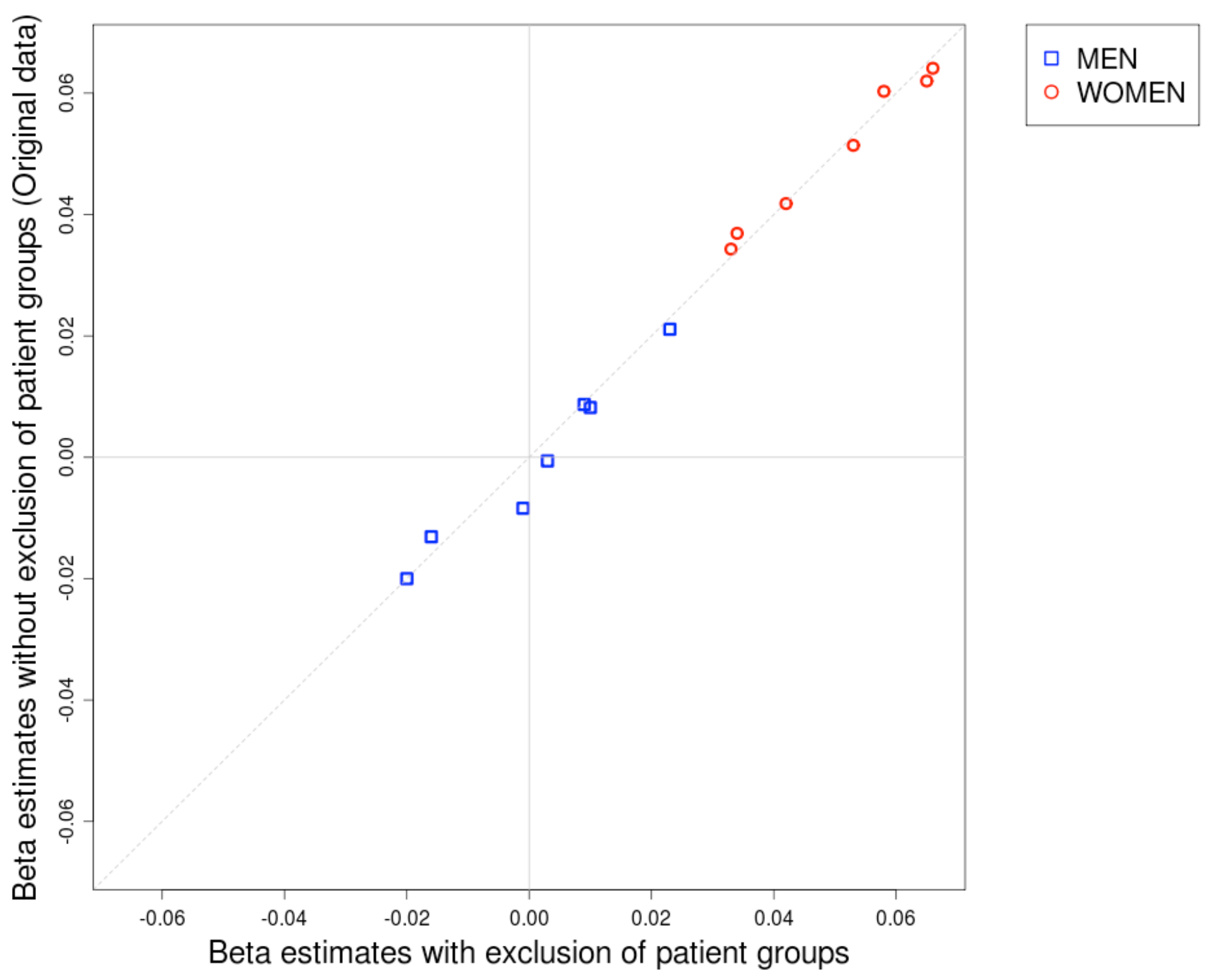

Supplement: Figure S1 — Sensitivity Analysis excluding patient groups shows consistent results. Shown are sex-specific beta-estimates of the seven identified SNPs in the follow-up data without (original analysis) and with exclusion of patient groups. It can be seen that the results are robust and patient groups do not trigger the observed sex-differences. (TIF) [file pgen.1003500.s001.tif]

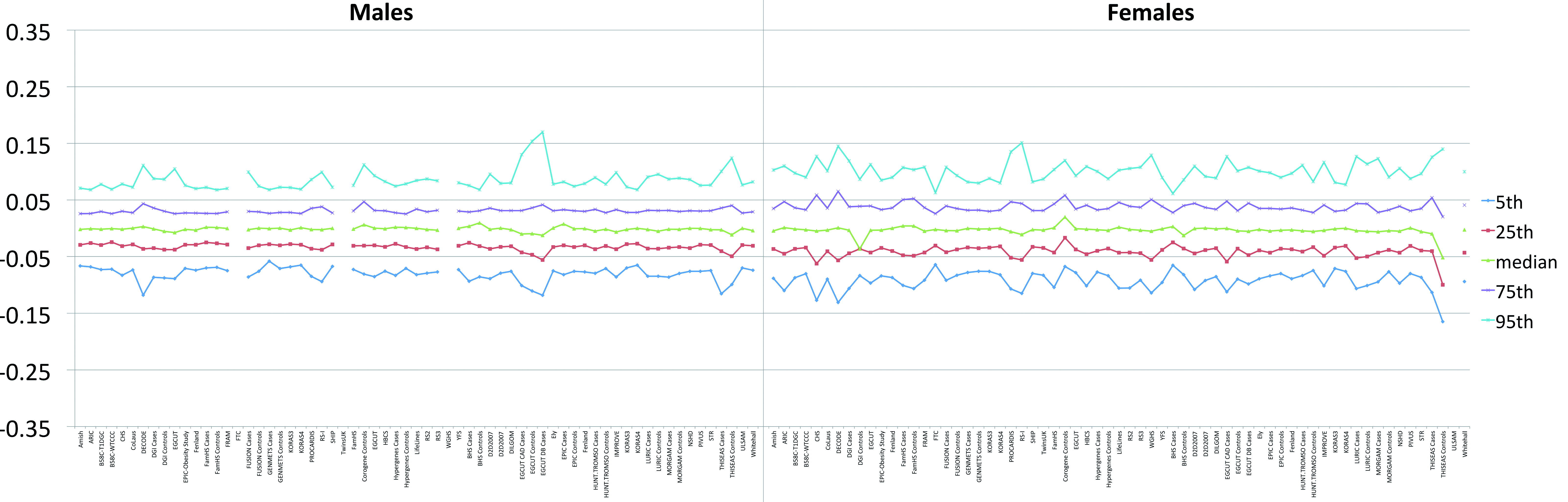

Supplement: Figure S3 — Distribution of waist-to-hip ratio adjusted for BMI. Shown are the 5th, 25th, 50th (median), 75th, and 95th percentiles of the residuals of waist-hip-ratio (before inverse normal transform) adjusted for BMI (therefore zero mean) for each contributing study. It can be seen that the variability of the phenotype is symmetric and to a similar extent in men and women. (TIFF) [file pgen.1003500.s003.tiff]

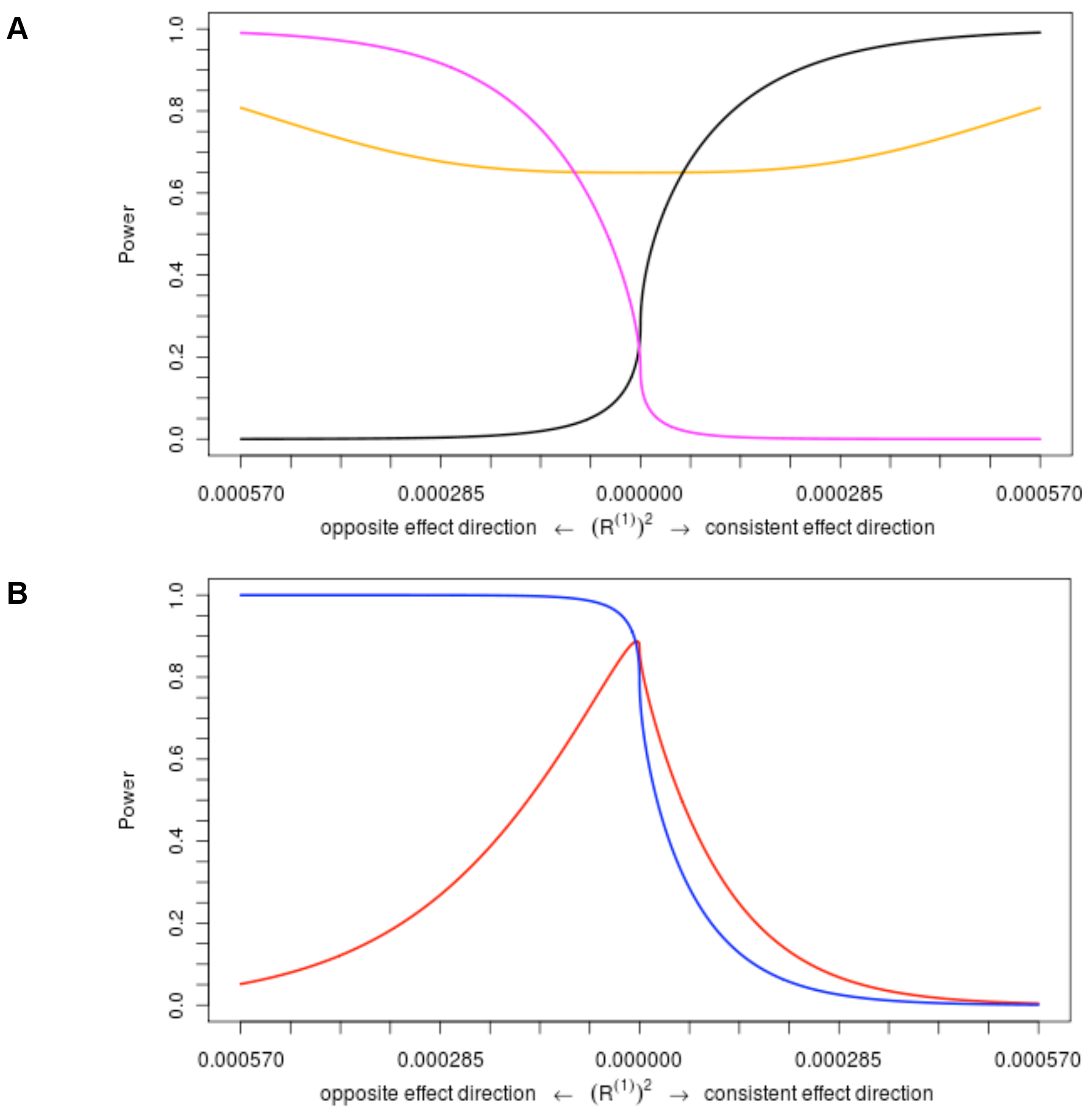

Supplement: Figure S4 — Power comparison. (A) Discovery: Shown is the power for selecting a sex-sensitive SNP (42969 women, 34629 men; assuming a signal as PPARG in women, MAF = 0.42, R2 women = 0.00057, various effects for men) into follow-up at α = 2×10^−5^ for the sex-specific (orange) scan, the sex-difference scan (magenta), or the sex-combined scan (black). (B) Follow-up: Power (60936 women, 47896 men; assuming a signal such as PPARG as above in women, various effects for men) to establish sex-difference among the 348 SNPs in the follow-up by (i) testing all 348 SNPs for sex-difference (no prior filter for a main effect; blue) at 5% FDR (corresponding to a P-diff of 9.9×10^−4^; blue), or by (ii) testing first for a main effect (P-value combined for men and women <0.01) and then testing the remaining 74 SNPs for sex-difference at 5% FDR (here corresponding to a P-diff of 4.2×10^−3^; red). (TIF) [file pgen.1003500.s004.tif]

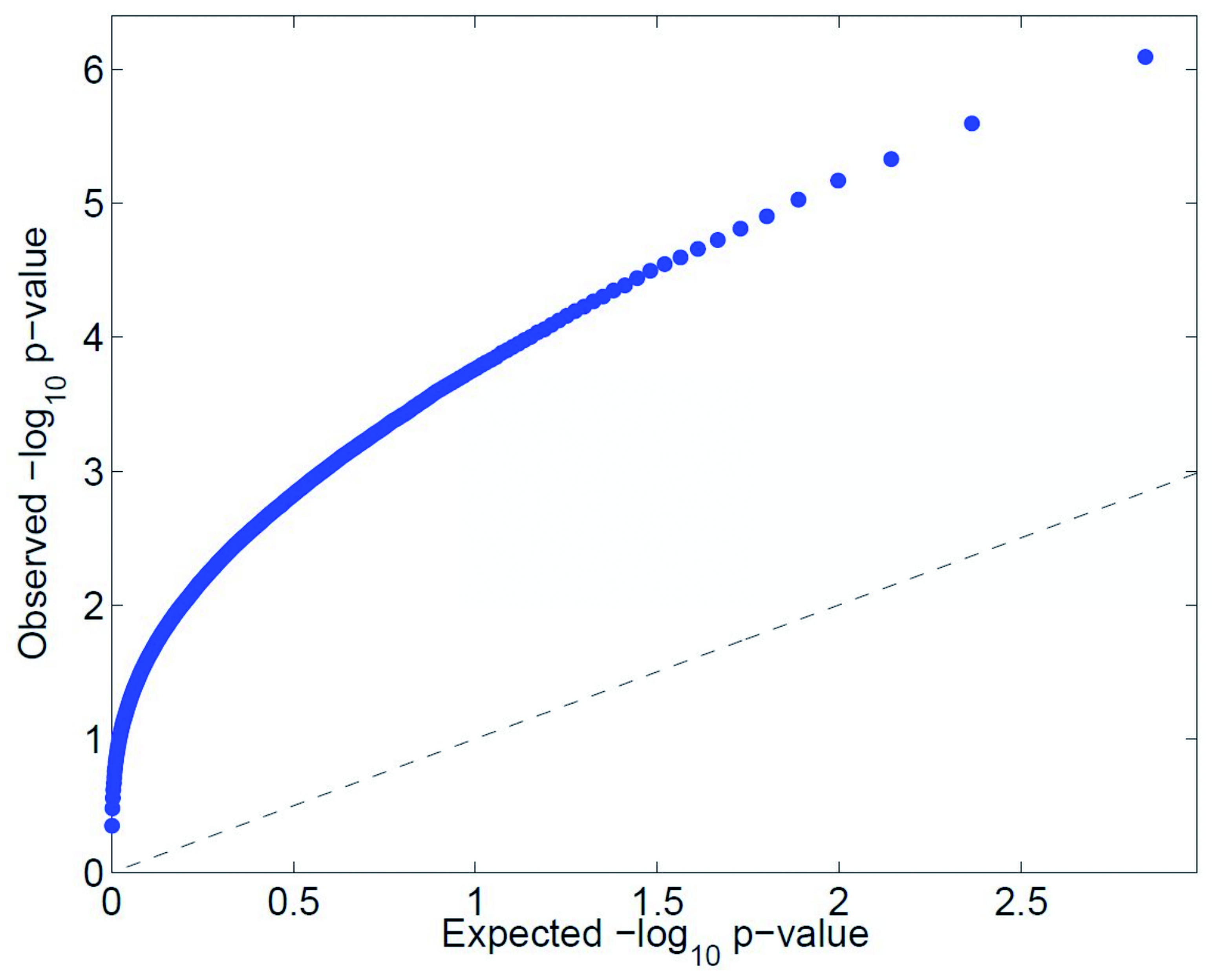

Supplement: Figure S5 — Inflated P-values of the sex-difference test due to the selection on sex-specific association. We have simulated 1 Million SNPs under the null hypothesis of no sex-difference (and no association), selected 348 SNPs with the most extreme sex-specific association, and plotted the observed P-values of the sex-difference test compared to the expected. It can be seen that the observed sex-difference P-values are inflated (i.e. do not lie on the identity line), which indicates that the sex-difference test is not independent from the sex-specific association selection. (TIFF) [file pgen.1003500.s005.tiff]
