# Supplementary material for: Sex-stratified Genome-wide Association Studies Including 270,000 Individuals Show Sexual Dimorphism in Genetic Loci for Anthropometric Traits
Source: PLoS Genet. 2013 Jun 6;9(6):e1003500. doi: 10.1371/journal.pgen.1003500 (PMC3674993; doi:10.1371/journal.pgen.1003500)
Supplement: Figure S2 — Region plot of the 7 identified loci showing the (a) association P-value in women, (b) association P-value in men, and (c) the sex difference P-value. (PDF) [file pgen.1003500.s002.pdf]

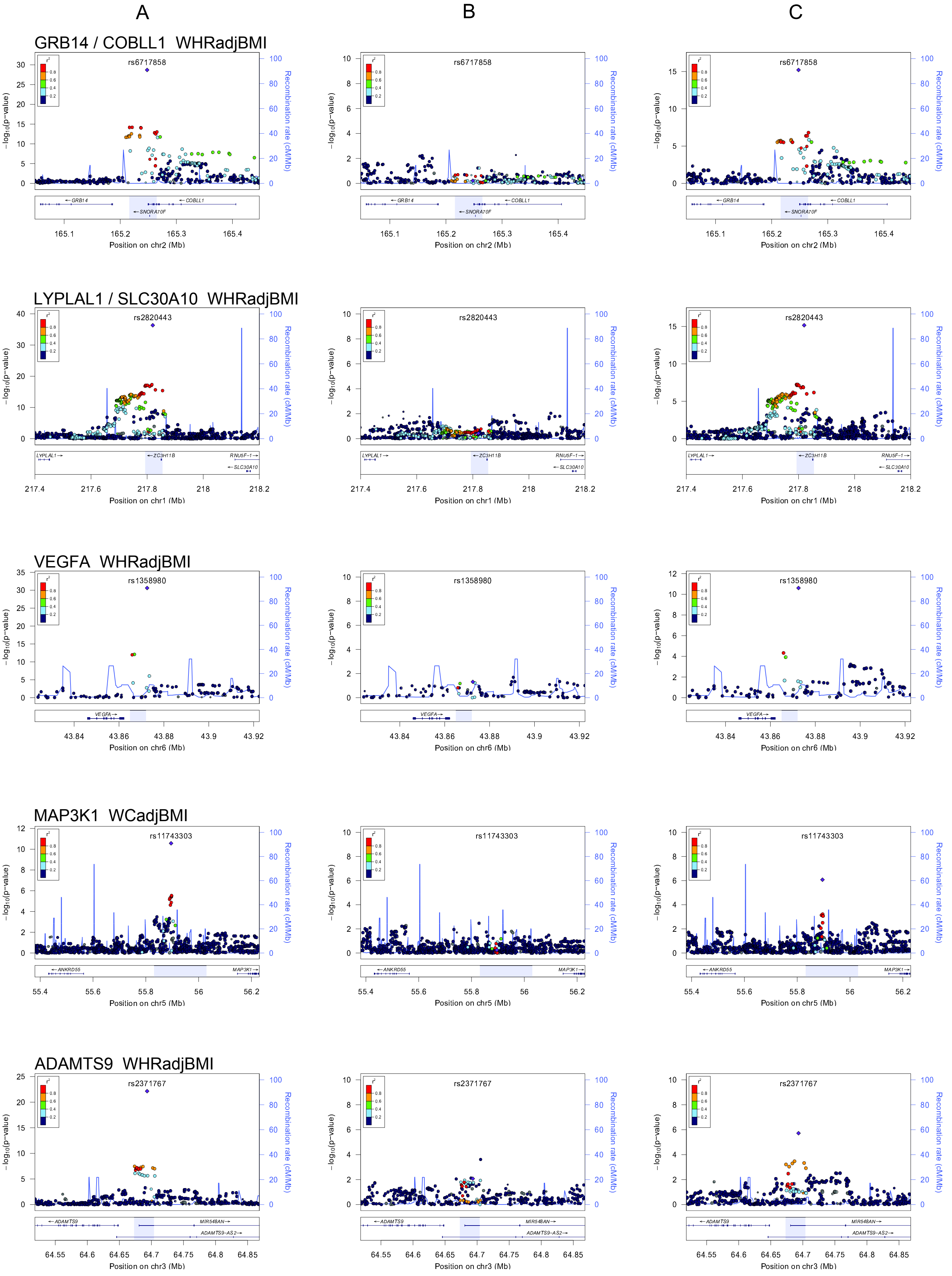
A
B
C
GRB14 / COBLL1 WHRadjBMI
rs6717858
GRB14
COBLL1
SNORA70F
Position on chr2 (Mb)
LYPLAL1 / SLC30A10 WHRadjBMI
rs2820443
LYPLAL1
ZC3H11B
RNU5F−1
SLC30A10
Position on chr1 (Mb)
VEGFA WHRadjBMI
rs1358980
VEGFA
Position on chr6 (Mb)
MAP3K1 WCadjBMI
rs11743303
ANKRD55
MAP3K1
Position on chr5 (Mb)
ADAMTS9 WHRadjBMI
rs2371767
ADAMTS9
MIR548AN
ADAMTS9−AS2
Position on chr3 (Mb)
−log10(p−value)
Recombination rate (cM/Mb)
r2

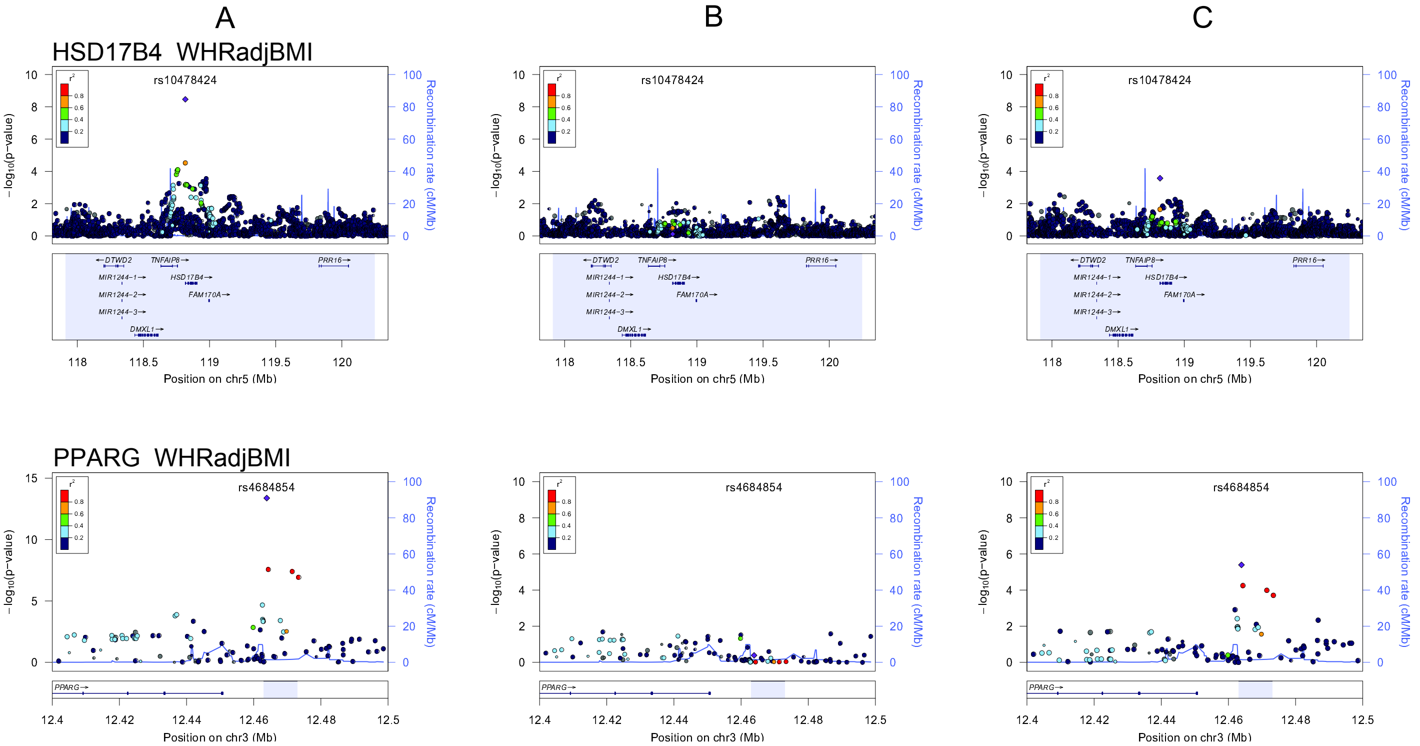
A
B
C
HSD17B4 WHRadjBMI
PPARG WHRadjBMI
rs10478424
rs4684854
−log10(p−value)
Recombination rate (cM/Mb)
r²
0.8
0.6
0.4
0.2
DTWD2
TNFAIP8
MIR1244−1
HSD17B4
MIR1244−2
FAM170A
MIR1244−3
DMXL1
PRR16
PPARG
Position on chr5 (Mb)
Position on chr3 (Mb)
