# Supplementary material for: Sex-stratified Genome-wide Association Studies Including 270,000 Individuals Show Sexual Dimorphism in Genetic Loci for Anthropometric Traits
Source: PLoS Genet. 2013 Jun 6;9(6):e1003500. doi: 10.1371/journal.pgen.1003500 (PMC3674993; doi:10.1371/journal.pgen.1003500)
Supplement: Text S1 — Supplementary note. (PDF) [file pgen.1003500.s019.pdf]

Text S1: Supplementary Note

Double genomic control correction in the meta-analysis

Sex-specific standard errors and P-values were genomic control (GC) corrected computing the study-specific lambda factor and a second GC correction was performed on the meta-analyzed result[50]. While all genome-wide available SNPs were used for GC correcting the *in-silico* studies, for metabochip studies, we only used a subset of 4,427 SNPs for GC correction: Most of the SNPs had been selected for the metabochip due to metabolic trait associations that would result in overestimated lambda factors, but these 4,427 SNPs selected for QT-interval association onto the metabochip were deemed to be least associated with anthropometric traits The lambda factors for GWAS have been reported previously[49,51,52] and the lambda factors for in-silico follow up studies ranged from 0.99 to 1.15 and for metabochip follow up studies from 0.89 to 1.13 except for one large study lambda=1.25.

Power considerations for the discovery

We evaluated the power of the two genome-wide screening approaches (the sex-specific scan and the sex-difference scan) to select sex-sensitive SNPs into follow-up[53]. For this, we assumed a certain genetic effect in women and varied the effect in men from OED to CED (**Figure S4A**). We found that the sex-specific scan had higher power for SSE signals (no effect in men) compared to the sex-difference scan, while the sex-difference scan had higher power for OED signals. It can also be seen that the overall scan (man and women combined) as currently usually applied in GWAS had good power for CED signals, but less for SSE and none for OED.

Specifically, to provide an example for the WHRadjBMI and height analyses in the discovery (34,629 men, 42,969 women for WHRadjBMI; 60,587 men, 73,137 women for height), the power of the sex-specific scan to select, at 5% FDR (corresponding to a P-value of $2x10^{-5}$ in our data), an SSE signal (i) such as the *LYPLAL1* SNP (b_{women} = 0.064, b_{men} = 0, MAF = 0.28, R^2_{women}= 0.00167, R^2_{men}=0) was 99%, (ii) for a signal such as the *PPARG* SNP (b_{women} = 0.034, b_{men} = 0, MAF=0.42, R^2_{women} = 0.00057, R^2_{men} = 0), the power was 81%, (iii) for a medium-sized height signal in one sex and none in the other (e.g. rs572169 from *GHSR*, b_{women} =0.030, b_{men} = 0, MAF = 0.31, R^2_{women}= 0.00039, R^2_{men} = 0, from Lango et al.), the power was 78%. We had 80% power to detect sex-specific signals for height with an R^2 of 0.000485 in women and 0 in men. The power via the sex-difference scan to select an OED signal at α = $2x10^{-5}$ with effect sizes that were (i) half of the *LYPLAL1* effect (signed R^2_{women} = + 0.000835 and signed R^2_{men} = -

0.000835) was 99%, (ii) for effect sizes half of the *PPARG* effect (signed R^2_{women} = +0.000285 and signed R^2_{men} = -0.000285), the power was 66%, (iii) for effect sizes half of a medium height signal (e.g. rs572169, signed R^2_{women} = + 0.000195, signed R^2_{men} = - 0.000195), the power was 79%. Note that the power to select such signals using the sex-specific scan was 98% (LYPLAL1), 21% (PPARG) or 32% (height gene GHSR), respectively.

Power considerations in the follow-up

When comparing the power of the sex-difference test of the 348 SNPs in the follow-up without and with a prior filter for a main effect (P-value of association combined in men and women < 0.01), it can be seen that there is a higher power with the prior filter for CED or SSE signals. This is due to the fact that the main effect filter utilizes the knowledge that the effect is not OED. For example, the power to detect a signal such as *PPARG* was 79% without the filter and 87% with the filter (see **Figure S4B**).

Specifically for the example of WHRadjBMI and height analyses (47,896 men, 60,936 women for WHRadjBMI; 62,395 men, 74,657 women for height), the power to establish sex-difference in our follow-up at 5% FDR (corresponding to a sex-difference P-value of $4.2x10^{-3}$ in our data) was (i) 99% for a women-only signal such as the *LYPLAL1*, (ii) 87% for a women-only signal such as *PPARG*, (iii) 78% for a women-only signal such as the height signal specified in the previous chapter. We had 80% power to establish a sex-difference with R^2_{women}=0.0045, R^2_{men}=0 for WHRadjBMI or R^2_{women}= 0.00035, R^2_{men}=0 for height.

Literature search and bio-informatic analyses regarding function of genes and variants at the seven loci

To explore any potentially functional elements underlying the regions of association (ranging from 7kbp to 2337kbp in size), we searched the UCSC and Ensembl genome browsers and found: (i) two of the seven regions (*GRB14/COBLL1* and *HSD17B4*) had one or more protein coding genes overlapping the region of the association signal (*COBLL1 at GRB14/COBLL1; DMXL1, DTWD2, FAM170A, HSD17B4, PRR16, and TNFAIP8 at HSD17B4*), making these seven protein-coding genes potential candidates to explain the observed association. (ii) Four of the seven regions (*GRB14/COBLL1, LYPLAL1/SLC30A10, ADAMTS9, and HSD17B4*) had a total of seven annotated non-coding transcripts including a snoRNA (SNORA70F at *GRB14/COBLL1*), a processed pseudogene (ZC3H11B at *LYPLAL1/SLC30A10*), a lincRNA (ADAMTS9-AS2 at *ADAMTS9*), and several microRNAs (MIR548AN at ADAMTS9; MIR1244-1, MIR1244-2, and MIR1244-3 at *HSD17B4*) overlapping

the region of association signal. (iii) In the remaining three loci (*VEGFA, PPARG, and MAP3K1*) we did not find any known coding genes or non-coding transcripts overlapping the regions of association. However, all three of these regions were relatively near to the protein-coding region of the genes (3.7kb downstream of VEGFA, 13kb downstream of PPARG, and 116kb upstream of MAP3K1) and thus could potentially be involved with cis-regulatory elements that have not yet been reported and/or annotated.

More details by locus as derived by searching several catalogues (copy number tagging SNPs, NHGRI GWAS), functional annotation data bases (SIFT, SNPinfo), as well as PubMed and OMIM, are given below:

1. ***GRB14 / COBLL1*** (2q24.3, selected for WHRadjBMI), lead marker rs6717858, with association signal extending across ~49kb of chromosome 2, ranging from 165216-165265kb. Two genes (*COBLL1* and *SNORA70F*) overlap this signal region, as does a previously reported SNP association with WHRadjBMI in tight LD with our lead marker (rs10195252: ~26.5kb, ~0.001cM, r^2=0.94, D'=1.0)[51]. Heid et al.[49] also presented eQTL data which suggested that growth factor receptor-bound protein 14 (*GRB14*) expression was associated with rs10195252 genotype and not *COBLL1*[51]. Our region lies ~30-79kb upstream of *GRB14*, which is a member of a family of SH2-containing adaptors. The *GRB14* protein binds directly to the insulin receptor[54,55], and likely has an inhibitory effect on receptor tyrosine kinase signaling as well as on insulin receptor signaling, thereby regulating growth and metabolism. *Grb14*-deficient mice exhibit enhanced body weight, mainly explained by increased lean mass on normal diet, improved glucose homeostasis despite lower circulating insulin levels, and enhanced insulin signaling in liver and skeletal muscle[56]. *Grb14* expression is increased in adipose tissue of insulin-resistant animal models and type 2 diabetic human patients[57], suggesting that *Grb14* may modulate insulin sensitivity. The WHR signal appears to be distinct from a *GRB14* locus previously reported as associated with both smoking initiation and current smoking (rs4423615: ~101kb, ~0.19cM, r^2<0.001, D'=0.01 with lead marker)[58]. Cordon-bleu protein-like 1 (*COBLL1*) may be involved in neural tube formation[59], is expressed at higher levels in tumors associated with good prognosis in mesothelioma after surgery[60], and its knockdown led to increased apoptosis in both normal and tumor cells[61]. Our bio/informatical analyses did not identify any potentially functional entity.

2. ***LYPLAL1 / SLC30A10*** (1q41, WHRadjBMI), lead marker rs2820443, with association signal extending across ~62kb of chromosome 1, ranging from 217793-217855kb. One gene (*ZC3H11B*) overlaps this signal region, as does a previously reported SNP associated with WHRadjBMI (rs4846567: ~2.8kb, ~0.0002cM, r^2 = 0.96, D' = 1.0 with lead marker)[51]. Zinc finger CCCH-type containing 11B (*ZC3H11B*) is a pseudogene with no known function. Another previously reported

SNP association with WHRadjBMI lies ~82-144kb from our association region (rs2605100: ~109kb, ~0.04cM, r^2 = 0.68, D' = 0.84 with lead marker)[62]. Excluding ZC3H11B, our signal region is nearest to *SLC30A10* (~299-361kb downstream) and *LYPLAL1* (~340-402kb downstream). Solute carrier family 30, member 10 (*SLC30A10*) belongs to a family of membrane transporters involved in intracellular zinc homeostasis and is expressed in brain and liver[63]. *LYPLAL1* encodes lysophospholipase-like 1 protein, which is thought to act as a triglyceride lipase and is reported to be up-regulated in subcutaneous adipose tissue of obese subjects[64]. Intergenic variants near *LYPLAL1* have also been associated with fatty liver disease (rs12137855: ~305kb, ~0.22cM, r^2 = 0.1, D' = 0.40 with lead marker)[65].

Our bio-informatical analyses found several SNPs moderately correlated with our lead SNP that are putative transcription factor binding sites (rs7538503: r^2 = 0.78, rs2605101: r^2 = 0.72, rs2605098: r^2 = 0.66, rs2605100: r^2 = 0.62).

3. ***VEGFA*** (6p21.1, WHRadjBMI), lead marker rs1358980, with association signal extending across ~7kb of chromosome 6, ranging from 43872-43865kb. No genes overlap this signal region, but it does include a SNP previously associated with WHRadjBMI (rs6905288: ~5.6kb, ~0.01cM, r^2 = 0.5, D' = 0.91 with lead marker)[49]. The associated region is located 3.7-10.7kb downstream of vascular endothelial growth factor A (*VEGFA*). Multiple variants and mutations in *VEGFA* are risk factors for diabetic retinopathy[66-68], and variants in *VEGFA* have been nominally associated with Type 2 Diabetes (T2D)[69]. *VEGFA* is proposed as a key mediator of adipogenesis and angiogenesis[70], is highly expressed in adipose tissue, and has increased expression during adipocyte differentiation[71-74]. *VEGFA* serum concentrations are elevated in overweight and obese patients compared with lean subjects[75] and decrease after weight loss following bariatric surgery, behaving similarly to other hormones related to adipose mass, such as leptin and insulin[76]. Variants near *VEGFA* have also been associated with kidney function (rs881858: ~42kb, 0.2cM, r^2 = 0.01, D' = 0.18 with lead marker)[77] and advanced age related macular degeneration (rs4711751: ~64kb, 0.2cM, r^2 = 0.04, D' = 0.21 with lead marker in 1000G data)[78] although both appear likely to be distinct from our signal.

Our bio/informatical analyses did not identify any potentially functional entity.

4. ***ADAMTS9*** (3p14.1, WHRadjBMI), lead marker rs2371767, with association signal extending across ~31kb of chromosome 3, ranging from 64704-64673kb. Two genes (*ADAMTS9-AS2* and *MIR548AN*) overlap this signal region, as do three previously reported SNP associations, one with WHRadjBMI (rs6795735: ~12.9kb, ~0.004cM, r^2=0.311, D'=1.0 with lead marker) [51], and two with T2D (rs4607103: ~6.4kb, ~0.001cM, r^2=0.90, D'=1.0; and rs4411878: ~14.6kb, ~0.005cM, r^2=0.85, D'=0.95)[69,79]. The T2D association is possibly mediated through decreased insulin sensitivity of

peripheral tissues[80]. *MIR548AN* is a microRNA which primarily maps to the X chromosome but also maps with 96.4% identity (full length transcript with 3 base mismatches) within our signal region. The function of *MIR548AN* is not known. *ADAMTS9-AS2* is a long non-coding RNA which is an antisense for *ADAMTS9*. Our region is located ~25-56kb upstream of *ADAMTS9*. *ADAMTS9* is a member of the disintegrin and metalloproteinase with thrombospondin motifs (ADAMTS) family, a group of genes encoding metalloproteases that lack transmembrane domains and are secreted into the extracellular matrix[81]. Members of the ADAMTS family have been implicated in control of organ shape during development and inhibition of angiogenesis[82].

Our bio/informatical analyses did not identify any pontially functional entity.

5. ***HSD17B4*** (5q23.1), lead marker rs10478424, with association signal extending across ~2337kb of chromosome 5, ranging from 117911-120249kb. Nine genes (*DMXL1*, *DTWD2*, *FAM170A*, *HSD17B4*, *MIR1244-1*, *MIR1244-2*, *MIR1244-3*, *PRR16*, and *TNFAIP8*) overlap this signal region. The lead marker, rs10478424, is located in an intronic region of hydroxysteroid (17-beta) dehydrogenase 4 (*HSD17B4*). The protein encoded by *HSD17B4* is a bifunctional enzyme that is involved in the peroxisomal beta-oxidation pathway for fatty acids. Mutations in this gene are known to cause DBP deficiency, an autosomal-recessive disorder of peroxisomal fatty acid beta-oxidation that is generally fatal within the first two years of life[83,84]. Expression levels of *HSD17B4* have been associated with prostate cancer severity[85], and it is also a significant independent predictor of poor patient outcome[86].

Our bio-informatical analyses found that our lead marker is in a predicted transcription factor binding site (TFBS) and could therefore potentially influence regulation of transcription of an alternative putative protein-coding splice variant of HSD17B4. Interestingly, one of the transcription factors predicted to bind at this site is PPARG, which itself is located near one of the other association regions reported here. In addition, several proxies in moderate LD with the lead marker disrupt predicted microRNA bindings sites (rs1045241: r^2 = 0.53, rs1045242: r^2 = 0.53, rs11064: r^2 = 0.53) and are also possible candidates for functional explanations of the association signal at this locus. It should also be noted that there was one CNV tagging SNP (rs1948325) in that region that showed nominal significance in women (P=0.054, P=0.281) consistent with the women-only association signal of WHRadjBMI, but this would not hold significance when accounting for the multiple testing conducted in the CNV tagging SNP analysis (6016 SNPs tested).

6. ***PPARG*** (3p25.1, WHRadjBMI), lead marker rs4684854, with association signal extending across ~10kb of chromosome 3, ranging from 12463-12473kb. No known genes overlap this region. However, it does lie approximately 13kb downstream of the well known type 2 diabetes susceptibility gene, peroxisome proliferator-activated receptor gamma (*PPARG*), although the T2D

associated SNP appears to be distinct from our locus (rs1801282: ~96kb, ~0.11cM, r^2=0.04, D'=0.61 with lead marker)[26,87,88]. The protein product encoded by *PPARG* is PPAR-gamma which is a regulator of adipocyte differentiation. Additionally, PPAR-gamma has been implicated in the pathology of numerous diseases including obesity[89-92], diabetes[93,94], atherosclerosis[95] and cancer[93,96-98]. Interestingly, previous studies of the Pro12Ala polymorphism in the PPAR gene have demonstrated genotype–by-sex interaction with BMI[99], fatty acid concentrations during the first 24h after birth were related to *PPARG* expression in female but not in male lambs[100], and female 12Ala mutation carriers had greater risk of developing abdominal obesity than female non-carriers while male 12Ala mutation carriers had no significant increase in risk[101].

Our bio/informatical analyses did not identify any potentially functional entity.

7. ***MAP3K1*** (5q11.2, WCadjBMI), lead marker rs11743303, with association signal extending across ~198kb of chromosome 5, ranging from 55832-56030kb. No known genes overlap this region, but it does overlap with another SNP (rs6867983: ~5.8kb, 0.02cM, r^2=0.71, D'=1.0 with lead marker) reported as a suggestive association with triglyceride level[102]. The associated region also lies ~116-314kb upstream of mitogen-activated protein kinase kinase kinase 1 (*MAP3K1*), a serine/threonine kinase that occupies a pivotal role in a network of phosphorylating enzymes integrating cellular responses to a number of mitogenic and metabolic stimuli, including insulin (MIM 176730) and many growth factors[82]. Mutations in *MAP3K1* are associated with gonadal dysgenesis[103], and a SNP within *MAP3K1* (rs889312: ~172kb, 0.44cM, r^2=0.005, D'=0.10 with lead marker) has been reported to be associated with breast cancer[104,105], possibly as a gene-gene interaction with *BRCA2*[106]. It is also ~384-582kb upstream of ankyrin repeat domain 55 (*ANKRD55*), which harbors SNPs reported to be associated with longevity (rs415407: ~445kb, 1.1cM, r^2=0.005, D'=0.12 with lead marker)[107], Rheumatoid Arthritis (rs6859219: ~421kb, 0.93cM, r^2=0.008, D'=0.10 with lead marker)[108], and Celiac Disease (rs1020388: ~300kb, 0.71cM, r^2=0.003, D'=0.11 with lead marker)[109].

Our bio/informatical analyses did not identify any pontially functional entity.

Human tissue eQTL

Subcutaneous adipose tissue and whole blood from deCode: As described previously[110], 603 (252 males, 351 females) individuals with adipose tissue and 747 (312 males, 435 females) individuals with whole blood samples were genotyped with the Illumina 317K or 370K platform and HapMap imputation performed. Gene expression profiles were conducted using RNA from the adipose and blood samples using a custom-made human array with 23,720 unique oligonucleotide probes. *Cis* associations were tested separately by gender by regressing the

mean logarithm (log10) expression ratio (MLR) on the number of effect alleles adjusting for age (and the differential cell count for the blood analyses) and accounting for familial relatedness. Only *cis* associations with a P-value < $1x10^{-5}$ corresponding to an FDR < 5% in either sex are reported.

Subcutaneous adipose tissue and whole blood from MolOBB: Seventy-three individuals were recruited to donate subcutaneous adipose tissue from the abdominal wall and gluteus [In press Plos Genetics]. Total RNA was extracted from the fat tissue using TRIreagent and hybridized onto the Affymetrix Human Genome U133 Plus 2.0 gene-expression microarrays (hgu133plus2 arrays), containing 17,726 non-overlapping probes. Subjects were genotyped with the Illumina 317K Beadchip chip array and imputation conducted using IMPUTE. After quality control filters were applied to the expression and genotype data, 52 individuals (31 male, 21 female) with abdominal adipose tissue, 62 subjects with gluteal fat (35 male, 27 female), and 65 subjects with whole blood (36 male, 29 female) remained for eQTL analysis. *Cis* associations within 500kb of each gene were evaluated by regressing expression level on genotype and adjusting for plate effects. Two models were tested: 1) assuming the same slope in each gender (e.g., gender-homogeneity effects) and 2) assuming a different slope for each gender (e.g., gender-specific effects). Only those associations with an FDR < 1% in either sex are presented.

Lymphoblastoid cell lines from a childhood asthma study. As described previously [111], peripheral blood lymphocytes were transformed into lymphoblastoid cell lines for 206 families of European descent (214 male, 181 female). Using extracted RNA, gene expression was assessed with the Affymetrix HG-U133 Plus 2.0 chip. Genotyping was conducted using the Illumina Human1M Beadchip and Illumina HumanHap300K Beadchip, and imputation performed using data from Phase II HapMap CEU population. SNPs were tested for *cis* associations (defined as genes within 1 Mb), adjusting for non-genetic effects in the gene expression value. Only cis associations that reached a $P<6.8x10^{-5}$ (FDR of 1%) in either sex were included in the table.

Lymphoblastoid cell lines from the International HapMap Project: As described previously[112], transcription profiling was done on Epstein-Barr virus-transformed lymphoblastoid cell lines from the original 379 individuals in the four HapMap populations (CEU: 54 females, 55 males; CHB: 42 females, 38 males; JPT: 40 females, 42 males; YRI: 53 females, 55 males) using the Illumina human whole-genome expression (WG-6 version 1) arrays, which contain 47,294 probes. The genotype data from HapMap 3 was used to evaluate the associations with expression. *Cis* associations within a 2 Mb window centered on the gene were tested using

Spearman rank correlation[112], stratified by sex and population. A threshold of $P < 1.0 \times 10^{-5}$ was used to determine nominal significance, but no associations reached this level.

Mouse expression

There were three experiments on mouse expression in Regensburg, Oxford and Houston. The genes explored via PCR in Regensburg are given in **Table S13A**, the genes explored via Illumina array in Oxford and Houston in **Table S13B**. Details of the three experiments are given below:

Mouse experiment at the Regensburg center:

At the Regensburg University, mice (7 female and 7 male animals) were purchased from Charles River Laboratories (Sulzfeld, Germany) at an age of 7 weeks. Body weight of the female mice was 16.7 + 0.7 g and of the male mice 20.3 + 1.4 g (p = 0.001). After 3 weeks of acclimatisation, animals were killed and respective organs were immediately removed. Total RNA was isolated with TRIzol reagent from GIBCO (Carlsbad, CA), oligonucleotides used for amplification using LightCycler technology were synthesized by Metabion (Planegg-Martinsried, Germany) and are listed in the table. Real-time RT-PCR was performed as recently described[113], and the specificity of the PCRs was confirmed by sequencing of the amplified DNA fragments (Geneart, Regensburg,Germany). For quantification of the results, RNA was reverse transcribed, and cDNA was serially diluted and used to create a standard curve for each of the genes analyzed. The second-derivative maximum method was used for quantification with the LightCycler software. Values were normalized to 18s rRNA expression and are given as arbitrary units.

Mouse experiment at the Oxford center:

Animals: Original Northport Heterogeneous Stock (HS) mice were obtained from Robert Hitzemann of the Oregon Health Sciences Unit (Portland, Oregon). At the time the animals arrived, they had descended from 50 generations of pseudorandom breeding that commenced with eight founder strains: A/J, AKR/J, BALBc/J, CBA/J, C3H/HeJ, C57BL/6J, DBA/2J, and LP/J[114]. The animals were bred for phenotyping in a colony established at the University of Oxford. They were housed at a maximum of six per cage (median of four) and maintained on a 12:12 light:dark cycle with *ad libitum* access to food and water.

Gene Expression Assays: From a pool of over 1940 HS mice (1000 males), the most unrelated animals (based on genome-wide genotyping of 13.5k SNPs using Illumina's BeadArray platform[115]) were assayed for gene expression in several tissues, including the liver (n = 273

with 139 males). As detailed by Huang and colleagues[116], the tissues were frozen in liquid nitrogen and homogenised. RNA was extracted from the tissue and mRNA was amplified. Labelled mRNA was hybridised to the Illumina Mouse WG-6 v1 BeadArray platform, which contains 47.4k unique RNA probe sequences.

Gene Expression Data Preprocessing: To extract data from the images produced by the BeadArray platform, the images were imported into the Gene Expression module (V 1.6.0) of the Illumina GenomeStudio (V 2010.1) without invoking any data adjustment procedures. The data were exported to R[117] using the Bioconductor package *lumi*[118], where one outlying liver array and five hippocampus arrays, as visualised in cluster dendrograms, were removed from further analysis. Subsequently, *lumi* was employed to subtract background noise from the arrays, and apply variance stabilising transformation and robust spline normalisation. Only probes identified by Barbosa-Morais and co-workers[119] as “good” or “perfect” matches to the genome, and only probes expressed in at least 5% of animals at a 0.95 detection level (as per GenomeStudio), were retained, leaving 13718 liver and 15737 hippocampus probes. Annotations aligning the probes with physical locations of the mouse genome were also obtained from Barbosa-Morais and colleagues. ComBat was used to remove batch effects[120].

Mapping human genes to mouse genes: We first mapped human gene names to Ensembl gene IDs using the DAVID gene ID conversion tool[116] (http://david.abcc.ncifcrf.gov/). We manually replaced three genes which have alternate names (*ATAD4* with *PRR15L*, *FBXL10* with *KDM2B*, and *MIRHG1* with *MIR17HG*) and could not convert *NIACR2*. Then we looked for mouse orthologs using the BioMart webservice (http://www.biomart.org/), keeping only genes with one to one orthologs. We found mouse orthologs for 134 out of 156 human genes at 18 loci.

Testing for Sex-Specific Gene Expression: All probes corresponding to the list of mouse genes of interest were fit into a linear model.

Mouse experiment at the Houston center:

Animals: The study was performed using 21 male, 21 female C57/BL6 mice fed from day 21 (after weaning) or 12 weeks on an HF diet (4.7 Kcal g–1 and 45% calories from fat; Research Diets, Inc., New Brunswick, NJ, US. Mice were single caged and maintained at a controlled temperature (22 °C) and a 12 h light–dark cycle (light on from 0800 to 2000 h). Daily food intake and body weight were monitored. All procedures were approved by the animal care and use committee of the University of Cincinnati.

Tissue collection: After 12 weeks of exposure to the diet, animals were killed during the first 2 h of the beginning of the light cycle after a 12-h fast. All females were killed in the proestrus phase of their estrus cycle. Two different WAT depots, FGWAT and IWATF, were rapidly dissected

and the orientation of the fat pad was maintained. Half of the fat pad was rapidly frozen for microarray analysis, and the other half of the fat pad was rapidly frozen for validation of target genes. Samples were rapidly frozen in liquid nitrogen and stored at 70 °C for RNA analysis.

RNA isolation: GWAT/IWAT were homogenized in 800 ml Trizol reagent (Invitrogen, Carlsbad, CA, USA), and total cellular RNA was isolated according to the specifications of the manufacturer. Total RNA was further purified using the RNeasy Mini kit (Qiagen, Valencia, CA, USA). The quality and concentration of the RNA was determined by measuring the absorbance at 260 and 280 nm, and RNA integrity was confirmed by bioanalysis (Agilent 2100 Bioanalyzer; Agilent Technologies,Santa Clara, CA, USA).

Microarray analysis: For the microarray analysis, seven independent pooled samples were analyzed from GWAT and IWAT fat pads. Each sample comprised a pool of tissue from three animals (for pooled samples, reverse transcription was performed on each sample individually and equal amounts of complementary DNA (cDNA) were pooled); thus, samples were obtained from a total of 21 mice. GWAT/IWAT adipose RNA pools were generated from the following groups of mice: males and females. To identify genes that were differentially expressed in the two WAT fat pads, comparisons were made between normalized signal intensity from the control group (males) and experimental groups (female) from each experiment. Each total RNA sample was processed according to protocols recommended by the manufacturers. In brief, total RNA is reverse-transcribed with an oligo-dT primer and double-stranded cDNA is generated. The cDNA serves as a template for the in vitro transcription reaction that produces amplified amounts of biotin-labeled antisense mRNA. This biotinylated RNA is referred to as labeled cRNA. After fragmentation, the cRNA is hybridized onto oligonucleotide microarrays (Mouse Genome 430 2.0; 40 000 mouse probe sets). A GeneChip Operating System absolute expression analysis was performed for each Gene-Chip genome array hybridization. After the initial analysis, the absolute analysis was re-run using global scaling to an average target intensity of 350. The scaling allows for the direct comparison of hybridization values from the different targets analyzed in this project (and with any additional GeneChip sample assays using the same array type).

REFERENCES

1. Stolk RP, Rosmalen JG, Postma DS, de Boer RA, Navis G, et al. (2008) Universal risk factors for multifactorial diseases: LifeLines: a three-generation population-based study. European journal of epidemiology 23: 67-74.

2. Pinto-Sietsma SJ, Janssen WM, Hillege HL, Navis G, De Zeeuw D, et al. (2000) Urinary albumin excretion is associated with renal functional abnormalities in a nondiabetic population. Journal of the American Society of Nephrology : JASN 11: 1882-1888.
3. Medland SE, Nyholt DR, Painter JN, McEvoy BP, McRae AF, et al. (2009) Common variants in the trichohyalin gene are associated with straight hair in Europeans. American journal of human genetics 85: 750-755.
4. Cornelis MC, Monda KL, Yu K, Paynter N, Azzato EM, et al. (2011) Genome-wide meta-analysis identifies regions on 7p21 (AHR) and 15q24 (CYP1A2) as determinants of habitual caffeine consumption. PLoS genetics 7: e1002033.
5. Go AS, Iribarren C, Chandra M, Lathon PV, Fortmann SP, et al. (2006) Statin and beta-blocker therapy and the initial presentation of coronary heart disease. Annals of internal medicine 144: 229-238.
6. Taylor-Piliae RE, Norton LC, Haskell WL, Mahbouda MH, Fair JM, et al. (2006) Validation of a new brief physical activity survey among men and women aged 60-69 years. American journal of epidemiology 164: 598-606.
7. Iribarren C, Go AS, Husson G, Sidney S, Fair JM, et al. (2006) Metabolic syndrome and early-onset coronary artery disease: is the whole greater than its parts? Journal of the American College of Cardiology 48: 1800-1807.
8. Assimes TL, Knowles JW, Basu A, Iribarren C, Southwick A, et al. (2008) Susceptibility locus for clinical and subclinical coronary artery disease at chromosome 9p21 in the multi-ethnic ADVANCE study. Human molecular genetics 17: 2320-2328.
9. Samani NJ, Deloukas P, Erdmann J, Hengstenberg C, Kuulasmaa K, et al. (2009) Large scale association analysis of novel genetic loci for coronary artery disease. Arteriosclerosis, thrombosis, and vascular biology 29: 774-780.
10. Strachan DP, Rudnicka AR, Power C, Shepherd P, Fuller E, et al. (2007) Lifecourse influences on health among British adults: effects of region of residence in childhood and adulthood. International journal of epidemiology 36: 522-531.
11. Barrett JC, Clayton DG, Concannon P, Akolkar B, Cooper JD, et al. (2009) Genome-wide association study and meta-analysis find that over 40 loci affect risk of type 1 diabetes. Nature genetics 41: 703-707.
12. (2007) Genome-wide association study of 14,000 cases of seven common diseases and 3,000 shared controls. Nature 447: 661-678.
13. Power C, Elliott J (2006) Cohort profile: 1958 British birth cohort (National Child Development Study). International journal of epidemiology 35: 34-41.
14. James AL, Knuiman MW, Divitini ML, Hui J, Hunter M, et al. (2010) Changes in the prevalence of asthma in adults since 1966: the Busselton health study. The European respiratory journal : official journal of the European Society for Clinical Respiratory Physiology 35: 273-278.
15. Samani NJ, Erdmann J, Hall AS, Hengstenberg C, Mangino M, et al. (2007) Genomewide association analysis of coronary artery disease. The New England journal of medicine 357: 443-453.
16. Kotronen A, Yki-Jarvinen H, Mannisto S, Saarikoski L, Korpi-Hyovalti E, et al. (2010) Non-alcoholic and alcoholic fatty liver disease - two diseases of affluence associated with the metabolic syndrome and type 2 diabetes: the FIN-D2D survey. BMC public health 10: 237.
17. Konttinen H, Mannisto S, Sarlio-Lahteenkorva S, Silventoinen K, Haukkala A (2010) Emotional eating, depressive symptoms and self-reported food consumption. A population-based study. Appetite 54: 473-479.
18. Inouye M, Silander K, Hamalainen E, Salomaa V, Harald K, et al. (2010) An immune response network associated with blood lipid levels. PLoS genetics 6.

19. Tuomilehto J, Lindstrom J, Eriksson JG, Valle TT, Hamalainen H, et al. (2001) Prevention of type 2 diabetes mellitus by changes in lifestyle among subjects with impaired glucose tolerance. The New England journal of medicine 344: 1343-1350.
20. Kouki R, Schwab U, Lakka TA, Hassinen M, Savonen K, et al. (2010) Diet, fitness and metabolic syndrome - The DR's EXTRA Study. Nutrition, metabolism, and cardiovascular diseases : NMCD.
21. Fowkes FG, Housley E, Cawood EH, Macintyre CC, Ruckley CV, et al. (1991) Edinburgh Artery Study: prevalence of asymptomatic and symptomatic peripheral arterial disease in the general population. International journal of epidemiology 20: 384-392.
22. Nelis M, Esko T, Magi R, Zimprich F, Zimprich A, et al. (2009) Genetic structure of Europeans: a view from the North-East. PloS one 4: e5472.
23. Forouhi NG, Luan J, Hennings S, Wareham NJ (2007) Incidence of Type 2 diabetes in England and its association with baseline impaired fasting glucose: the Ely study 1990-2000. Diabetic medicine : a journal of the British Diabetic Association 24: 200-207.
24. Day N, Oakes S, Luben R, Khaw KT, Bingham S, et al. (1999) EPIC-Norfolk: study design and characteristics of the cohort. European Prospective Investigation of Cancer. British journal of cancer 80 Suppl 1: 95-103.
25. Willer CJ, Speliotes EK, Loos RJ, Li S, Lindgren CM, et al. (2009) Six new loci associated with body mass index highlight a neuronal influence on body weight regulation. Nature genetics 41: 25-34.
26. Scott LJ, Mohlke KL, Bonnycastle LL, Willer CJ, Li Y, et al. (2007) A genome-wide association study of type 2 diabetes in Finns detects multiple susceptibility variants. Science 316: 1341-1345.
27. Renstrom F, Payne F, Nordstrom A, Brito EC, Rolandsson O, et al. (2009) Replication and extension of genome-wide association study results for obesity in 4923 adults from northern Sweden. Human molecular genetics 18: 1489-1496.
28. Hallmans G, Agren A, Johansson G, Johansson A, Stegmayr B, et al. (2003) Cardiovascular disease and diabetes in the Northern Sweden Health and Disease Study Cohort - evaluation of risk factors and their interactions. Scandinavian journal of public health Supplement 61: 18-24.
29. Kimber CH, Doney AS, Pearson ER, McCarthy MI, Hattersley AT, et al. (2007) TCF7L2 in the Go-DARTS study: evidence for a gene dose effect on both diabetes susceptibility and control of glucose levels. Diabetologia 50: 1186-1191.
30. Stang A, Moebus S, Dragano N, Beck EM, Mohlenkamp S, et al. (2005) Baseline recruitment and analyses of nonresponse of the Heinz Nixdorf Recall Study: identifiability of phone numbers as the major determinant of response. European journal of epidemiology 20: 489-496.
31. Holmen J, Midthjell K, Krüger Ø, Langhammer A, Holmen TL, et al. (2003) The Nord-Trøndelag Health Study 1995-97 (HUNT 2): Objectives, contents, methods and participation. Science And Technology 13: 19-32.
32. Baldassarre D, Nyyssonen K, Rauramaa R, de Faire U, Hamsten A, et al. (2010) Cross-sectional analysis of baseline data to identify the major determinants of carotid intima-media thickness in a European population: the IMPROVE study. European heart journal 31: 614-622.
33. Wichmann H, Gieger C, Illig T (2005) KORA-gen-resource for population genetics, controls and a broad spectrum of disease phenotypes. Gesundheitswesen 67: 26.
34. Winkelmann BR, Marz W, Boehm BO, Zotz R, Hager J, et al. (2001) Rationale and design of the LURIC study--a resource for functional genomics, pharmacogenomics and long-term prognosis of cardiovascular disease. Pharmacogenomics 2: S1-73.
35. Stancakova A, Javorsky M, Kuulasmaa T, Haffner SM, Kuusisto J, et al. (2009) Changes in insulin sensitivity and insulin release in relation to glycemia and glucose tolerance in 6,414 Finnish men. Diabetes 58: 1212-1221.
36. Evans A, Salomaa V, Kulathinal S, Asplund K, Cambien F, et al. (2005) MORGAM (an international pooling of cardiovascular cohorts). International journal of epidemiology 34: 21.

37. Ferrario M, Chiodini P, Chambless LE, Cesana G, Vanuzzo D, et al. (2005) Prediction of coronary events in a low incidence population. Assessing accuracy of the CUORE Cohort Study prediction equation. International journal of epidemiology 34: 413-421.
38. Ferrario M, Sega R, Chatenoud L, Mancia G, Mocarelli P, et al. (2001) Time trends of major coronary risk factors in a northern Italian population (1986-1994). How remarkable are socioeconomic differences in an industrialized low CHD incidence country? International journal of epidemiology 30: 285-297.
39. Ferrario MM, Veronesi G, Chambless LE, Sega R, Fornari C, et al. (2011) The contribution of major risk factors and job strain to occupational class differences in coronary heart disease incidence: the MONICA Brianza and PAMELA population-based cohorts. Occup Environ Med 68: 717-722.
40. Wadsworth M, Kuh D, Richards M, Hardy R (2006) Cohort Profile: The 1946 National Birth Cohort (MRC National Survey of Health and Development). International journal of epidemiology 35: 49-54.
41. Hardy R, Wills AK, Wong A, Elks CE, Wareham NJ, et al. (2010) Life course variations in the associations between FTO and MC4R gene variants and body size. Human molecular genetics 19: 545-552.
42. Lind L, Fors N, Hall J, Marttala K, Stenborg A (2005) A comparison of three different methods to evaluate endothelium-dependent vasodilation in the elderly: the Prospective Investigation of the Vasculature in Uppsala Seniors (PIVUS) study. Arteriosclerosis, thrombosis, and vascular biology 25: 2368-2375.
43. Lichtenstein P, Sullivan PF, Cnattingius S, Gatz M, Johansson S, et al. (2006) The Swedish Twin Registry in the third millennium: an update. Twin research and human genetics : the official journal of the International Society for Twin Studies 9: 875-882.
44. Theodoraki EV, Nikopensius T, Suhorutsenko J, Peppes V, Fili P, et al. (2010) Fibrinogen beta variants confer protection against coronary artery disease in a Greek case-control study. BMC medical genetics 11: 28.
45. Jacobsen BK, Eggen AE, Mathiesen EB, Wilsgaard T, Njolstad I (2011) Cohort profile: The Tromso Study. International journal of epidemiology.
46. Ingelsson E, Sundstrom J, Arnlov J, Zethelius B, Lind L (2005) Insulin resistance and risk of congestive heart failure. JAMA : the journal of the American Medical Association 294: 334-341.
47. Marmot M, Brunner E (2005) Cohort Profile: the Whitehall II study. International journal of epidemiology 34: 251-256.
48. Jensen AC, Barker A, Kumari M, Brunner EJ, Kivimaki M, et al. (2011) Associations of common genetic variants with age-related changes in fasting and postload glucose: evidence from 18 years of follow-up of the Whitehall II cohort. Diabetes 60: 1617-1623.
49. Heid IM, Jackson AU, Randall JC, Winkler TW, Qi L, et al. (2010) Meta-analysis identifies 13 new loci associated with waist-hip ratio and reveals sexual dimorphism in the genetic basis of fat distribution. Nature genetics 42: 949-960.
50. Devlin B, Roeder K (1999) Genomic control for association studies. Biometrics 55: 997-1004.
51. Speliotes EK, Willer CJ, Berndt SI, Monda KL, Thorleifsson G, et al. (2010) Association analyses of 249,796 individuals reveal 18 new loci associated with body mass index. Nature genetics 42: 937-948.
52. Lango Allen H, Estrada K, Lettre G, Berndt SI, Weedon MN, et al. (2010) Hundreds of variants clustered in genomic loci and biological pathways affect human height. Nature 467: 832-838.
53. Behrens G, Winkler TW, Gorski M, Leitzmann MF, Heid IM (2011) To stratify or not to stratify: power considerations for population‚Äêbased genome‚Äêwide association studies of quantitative traits. Genetic epidemiology 35: 867-879.

54. Holt LJ, Siddle K (2005) Grb10 and Grb14: enigmatic regulators of insulin action--and more? The Biochemical journal 388: 393-406.
55. Depetris RS, Hu J, Gimpelevich I, Holt LJ, Daly RJ, et al. (2005) Structural basis for inhibition of the insulin receptor by the adaptor protein Grb14. Molecular cell 20: 325-333.
56. Cooney GJ, Lyons RJ, Crew AJ, Jensen TE, Molero JC, et al. (2004) Improved glucose homeostasis and enhanced insulin signalling in Grb14-deficient mice. The EMBO journal 23: 582-593.
57. Cariou B, Capitaine N, Le Marcis V, Vega N, Bereziat V, et al. (2004) Increased adipose tissue expression of Grb14 in several models of insulin resistance. The FASEB journal : official publication of the Federation of American Societies for Experimental Biology 18: 965-967.
58. Vink JM, Smit AB, de Geus EJ, Sullivan P, Willemsen G, et al. (2009) Genome-wide association study of smoking initiation and current smoking. American journal of human genetics 84: 367-379.
59. Carroll EA, Gerrelli D, Gasca S, Berg E, Beier DR, et al. (2003) Cordon-bleu is a conserved gene involved in neural tube formation. Developmental biology 262: 16-31.
60. Gordon GJ, Jensen RV, Hsiao LL, Gullans SR, Blumenstock JE, et al. (2003) Using gene expression ratios to predict outcome among patients with mesothelioma. Journal of the National Cancer Institute 95: 598-605.
61. Gordon GJ, Bueno R, Sugarbaker DJ (2011) Genes associated with prognosis after surgery for malignant pleural mesothelioma promote tumor cell survival in vitro. BMC cancer 11: 169.
62. Lindgren CM, Heid IM, Randall JC, Lamina C, Steinthorsdottir V, et al. (2009) Genome-wide association scan meta-analysis identifies three Loci influencing adiposity and fat distribution. PLoS genetics 5: e1000508.
63. Seve M, Chimienti F, Devergnas S, Favier A (2004) In silico identification and expression of SLC30 family genes: an expressed sequence tag data mining strategy for the characterization of zinc transporters' tissue expression. BMC Genomics 5: 32.
64. Steinberg GR, Kemp BE, Watt MJ (2007) Adipocyte triglyceride lipase expression in human obesity. Am J Physiol Endocrinol Metab 293: E958-964.
65. Speliotes EK, Yerges-Armstrong LM, Wu J, Hernaez R, Kim LJ, et al. (2011) Genome-wide association analysis identifies variants associated with nonalcoholic fatty liver disease that have distinct effects on metabolic traits. PLoS genetics 7: e1001324.
66. Watanabe D, Suzuma K, Suzuma I, Ohashi H, Ojima T, et al. (2005) Vitreous levels of angiopoietin 2 and vascular endothelial growth factor in patients with proliferative diabetic retinopathy. Am J Ophthalmol 139: 476-481.
67. Abhary S, Burdon KP, Gupta A, Lake S, Selva D, et al. (2009) Common sequence variation in the VEGFA gene predicts risk of diabetic retinopathy. Invest Ophthalmol Vis Sci 50: 5552-5558.
68. Al-Kateb H, Boright AP, Mirea L, Xie X, Sutradhar R, et al. (2008) Multiple superoxide dismutase 1/splicing factor serine alanine 15 variants are associated with the development and progression of diabetic nephropathy: the Diabetes Control and Complications Trial/Epidemiology of Diabetes Interventions and Complications Genetics study. Diabetes 57: 218-228.
69. Zeggini E, Scott LJ, Saxena R, Voight BF, Marchini JL, et al. (2008) Meta-analysis of genome-wide association data and large-scale replication identifies additional susceptibility loci for type 2 diabetes. Nature genetics 40: 638-645.
70. Nishimura S, Manabe I, Nagasaki M, Hosoya Y, Yamashita H, et al. (2007) Adipogenesis in obesity requires close interplay between differentiating adipocytes, stromal cells, and blood vessels. Diabetes 56: 1517-1526.
71. Claffey KP, Wilkison WO, Spiegelman BM (1992) Vascular endothelial growth factor. Regulation by cell differentiation and activated second messenger pathways. J Biol Chem 267: 16317-16322.

72. Zhang QX, Magovern CJ, Mack CA, Budenbender KT, Ko W, et al. (1997) Vascular endothelial growth factor is the major angiogenic factor in omentum: mechanism of the omentum-mediated angiogenesis. J Surg Res 67: 147-154.
73. Soukas A, Socci ND, Saatkamp BD, Novelli S, Friedman JM (2001) Distinct transcriptional profiles of adipogenesis in vivo and in vitro. J Biol Chem 276: 34167-34174.
74. Emoto M, Anno T, Sato Y, Tanabe K, Okuya S, et al. (2001) Troglitazone treatment increases plasma vascular endothelial growth factor in diabetic patients and its mRNA in 3T3-L1 adipocytes. Diabetes 50: 1166-1170.
75. Silha JV, Krsek M, Sucharda P, Murphy LJ (2005) Angiogenic factors are elevated in overweight and obese individuals. International journal of obesity 29: 1308-1314.
76. Garcia de la Torre N, Wass JA, Turner HE (2002) Antiangiogenic effects of somatostatin analogues. Clin Endocrinol (Oxf) 57: 425-441.
77. Kottgen A, Pattaro C, Boger CA, Fuchsberger C, Olden M, et al. (2010) New loci associated with kidney function and chronic kidney disease. Nature genetics 42: 376-384.
78. Yu Y, Bhangale TR, Fagerness J, Ripke S, Thorleifsson G, et al. (2011) Common variants near FRK/COL10A1 and VEGFA are associated with advanced age-related macular degeneration. Human molecular genetics 20: 3699-3709.
79. van Hoek M, Dehghan A, Witteman JC, van Duijn CM, Uitterlinden AG, et al. (2008) Predicting type 2 diabetes based on polymorphisms from genome-wide association studies: a population-based study. Diabetes 57: 3122-3128.
80. Boesgaard TW, Gjesing AP, Grarup N, Rutanen J, Jansson PA, et al. (2009) Variant near ADAMTS9 known to associate with type 2 diabetes is related to insulin resistance in offspring of type 2 diabetes patients--EUGENE2 study. PloS one 4: e7236.
81. Tang BL (2001) ADAMTS: a novel family of extracellular matrix proteases. Int J Biochem Cell Biol 33: 33-44.
82. Pruitt KD, Tatusova T, Maglott DR (2007) NCBI reference sequences (RefSeq): a curated non-redundant sequence database of genomes, transcripts and proteins. Nucleic acids research 35: D61-65.
83. Suzuki Y, Jiang LL, Souri M, Miyazawa S, Fukuda S, et al. (1997) D-3-hydroxyacyl-CoA dehydratase/D-3-hydroxyacyl-CoA dehydrogenase bifunctional protein deficiency: a newly identified peroxisomal disorder. American journal of human genetics 61: 1153-1162.
84. van Grunsven EG, van Berkel E, Ijlst L, Vreken P, de Klerk JB, et al. (1998) Peroxisomal D-hydroxyacyl-CoA dehydrogenase deficiency: resolution of the enzyme defect and its molecular basis in bifunctional protein deficiency. Proceedings of the National Academy of Sciences of the United States of America 95: 2128-2133.
85. True L, Coleman I, Hawley S, Huang CY, Gifford D, et al. (2006) A molecular correlate to the Gleason grading system for prostate adenocarcinoma. Proceedings of the National Academy of Sciences of the United States of America 103: 10991-10996.
86. Rasiah KK, Gardiner-Garden M, Padilla EJ, Moller G, Kench JG, et al. (2009) HSD17B4 overexpression, an independent biomarker of poor patient outcome in prostate cancer. Mol Cell Endocrinol 301: 89-96.
87. Zeggini E, Weedon MN, Lindgren CM, Frayling TM, Elliott KS, et al. (2007) Replication of genome-wide association signals in UK samples reveals risk loci for type 2 diabetes. Science 316: 1336-1341.
88. Saxena R, Voight BF, Lyssenko V, Burtt NP, de Bakker PI, et al. (2007) Genome-wide association analysis identifies loci for type 2 diabetes and triglyceride levels. Science 316: 1331-1336.

89. Ristow M, Muller-Wieland D, Pfeiffer A, Krone W, Kahn CR (1998) Obesity associated with a mutation in a genetic regulator of adipocyte differentiation. The New England journal of medicine 339: 953-959.
90. Barak Y, Nelson MC, Ong ES, Jones YZ, Ruiz-Lozano P, et al. (1999) PPAR gamma is required for placental, cardiac, and adipose tissue development. Molecular cell 4: 585-595.
91. Rosen ED, Sarraf P, Troy AE, Bradwin G, Moore K, et al. (1999) PPAR gamma is required for the differentiation of adipose tissue in vivo and in vitro. Molecular cell 4: 611-617.
92. Rankinen T, Zuberi A, Chagnon YC, Weisnagel SJ, Argyropoulos G, et al. (2006) The human obesity gene map: the 2005 update. Obesity 14: 529-644.
93. Murphy GJ, Holder JC (2000) PPAR-gamma agonists: therapeutic role in diabetes, inflammation and cancer. Trends in pharmacological sciences 21: 469-474.
94. Barroso I, Gurnell M, Crowley VE, Agostini M, Schwabe JW, et al. (1999) Dominant negative mutations in human PPARgamma associated with severe insulin resistance, diabetes mellitus and hypertension. Nature 402: 880-883.
95. Galgani A, Valdes A, Erlich HA, Mano C, Cheng S, et al. (2010) Homozygosity for the Ala allele of the PPARgamma2 Pro12Ala polymorphism is associated with reduced risk of coronary artery disease. Dis Markers 29: 259-264.
96. Kitamura S, Miyazaki Y, Shinomura Y, Kondo S, Kanayama S, et al. (1999) Peroxisome proliferator-activated receptor gamma induces growth arrest and differentiation markers of human colon cancer cells. Jpn J Cancer Res 90: 75-80.
97. Sarraf P, Mueller E, Smith WM, Wright HM, Kum JB, et al. (1999) Loss-of-function mutations in PPAR gamma associated with human colon cancer. Molecular cell 3: 799-804.
98. Xin X, Yang S, Kowalski J, Gerritsen ME (1999) Peroxisome proliferator-activated receptor gamma ligands are potent inhibitors of angiogenesis in vitro and in vivo. J Biol Chem 274: 9116-9121.
99. Morini E, Tassi V, Capponi D, Ludovico O, Dallapiccola B, et al. (2008) Interaction between PPARgamma2 variants and gender on the modulation of body weight. Obesity 16: 1467-1470.
100. Duffield JA, Vuocolo T, Tellam R, McFarlane JR, Kauter KG, et al. (2009) Intrauterine growth restriction and the sex specific programming of leptin and peroxisome proliferator-activated receptor gamma (PPARgamma) mRNA expression in visceral fat in the lamb. Pediatr Res 66: 59-65.
101. Chen CH, Lu ML, Kuo PH, Chen PY, Chiu CC, et al. (2011) Gender differences in the effects of peroxisome proliferator-activated receptor gamma2 gene polymorphisms on metabolic adversity in patients with schizophrenia or schizoaffective disorder. Prog Neuropsychopharmacol Biol Psychiatry 35: 239-245.
102. Waterworth DM, Ricketts SL, Song K, Chen L, Zhao JH, et al. (2010) Genetic variants influencing circulating lipid levels and risk of coronary artery disease. Arteriosclerosis, thrombosis, and vascular biology 30: 2264-2276.
103. Pearlman A, Loke J, Le Caignec C, White S, Chin L, et al. (2010) Mutations in MAP3K1 cause 46,XY disorders of sex development and implicate a common signal transduction pathway in human testis determination. American journal of human genetics 87: 898-904.
104. Easton DF, Pooley KA, Dunning AM, Pharoah PD, Thompson D, et al. (2007) Genome-wide association study identifies novel breast cancer susceptibility loci. Nature 447: 1087-1093.
105. Lu PH, Yang J, Li C, Wei MX, Shen W, et al. (2011) Association between mitogen-activated protein kinase kinase kinase 1 rs889312 polymorphism and breast cancer risk: evidence from 59,977 subjects. Breast Cancer Res Treat 126: 663-670.
106. Antoniou AC, Spurdle AB, Sinilnikova OM, Healey S, Pooley KA, et al. (2008) Common breast cancer-predisposition alleles are associated with breast cancer risk in BRCA1 and BRCA2 mutation carriers. American journal of human genetics 82: 937-948.

107. Sebastiani P, Solovieff N, Puca A, Hartley SW, Melista E, et al. (2010) Genetic signatures of exceptional longevity in humans. Science 2010.
108. Stahl EA, Raychaudhuri S, Remmers EF, Xie G, Eyre S, et al. (2010) Genome-wide association study meta-analysis identifies seven new rheumatoid arthritis risk loci. Nature genetics 42: 508-514.
109. Zhernakova A, Stahl EA, Trynka G, Raychaudhuri S, Festen EA, et al. (2011) Meta-analysis of genome-wide association studies in celiac disease and rheumatoid arthritis identifies fourteen non-HLA shared loci. PLoS genetics 7: e1002004.
110. Emilsson V, Thorleifsson G, Zhang B, Leonardson AS, Zink F, et al. (2008) Genetics of gene expression and its effect on disease. Nature 452: 423-428.
111. Dixon AL, Liang L, Moffatt MF, Chen W, Heath S, et al. (2007) A genome-wide association study of global gene expression. Nature genetics 39: 1202-1207.
112. Stranger BE, Nica AC, Forrest MS, Dimas A, Bird CP, et al. (2007) Population genomics of human gene expression. Nature genetics 39: 1217-1224.
113. Bauer S, Wanninger J, Schmidhofer S, Weigert J, Neumeier M, et al. (2011) Sterol regulatory element-binding protein 2 (SREBP2) activation after excess triglyceride storage induces chemerin in hypertrophic adipocytes. Endocrinology 152: 26-35.
114. Demarest K, Koyner J, McCaughran J, Cipp L, Hitzemann R (2001) Further characterization and high-resolution mapping of quantitative trait loci for ethanol-induced locomotor activity. Behavior genetics 31: 79-91.
115. Valdar W, Solberg LC, Gauguier D, Burnett S, Klenerman P, et al. (2006) Genome-wide genetic association of complex traits in heterogeneous stock mice. Nature genetics 38: 879-887.
116. Huang GJ, Shifman S, Valdar W, Johannesson M, Yalcin B, et al. (2009) High resolution mapping of expression QTLs in heterogeneous stock mice in multiple tissues. Genome research 19: 1133-1140.
117. Team R (2010) R: A language and environment for statistical computing. R Foundation for Statistical Computing Vienna Austria.
118. Du P, Kibbe WA, Lin SM (2008) lumi: a pipeline for processing Illumina microarray. Bioinformatics 24: 1547-1548.
119. Barbosa-Morais NL, Dunning MJ, Samarajiwa SA, Darot JFJ, Ritchie ME, et al. (2010) A re-annotation pipeline for Illumina BeadArrays: improving the interpretation of gene expression data. Nucleic acids research 38: e17-e17.
120. Li C, Rabinovic A (2007) Adjusting batch effects in microarray expression data using empirical Bayes methods. Biostatistics 8: 118-127.
