# Supplementary material for: Sex-stratified Genome-wide Association Studies Including 270,000 Individuals Show Sexual Dimorphism in Genetic Loci for Anthropometric Traits
Source: PLoS Genet. 2013 Jun 6;9(6):e1003500. doi: 10.1371/journal.pgen.1003500 (PMC3674993; doi:10.1371/journal.pgen.1003500)
Supplement: Text S2 — Extended acknowledgments. (PDF) [file pgen.1003500.s020.pdf]

Text S2: Extended Acknowledgements

Cohort	Acknowledgements (funding, personal, groups, …)
**Discovery Stage**	
ADVANCE	The ADVANCE study was supported by a grant from the Reynold's Foundation and NHLBI grant HL087647.
AGES Midlife	The Age, Gene/Environment Susceptibility Reykjavik Study has been funded by NIH contract N01-AG-12100, the NIA Intramural Research Program, Hjartavernd (the Icelandic Heart Association), and the Althingi (the Icelandic Parliament). The study is approved by the Icelandic National Bioethics Committee, (VSN: 00-063) and the Data Protection Authority. The researchers are indebted to the participants for their willingness to participate in the study.
Amish	We gratefully acknowledge our Amish liaisons, field workers and clinic staff and the extraordinary cooperation and support of the Amish community without which these studies would not have been possible. The Amish studies are supported by grants and contracts from the NIH, including U01 HL072515, U01 HL84756, the University of Maryland General Clinical Research Center, grant M01 RR 16500, the Mid-Atlantic Nutrition and Obesity research Center, grant NIH P30 DK072488, and by National Research Initiative Competitive Grant no. 2007-35205-17883 from the USDA National Institute of Food and Agriculture. We thank our Amish research volunteers for their long-standing partnership in research, and the research staff at the Amish Research Clinic for their hard work and dedication.
ARIC	The Atherosclerosis Risk in Communities Study is carried out as a collaborative study supported by National Heart, Lung, and Blood Institute contracts (HHSN268201100005C, HHSN268201100006C, HHSN268201100007C, HHSN268201100008C, HHSN268201100009C, HHSN268201100010C, HHSN268201100011C, and HHSN268201100012C), R01HL087641, R01HL59367 and R01HL086694; National Human Genome Research Institute contract U01HG004402; and National Institutes of Health contract HHSN268200625226C. The authors thank the staff and participants of the ARIC study for their important contributions. Infrastructure was partly supported by Grant Number UL1RR025005, a component of the National Institutes of Health and NIH Roadmap for Medical Research.
B58C-WTCCC and B58C-T1DGC	We acknowledge use of phenotype and genotype data from the British 1958 Birth Cohort DNA collection, funded by the Medical Research Council grant G0000934 and the Wellcome Trust grant 068545/Z/02. (http://www.b58cgene.sgul.ac.uk/). Genotyping for the B58C-WTCCC subset was funded by the Wellcome Trust grant 076113/B/04/Z. The B58C-T1DGC genotyping utilized resources provided by the Type 1 Diabetes Genetics Consortium, a collaborative clinical study sponsored by the National Institute of Diabetes and Digestive and Kidney Diseases (NIDDK), National Institute of Allergy and Infectious Diseases (NIAID), National Human Genome Research Institute (NHGRI), National Institute of Child Health and Human Development (NICHD), and Juvenile Diabetes Research Foundation International (JDRF) and supported by U01 DK062418. B58C-T1DGC GWAS data were deposited by the Diabetes and Inflammation Laboratory, Cambridge Institute for Medical Research (CIMR), University of Cambridge, which is funded by Juvenile Diabetes Research Foundation International, the Wellcome Trust and the National Institute for Health Research Cambridge Biomedical Research Centre; the CIMR is in receipt of a Wellcome Trust Strategic Award (079895).

Cohort	Acknowledgements (funding, personal, groups, …)
BRIGHT	The BRIGHT study is supported by the Medical Research Council of Great Britain (G9521010D) and the British Heart Foundation (PG/02/128). The BRIGHT study is extremely grateful to all the patients who participated in the study and the BRIGHT nursing team. We also acknowledge the contribution of Chris Wallace for the analysis. This work forms part of the research themes contributing to the translational research portfolio of Barts and the London Cardiovascular Biomedical Research Unit which is supported and funded by the National Institute of Health Research.
CAD_WTCCC	CAD-WTCCC (WTCCC Coronary Artery Disease cases) - Collection of the CAD-WTCCC cases (BHF Family Heart Study) was funded by the British Heart Foundation and the Medical Research Council and genotyping by the Wellcome Trust as part of the WTCCC. We thank the members of the BHF Family Heart Study Research Group for recruitment. N.J.S. holds a personal chair supported by the BHF and also holds a UK NIHR Senior Investigator Award.
CAPS	The CAPS study was supported by grants from the Swedish Research Council, the Swedish Cancer Society, and the National Cancer Institute. E.I. was supported by grants from the Swedish Research Council, the Swedish Heart - Lung Foundation, the Swedish Society of Medicine, the Swedish Foundation for Strategic Research, and the Royal Swedish Academy of Science while working with this article.
CHS	This CHS research was supported by NHLBI contracts N01-HC-85239, N01-HC-85079 through N01-HC-85086; N01-HC-35129, N01 HC-15103, N01 HC-55222, N01-HC-75150, N01-HC-45133 and NHLBI grants HL080295, HL075366, HL087652, HL105756 with additional contribution from NINDS. Additional support was provided through AG-023629, AG-15928, AG-20098, and AG-027058 from the NIA. See also http://www.chs-nhlbi.org/pi.htm. DNA handling and genotyping was supported in part by National Center for Research Resources CTSI grant UL 1RR033176, National Institute of Diabetes and Digestive and Kidney Diseases grant DK063491 to the Southern California Diabetes Endocrinology Research Center.
COLAUS	The CoLaus study received financial contributions from GlaxoSmithKline, the Faculty of Biology and Medicine of Lausanne, and the Swiss National Science Foundation (33CSCO-122661). The authors thank Vincent Mooser, Dawn Waterworth, Peter Vollenweider and Gérard Waeber, Co-PIs of the CoLaus study. Special thanks to Murielle Bochud, Yolande Barreau, Mathieu Firmann, Vladimir Mayor, Anne-Lise Bastian, Binasa Ramic, Martine Moranville, Martine Baumer, Marcy Sagette, Jeanne Ecoffey and Sylvie Mermoud for data collection. JSB is supported by the Centre Hospitalier Universitaire Vaudois and the University of Lausanne, Switzerland, the Swiss National Science Foundation (grants nb 310000-112552) and the European Union HEALTH-F4-2007-201550 HYPERGENES grant.
deCODE	We would like to thank participants in deCODE cardiovascular- and obesity studies and collaborators for their cooperation. The research performed at deCODE Genetics was part funded through the European Community's Seventh Framework Programme (FP7/2007-2013), ENGAGE project, grant agreement HEALTH-F4-2007- 201413.

Cohort	Acknowledgements (funding, personal, groups, …)
DGI	The Botnia (DGI) study have been supported by grants from Folkhälsan Research Foundation, Sigrid Juselius Foundation, Ministry of Education, Nordic Center of Excellence in Disease Genetics, Gyllenberg Foundation, Swedish Cultural Foundation in Finland, Finnish Diabetes Research Foundation, Foundation for Life and Health in Finland, Finnish Medical Society, Paavo Nurmi Foundation, Perklén Foundation, Ollqvist Foundation, Närpes Health Care Foundation, the Municipal Health Care Center and Hospital in Jakobstad, Health Care Centers in Vasa, Närpes and Korsholm. This work was also partially supported by NIH grant R01-DK075787 to JNH and March of Dimes grant 6-FY-09-507 to JNH.
EGCUT	EGCUT received financing by FP7 grants (201413, 245536), also received targeted financing from Estonian Government SF0180142s08 and from the European Union through the European Regional Development Fund, in the frame of Centre of Excellence in Genomics and Estoinian Research Infrastructure's Roadmap. We acknowledge EGCUT and Estonian Biocentre personnel, especially Ms. M. Hass and Mr V. Soo
EPIC	The EPIC Norfolk Study is funded by Cancer Research United Kingdom and the Medical Research Council.
ERF (EUROSPAN)	The genotyping for the ERF study was supported by EUROSPAN (European Special Populations Research Network) and the European Commission FP6 STRP grant (018947; LSHG-CT-2006-01947). The ERF study was further supported by grants from the Netherlands Organisation for Scientific Research, Erasmus MC, the Centre for Medical Systems Biology (CMSB) and the Netherlands Brain Foundation (HersenStichting Nederland). We are grateful to all patients and their relatives, general practitioners and neurologists for their contributions and to P. Veraart for her help in genealogy, Jeannette Vergeer for the supervision of the laboratory work and P. Snijders for his help in data collection. We would also like to acknowledge Internationale Stichting Alzheimer Onderzoek (ISAO) and Hersenstichting Netherlands.
FamHS	The Family Heart Study (FamHS) was supported by NIH grants R01-HL-087700 and R01-HL-088215 (Michael A. Province, PI) from NHLBI; and R01-DK-8925601 and R01-DK-075681 (Ingrid B. Borecki, PI) from NIDDK.
FENLAND	The Fenland Study is funded by the Wellcome Trust and the Medical Research Council, as well as by the Support for Science Funding programme and CamStrad. We are grateful to all the volunteers for their time and help, and to the General Practitioners and practice staff for help with recruitment. We thank the Fenland Study co-ordination team and the Field Epidemiology team of the MRC Epidemiology Unit for recruitment and clinical testing.
FRAM	This research was conducted in part using data and resources from the Framingham Heart Study of the National Heart Lung and Blood Institute of the National Institutes of Health and Boston University School of Medicine. The analyses reflect intellectual input and resource development from the Framingham Heart Study investigators participating in the SNP Health Association Resource (SHARe) project. This work was partially supported by the National Heart, Lung and Blood Institute's Framingham Heart Study (Contract No. N01-HC-25195) and its contract with Affymetrix, Inc for genotyping services (Contract No. N02-HL-6-4278). A portion of this research utilized the Linux Cluster for Genetic Analysis (LinGA-II) funded by the Robert Dawson Evans Endowment of the Department of Medicine at Boston University School of Medicine and Boston Medical Center.

Cohort	Acknowledgements (funding, personal, groups, …)
FTC	Finnish Twin cohort is supported by the European Commission under the programme 'Quality of Life and Management of the Living Resources' of 5th Framework Programme (no. QLG2-CT-2002-01254). S.R was supported by the Academy of Finland Center of Excellence in Complex Disease Genetics (213506 and 129680), Academy of Finland (251217), the Finnish foundation for Cardiovascular Research and the Sigrid Juselius Foundation. J.K. has been supported by the Academy of Finland Centre of Excellence in Complex Disease Genetics.
Genmets	Genmets was supported through funds from The European Community's Seventh Framework Programme (FP7/2007-2013), BioSHaRE Consortium, grant agreement 261433. V.S. was supported by the Sigrid Juselius Foundation, Finnish Foundation for Cardiovascular research, and the Finnish Academy (grant number 129494).
GERMIFS1 and GERMIFS2	GerMIFS1, GerMIFS2: Supported by the Deutsche Forschungsgemeinschaft and the German Federal Ministry of Education and Research (BMBF) in the context of the German National Genome Research Network (NGFN-2 and NGFN-plus), the FP6 and FP7 EU funded integrated projects Cardiogenics (LSHM-CT-2006-037593), ENGAGE (201413), the bi-national BMBF/ANR funded project CARDomics (01KU0908A), and the Nordic Center of Cardiovascular Research (NCCR).
KORA	The MONICA/KORA Augsburg studies were financed by the Helmholtz Zentrum München, German Research Center for Environmental Health, Neuherberg, Germany, and supported by grants from the German Federal Ministry of Education and Research (BMBF). Part of this work was financed by the German National Genome Research Network (NGFNPlus, project number 01GS0834, 01GS0823), software development for sex-specific GWAMAS was partly funded by BMBF 01ER1206; additional funds were from the University of Ulm. Furthermore, the research was supported within the Munich Center of Health Sciences (MC Health) as part of LMU innovative.
MICROS	For the MICROS study, we thank the primary care practitioners in the villages of the Val Venosta and the personnel of the Hospital of Silandro (Department of Laboratory Medicine) for their participation and collaboration in the research project. In South Tyrol, the study was supported by the Ministry of Health and Department of Educational Assistance, University and Research of the Autonomous Province of Bolzano, and the South Tyrolean Sparkasse Foundation.
Migen	The MIGen study was supported by the National Heart, Lung, and Blood Institute's STAMPEED genomics research program (R01 HL087676) and the National Center for Research Resources (U54 RR020278). This work was also partially supported by NIH grant K23-DK080145 to EKS, NIH grant R01-DK075787 to JNH and March of Dimes grant 6-FY-09-507 to JNH.
NBS-WTCCC	We acknowledge use of DNA from The UK Blood Services collection of Common Controls (UKBS-CC collection), funded by the Wellcome Trust grant 084183/Z/07/Z and by NIHR programme grant to NHSBT (RP-PG-0310-1002). The collection was established as part of the Wellcome Trust Case Control Consortium (WTCCC)

Cohort	Acknowledgements (funding, personal, groups, …)
NFBC1966	NFBC1966 received financial support from the Academy of Finland (project grants 104781, 120315, 129269, 1114194, Center of Excellence in Complex Disease Genetics and SALVE), University Hospital Oulu, Biocenter, University of Oulu, Finland (75617), the European Commission (EURO-BLCS, Framework 5 award QLG1-CT-2000-01643), NHLBI grant 5R01HL087679-02 through the STAMPEED program (1RL1MH083268-01), NIH/NIMH (5R01MH63706:02), ENGAGE project and grant agreement HEALTH-F4-2007-201413, and the Medical Research Council, UK (G0500539, G0600705, PrevMetSyn/SALVE).<br><br>The DNA extractions, sample quality controls, biobank up-keeping and aliquotting was performed in the National Public Health Institute, Biomedicum Helsinki, Finland and supported financially by the Academy of Finland and Biocentrum Helsinki. We thank Professor (emeriti) Paula Rantakallio (launch of NFBC1966 and 1986), and Ms Outi Tornwall and Ms Minttu Jussila (DNA biobanking). The authors would like to acknowledge the contribution of the late Academian of Science Leena Peltonen.
NHS	This study was supported by grants HL71981, CA65725, CA87969, CA49449, CA67262, CA50385 and 5UO1CA098233 from the National Institutes of Health. Dr. Lu Qi is a recipient of the American Heart Association Scientist Development Award (0730094N)
NSPHS	The Northern Swedish Population Health Study (NSPHS) was funded by the Swedish Medical Research Council (project number K2007-66X-20270-01-3), and the Foundation for Strategic Research (SSF). The NSPHS as part of EUROSPAN (European Special Populations Research Network) was also supported by European Commission FP6 STRP grant number 01947 (LSHG-CT-2006-01947). This work was also supported by the Swedish Society for Medical Research (ÅJ). The authors are grateful for the contribution of district nurse Svea Hennix for data collection and Inger Jonasson for logistics and coordination of the health survey. Finally, the authors thank all the community participants for their interest and willingness to contribute to the study.
NTRNESDA	Funding was obtained from the Netherlands Organization for Scientific Research (NWO: MagW/ZonMW): Genetic basis of anxiety and depression (904-61-090); Genetics of individual differences in smoking initiation and persistence (NWO 985-10-002); Resolving cause and effect in the association between exercise and well-being (904-61-193); Twin family database for behavior genomics studies (480-04-004); Twin research focusing on behavior (400-05-717); Genetic determinants of risk behavior in relation to alcohol use and alcohol use disorder (Addiction-31160008); Genotype/phenotype database for behavior genetic and genetic epidemiological studies (40-0056-98-9032); Spinozapremie (SPI 56-464-14192); CMSB: Center for Medical Systems Biology (NWO Genomics); NBIC/BioAssist/RK/2008.024); BBMRI –NL: Biobanking and Biomolecular Resources Research Infrastructure (184.021.007); the VU University: Institute for Health and Care Research (EMGO+) and Neuroscience Campus Amsterdam (NCA); the European Science Foundation (ESF): Genomewide analyses of European twin and population cohorts (EU/QLRT-2001-01254); European Community's Seventh Framework Program (FP7/2007-2013): ENGAGE (HEALTH-F4-2007-201413); the European Science Council (ERC) Genetics of Mental Illness (230374); Rutgers University Cell and DNA Repository cooperative agreement (NIMH U24 MH068457-06); Collaborative study of the genetics of DZ twinning (NIH R01D0042157-01A); the Genetic Association Information Network, a public–private partnership between the NIH and Pfizer Inc., Affymetrix Inc. and Abbott Laboratories.

Cohort	Acknowledgements (funding, personal, groups, …)
ORCADES	ORCADES was supported by the Chief Scientist Office of the Scottish Government, the Royal Society, and the European Union framework program 6 European Special Populations Research Network project [contract LSHG-CT-2006-018947]. ORCADES would like to acknowledge the invaluable contributions of Lorraine Anderson, the research nurses in Orkney, and the administrative team in Edinburgh. DNA extraction for ORCADES was carried out by the Genetics Core Laboratory at the Wellcome Trust Clinical Research Facility, WGH, Edinburgh, Scotland and SNP genotyping was performed by Helmholtz Zentrum München, GmbH, Neuherberg, Germany.
PLCO	The Prostate, Lung, Colorectal, and Ovarian (PLCO) Cancer Screening Trial was funded by the Intramural Research Program of the Division of Cancer Epidemiology and Genetics, National Cancer Institute, NIH
PROCARDIS	European Commission (LSHM-CT-2007-037273), the Swedish Heart-Lung Foundation, the Swedish Research Council (8691), the Knut and Alice Wallenberg Foundation, the Foundation for Strategic Research, the Torsten and Ragnar Söderberg Foundation, the Strategic Cardiovascular Programme of Karolinska Institutet and the Stockholm County Council, the Stockholm County Council (560183) and the Wellcome trust core award [090532/Z/09/Z]. MF acknowledges support from the British Heart Foundation Centre of Research Excellence, Oxford.
RS1	The generation and management of GWAS genotype data for the Rotterdam Study is supported by the Netherlands Organisation of Scientific Research NWO Investments (nr. 175.010.2005.011, 911-03-012). This study is funded by the Research Institute for Diseases in the Elderly (014-93-015; RIDE2), the Netherlands Genomics Initiative (NGI)/Netherlands Organisation for Scientific Research (NWO) project nr. 050-060-810. We thank Pascal Arp, Mila Jhamai, Marijn Verkerk, Lizbeth Herrera and Marjolein Peters for their help in creating the GWAS database, and Karol Estrada and Maksim V. Struchalin for their support in creation and analysis of imputed data. The Rotterdam Study is funded by Erasmus Medical Center and Erasmus University, Rotterdam, Netherlands Organization for the Health Research and Development (ZonMw), the Research Institute for Diseases in the Elderly (RIDE), the Ministry of Education, Culture and Science, the Ministry for Health, Welfare and Sports, the European Commission (DG XII), and the Municipality of Rotterdam. The authors are grateful to the study participants, the staff from the Rotterdam Study and the participating general practitioners and pharmacists.
RunMC	The RUNMC Nijmegen Biomedical Study was made possible by research investment grants of the Radboud University Nijmegen Medical Centre and the Municipality of Nijmegen.
Sardinia	We thank all the volunteers who generously participated in this study, Monsignore Piseddu, Bishop of Ogliastra and the mayors and citizens of the Sardinian towns (Lanusei, Ilbono, Arzana, and Elini). This work was supported by the Intramural Research Program of the National Institute on Aging (NIA), National Institutes of Health (NIH). The SardiNIA ("Progenia") team was supported by Contract NO1-AG-1–2109 from the NIA; the efforts of GRA were supported in part by contract 263-MA-410953 from the NIA to the University of Michigan and by research grant HG002651 and HL084729 from the NIH (to GRA).
SASBAC	The SASBAC study was supported by funding from the Agency for Science, Technology and Research of Singapore (A*STAR), the United States National Institute of Health (NIH) and the Susan G. Komen Breast Cancer Foundation.
SEARCH	Paul Pharoah, Douglas Easton, Simon Gayther and the SEARCH and UKOPS teams. SEARCH was funded by Cancer Research UK (C490/A10124 and C490/A8339).

Cohort	Acknowledgements (funding, personal, groups, …)
SHIP	SHIP is part of the Community Medicine Research net of the University of Greifswald, Germany, which is funded by the Federal Ministry of Education and Research (grants no. 01ZZ9603, 01ZZ0103, and 01ZZ0403), the Ministry of Cultural Affairs as well as the Social Ministry of the Federal State of Mecklenburg-West Pomerania, and the network 'Greifswald Approach to Individualized Medicine (GANI_MED)' funded by the Federal Ministry of Education and Research (grant 03IS2061A). Genome-wide data have been supported by the Federal Ministry of Education and Research (grant no. 03ZIK012) and a joint grant from Siemens Healthcare, Erlangen, Germany and the Federal State of Mecklenburg- West Pomerania. The University of Greifswald is a member of the 'Center of Knowledge Interchange' program of the Siemens AG and the Caché Campus program of the InterSystems GmbH.
T2D-WTCCC	Research funding for sample collection, genotyping and data analysis for the T2D-WTCCC and other cohorts for which the Oxford group<br>had responsibility came from the British Diabetes Association, BDA Research, Diabetes UK, Oxford NIHR Biomedical Research Centre, European Commission (ENGAGE: HEALTH-F4-2007-201413;<br>EURODIA: LSHG-CT-2004-518153, Wellcome Trust (072960, 076113/B/04/Z, 076113/K/04/Z, 083270, 085301, 079557, 081682, 075491) UK Medical Research Council (G0000649, G0601261)<br>and NIDDK (R01-DK-073490). In addition, Cecilia Lindgren is funded by WT086596/Z/08/Z (Wellcome Trust Research Career Development Fellowship); Reedik Mägi is funded by European Commission under the Marie Curie Intra-European Fellowship; and Mark McCarthy receivespersonal funding from the Oxford NIHR Biomedical Research Centre.
TwinsUK	The study was funded by the Wellcome Trust; European Community's Seventh Framework Programme (FP7/2007-2013)/grant agreement HEALTH-F2-2008-ENGAGE and the European Union FP-5 GenomEUtwin Project (QLG2-CT-2002-01254) and Framework 6 Project EUroClot. The study also receives support from the National Institute for Health Research (NIHR) comprehensive Biomedical Research Centre award to Guy's & St Thomas' NHS Foundation Trust in partnership with King's College London We thank the staff from the TwinsUK, the DNA Collections and Genotyping Facilities at the Wellcome Trust Sanger Institute for sample preparation; Quality Control of the Twins UK cohort for genotyping (in particular Amy Chaney, Radhi Ravindrarajah, Douglas Simpkin, Cliff Hinds, and Thomas Dibling); Paul Martin and Simon Potter of the DNA and Genotyping Informatics teams for data handling; Le Centre National de Génotypage, France, led by Mark Lathrop, for genotyping; Duke University, North Carolina, USA, led by David Goldstein, for genotyping; and the Finnish Institute of Molecular Medicine, Finnish Genome Center, University of Helsinki, led by Aarno Palotie.
VIS	The CROATIA-Vis study was funded by grants from the Medical Research Council (UK), European Commission Framework 6 project EUROSPAN (Contract No. LSHG-CT-2006-018947) and Republic of Croatia Ministry of Science, Education and Sports research grants. (108-1080315-0302). The funders had no role in study design, data collection and analysis, decision to publish, or preparation of the manuscript."<br>We would like to acknowledge the staff of several institutions in Croatia that supported the field work, including but not limited to The University of Split and Zagreb Medical Schools, Institute for Anthropological Research in Zagreb and Croatian Institute for Public Health. The SNP genotyping for the CROATIA-Vis cohort was performed in the core genotyping laboratory of the Wellcome Trust Clinical Research Facility at the Western General Hospital, Edinburgh, Scotland, UK.

Follow-Up Stage - Insilico	
**Cohort**	**Acknowledgements (funding, personal, groups, …)**
BHS	The Busselton Health Study acknowledges the generous support for the 1994/5 follow-up study from Healthway, Western Australia and the numerous Busselton community volunteers who assisted with data collection and the study participants from the Shire of Busselton. The Busselton Health Study is supported by The Great Wine Estates of the Margaret River region of Western Australia. The BHS gratefully acknowledges the assistance of the Western Australian DNA Bank (NHMRC Enabling Facility) with DNA samples and the support provided by the Ark (NHMRC Enabling Facility) for this study.
COROGENE	The study was supported in part by the Aarno Koskelo Foundation, the Finnish Foundation for Cardiovascular Research, and the EVO funds of Helsinki University Central Hospital. The Corogene study is not supported by industry and all presentations have been carried out in academic environment.
FinGesture	FinGesture (Finnish Genetic Study of Arrhythmic Events) - We thank the study participants. We also thank Kari Kaikkonen and Marja-Leena Kortelainen for study concept and design, and data acquisition and interpretation. The FinGesture cohort is supported by the Juselius Foundation (Helsinki, Finland) and the Council of Health of the Academy of Finland (Helsinki, Finland). Authors would like to thank Guillaume Lettre, Jean-Claude Tardif, Juhani Juntila, Philipe Goyette and Sylvain Foisy for their contributions to the design, implementation and analysis of the GWA study of the FinGesture cohort. In addition, we would like to acknowledge the support of the Montreal Heart Institute Foundation.
GOOD	Financial support was received from the Swedish Research Council (K2010-54X-09894-19-3, 2006-3832 and K2010-52X-20229-05-3), the Swedish Foundation for Strategic Research, the ALF/LUA research grant in Gothenburg, the Lundberg Foundation, the Torsten and Ragnar Söderberg's Foundation, Petrus and Augusta Hedlunds Foundation, the Västra Götaland Foundation, the Göteborg Medical Society, the Novo Nordisk foundation, and the European Commission grant HEALTH-F2-2008-201865-GEFOS. We would like to acknowledge Maria Nethander at the Genomics core facility at the University of Gothenburg for statistical analyses. We would like to thank Dr. Tobias A. Knoch, Luc V. de Zeeuw, Anis Abuseiris, and Rob de Graaf as well as their institutions the Erasmus Computing Grid, Rotterdam, The Netherlands, and especially the national German MediGRID and Services@MediGRID part of the German D-Grid, both funded by the German Bundesministerium fuer Forschung und Technology under grants #01 AK 803 A-H and # 01 IG 07015 G for access to their grid resources. We would also like to thank Karol Estrada, Department of Internal Medicine, Erasmus MC, Rotterdam, Netherlands for advice regarding the grid resources.
HBCS	Helsinki Birth Cohort Study has been supported by grants from the Academy of Finland (Grant No. 120386 and 125876 to JGE), the Finnish Diabetes Research Society, Folkhälsan Research Foundation, Novo Nordisk Foundation, Finska Läkaresällskapet, the European Science Foundation (EuroSTRESS), the Wellcome Trust (Grant No. 89061/Z/09/Z and 089062/Z/09/Z), Samfundet Folkhälsan, Finska Läkaresällskapet and the Signe and Ane Gyllenberg foundation. We thank all study participants as well as everybody involved in the Helsinki Birth Cohort Study.

Cohort	Acknowledgements (funding, personal, groups, …)
HYPERGENES	funding: HYPERGENES (FP7 - HEALTH-F4-2007-201550); INTEROMICS (MIUR - CNR Italian Flagship Project).<br>To HYPERGENES consortium took part:<br>1) University of Milano and Fondazione Filarete with Daniele Cusi, Project Coordinator, Cristina Barlassina, Erika Salvi, Sara Lupoli, Maurizio Marconi, Gianna Petrini, Vincenzo Toschi,.<br>2) Katholieke Universiteit Leuven, with Jan Staessen, Jan Staessen, Tatiana Kuznetsova, Lutgarde Thijs.<br>3) Jagiellonian University Medical College, Krakow, with Kalina Kawecka-Jaszcz, Katarzyna Stolarz, Agnieszka Olszanecka, Wiktoria Wojciechowska.<br>4) IBM Israel – Science and Technology LTD, with Amnon Shabo, Ariel Frakash, Simona Cohen, Boaz Carmeli, Dan Pelleg, Michal Rosen-Zvi, Hani Neuvrith-Telem.<br>5) I.M.S. – Istituto di Management Sanitario S.r.l., Milan, with Pietro Conti, Costanza Conti, Mariella D'Alessio.<br>6) Institute of Internal Medicine, Siberian Branch of Russian Academy of Medical Sciences, Novosibirsk, with Yuri Nikitin, Sofia Malyutina, M. Voevoda, Andrew Ryabikov, E. Pello, Maxim Ryabikov.<br>7) Imperial College of Science, Technology and Medicine, with Paolo Vineis and Clive J Hoggart.<br>8) INSERM – Institut National de la Santé et de la Recherche Médicale U772, with Xavier Jeunemaitre, Pierre-François Plouin, Anne-Paule Gimenez-Roqueplo, Rosa Vargas-Poussou, Geneviève Beaurain.<br>9) University of Warwick. Cardiovascular Medicine & Epidemiology Group, Clinical Sciences Research Institute, with Francesco P Cappuccio, Michelle A Miller, Chen Ji<br>10) Università degli Studi di Sassari. Hypertension and cardiovascular prevention centre, with Nicola Glorioso, Chiara Maria Troffa, Giuseppe Argiolas, Francesca Fau, Silvia Pitzoi.<br>11) STMICROELECTRONICS SRL, with Enrico Rosario Alessi.<br>12) Universite de Lausanne. Department of Medical Genetics, with Carlo Rivolta, Jacques S. Beckmann, Zoltan Kutalik, Paola Benaglio.<br>13) Pharnext S.A.S., Paris, with Daniel Cohen and Ilya Chumakov.<br>14) Softeco Sismat Spa, Genova, with Stefano Bianchi.<br>15) Shanghai Institute of Hypertension, with Jiguang Wang and Li Yan.<br>16) Charles University in Prague. Department of Internal Medicine II, Pilsen, with Jan Filipovsky, Otto Mayer, Milan Hromadka, Jitka Seidlerova, Milena Dolejsova, Lukas Handl.<br>17) Università degli Studi di Padova. Department of Clinical and Experimental Medicine, with Edoardo Casiglia, Valerie Tikhonoff, Laura Schiavon, Anna Bascelli, Elisa Pagnin.<br>18) Medical University of Gdansk. Hypertension Unit, Department of Hypertension and Diabetology, with Krzysztof Narkiewicz, Marzena Chrostowska, Radoslaw Szczech, Michal Hoffmann.<br>19) University Vita-Salute San Raffaele, with Paolo Manunta, Chiara Lanzani, Maria Teresa Sciarrone, Lorena Citterio, Laura Zagato.

Cohort	Acknowledgements (funding, personal, groups, …)
LifeLines Cohort Study	The LifeLines Cohort Study, and generation and management of GWAS genotype data for the LifeLines Cohort Study is supported by the Netherlands Organization of Scientific Research NWO (grant 175.010.2007.006), the Economic Structure Enhancing Fund (FES) of the Dutch government, the Ministry of Economic Affairs, the Ministry of Education, Culture and Science, the Ministry for Health, Welfare and Sports, the Northern Netherlands Collaboration of Provinces (SNN), the Province of Groningen, University Medical Center Groningen, the University of Groningen, Dutch Kidney Foundation and Dutch Diabetes Research Foundation. We thank Behrooz Alizadeh, Annemieke Boesjes, Marcel Bruinenberg, Noortje Festen, Ilja Nolte, Lude Franke, Mitra Valimohammadi for their help in creating the GWAS database, and Rob Bieringa, Joost Keers, René Oostergo, Rosalie Visser, Judith Vonk for their work related to data-collection and validation. The authors are grateful to the study participants, the staff from the LifeLines Cohort Study and Medical Biobank Northern Netherlands, and the participating general practitioners and pharmacists. Statistical analyses were carried out on the Genetic Cluster Computer (http://www.geneticcluster.org) which is financially supported by the Netherlands Scientific Organization (NWO 480-05-003) along with a supplement from the Dutch Brain Foundation and the VU University Amsterdam.
MGS	The Molecular Genetics of Schizophrenia project was carried out by 10 research sites and PIs: Pablo V. Gejman, Study Coordinator (Department of Psychiatry and Behavioral Sciences, NorthShore University HealthSystem, Evanston, IL, and Department of Psychiatry and Behavioral Sciences, University of Chicago, Chicago, IL), Douglas F. Levinson (Stanford University), Bryan J. Mowry (University of Queensland), Donald Black (University of Iowa), Robert Freedman (University of Colorado), C. Robert Cloninger (Washington University), Jeremy Silverman (Mt. Sinai Medical School), Nancy Buccola (Louisiana State University - New Orleans), William Byerley (University of California at San Francisco), and Farooq Amin (Emory University).<br>This study was supported by NIH R01 grants (MH67257 to N.G.B., MH59588 to B.J.M., MH59571 to P.V.G., MH59565 to R.F., MH59587 to F.A., MH60870 to W.F.B., MH59566 to D.W.B., MH59586 to J.M.S., MH61675 to D.F.L., MH60879 to C.R.C., and MH81800 to P.V.G.), MH79469 to P.V.G., and MH79470 to D.F.L.), NARSAD (National Alliance for Research on Schizophrenia and Depression) Young Investigator Awards (to J.D. and A.R.S.), the Genetic Association Information Network (GAIN), the Walter E. Nichols, M.D., and Eleanor Nichols endowments, at Stanford University, and by The Paul Michael Donovan Charitable Foundation. Genotyping was carried out by the Genotyping and Analysis at the Broad Institute of Harvard and MIT (S. Gabriel and D.B.M.), which is supported by grant U54 RR020278 from the National Center for Research Resources. Genotyping of half of the control sample presented here was carried out with support from GAIN. The GAIN quality control team (G.R. Abecasis and J. Paschall) made important contributions to the project.
PLCO2	The Prostate, Lung, Colorectal, and Ovarian (PLCO) Cancer Screening Trial was funded by the Intramural Research Program of the Division of Cancer Epidemiology and Genetics, National Cancer Institute, NIH
PREVEND	PREVEND genetics is supported by the Dutch Kidney Foundation (Grant E033), the EU project grant GENECURE (FP-6 LSHM CT 2006 037697), the National Institutes of Health (grant LM010098), The Netherlands organisation for health research and development (NWO VENI grant 916.761.70), and the Dutch Inter University Cardiology Institute Netherlands (ICIN).

Cohort	Acknowledgements (funding, personal, groups, …)
QIMR	We are grateful to the twins and their families for their generous participation in these studies. We would like to thank staff at the Queensland Institute of Medical Research: Dixie Statham, Ann Eldridge and Marlene Grace for sample collection, Anjali Henders, Megan Campbell, Lisa Bowdler, Steven Crooks and staff of the Molecular Epidemiology Laboratory for sample processing and preparation, Scott Gordon, Brian McEvoy, Belinda Cornes and Beben Benyamin for data QC and preparation, and David Smyth and Harry Beeby for IT support. We acknowledge funding from the Australian National Health and Medical Research Council (NHMRC grants 241944, 389875, 389891, 389892, 389938, 442915, 442981, 496739, 496688, 552485, 613672, 613601 and 1011506), the U.S. National Institute of Health (grants AA07535, AA10248, AA014041, AA13320, AA13321, AA13326 and DA12854) and the Australian Research Council (ARC grants DP0770096 and DP1093900).
RS2, RS3	The generation and management of GWAS genotype data for the Rotterdam Study is supported by the Netherlands Organisation of Scientific Research NWO Investments (nr. 175.010.2005.011, 911-03-012). This study is funded by the Research Institute for Diseases in the Elderly (014-93-015; RIDE2), the Netherlands Genomics Initiative (NGI)/Netherlands Organisation for Scientific Research (NWO) project nr. 050-060-810. We thank Pascal Arp, Mila Jhamai, Marijn Verkerk, Lizbeth Herrera and Marjolein Peters for their help in creating the GWAS database, and Karol Estrada and Maksim V. Struchalin for their support in creation and analysis of imputed data.<br>The Rotterdam Study is funded by Erasmus Medical Center and Erasmus University, Rotterdam, Netherlands Organization for the Health Research and Development (ZonMw), the Research Institute for Diseases in the Elderly (RIDE), the Ministry of Education, Culture and Science, the Ministry for Health, Welfare and Sports, the European Commission (DG XII), and the Municipality of Rotterdam. The authors are grateful to the study participants, the staff from the Rotterdam Study and the participating general practitioners and pharmacists.
Sorbs	This work was supported by grants from the Interdisciplinary Centre for Clinical Research at the University of Leipzig (B27 to AT, PK) from the German Diabetes Association (to AT, PK), a Travel Grant from BIF (to AT) and by the DHFD, Diabetes Hilfs- und Forschungsfonds Deutschland (PK). We thank all those who participated in the study. Sincere thanks are given to Anke Tönjes who was significantly involved in the design and performing of the Sorbs study. We also thank Knut Krohn (Microarray Core Facility of the Interdisciplinary Centre for Clinical Research, University of Leipzig) for the genotyping support. Reedik Mägi acknowledges financial support from the European Commission under a Marie Curie Intra-European Fellowship. The research of Inga Prokopenko is funded in part through the European Community's Seventh Framework Programme (FP7/2007-2013), ENGAGE project, grant agreement HEALTH-F4-2007- 201413.
WGHS	The WGHS is supported by HL 043851 and HL69757 from the National Heart, Lung, and Blood Institute and CA 047988 from the National Cancer Institute, the Donald W. Reynolds Foundation and the Fondation Leducq, with collaborative scientific support and funding for genotyping provided by Amgen.
YFS	The Young Finns Study has been financially supported by the Academy of Finland: grants 126925, 121584, 124282, 129378 (Salve), 117787 (Gendi), and 41071 (Skidi), the Social Insurance Institution of Finland, Kuopio, Tampere and Turku University Hospital Medical Funds (grant 9M048 for TeLeht), Juho Vainio Foundation, Paavo Nurmi Foundation, Finnish Foundation of Cardiovascular Research and Finnish Cultural Foundation, Tampere Tuberculosis Foundation and Emil Aaltonen Foundation (T.L). The expert technical assistance in the statistical analyses by Irina Lisinen is gratefully acknowledged.

Cohort	Acknowledgements (funding, personal, groups, …)
**Follow-Up Stage - Metabochip**	
ADVANCE	The ADVANCE study was supported by a grant from the Reynold's Foundation and NHLBI grant HL087647.
AMC-PAS	AMC-PAS is greatful to M.D. Trip MD, PhD and S. Sivapalaratnam MD for their input in collecting the data.
B58C noGWAS	Collection of DNA in the 1958 Birth Cohort was funded by the Medical Research Council grant G0000934 and Wellcome Trust grant 068545/Z/02. Dr Sue Ring and Dr Wendy McArdle (University of Bristol), and Mr Jon Johnson (Centre for Longitudinal Studies, Institute of Education, London) are thanked for help with data linkage. Work was undertaken at Great Ormond Street Hospital /University College London, Institute of Child Health which received a proportion of funding from the Department of Health's National Institute of Health Research ('Biomedical Research Centres' funding). The Medical Research Council provides funds for the MRC Centre of Epidemiology for Child Health.
BHS	The Busselton Health Study (BHS) acknowledges the generous support for the 1994/5 follow-up study from Healthway, Western Australia and the numerous Busselton community volunteers who assisted with data collection and the study participants from the Shire of Busselton. The Busselton Health Study is supported by The Great Wine Estates of the Margaret River region of Western Australia.
CARDIOGENICS	Sample collection in the Cardiogenics Consortium (http://www.cardiogenics.eu/web/) was funded by the 6th Framework Program of the European Union (LSHM-CT-2006-037593) and supported through the Cambridge Bioresource which is funded by the NIHR Cambridge Biomedical research Centre. We thank all the participants and clinicians involved in the recruitment process at Cambridge and Leicester (UK), Luebeck and Regensburg (Germany), and Paris (France).
CARDIOGENICS / THISEAS / AMC-PAS	This work was funded by the Wellcome Trust. We like to thank the members of the WTSI GenotypingFacility in particualr Sarah Edkins and Cordelia Langford
DIAGEN	The DIAGEN study was supported by the Commission of the European Communities, Directorate C - Public Health and Risk Assessment, Health & Consumer Protection, Grant Agreement number - 2004310 and by the Dresden University of Technology Funding Grant, Med Drive. We are grateful to all of the patients who cooperated in this study and to their referring physicians and diabetologists in Saxony.
DILGOM	The DILGOM study has been funded by the Academy of Finland (grant numbers 139635, 129494, 118065, 129322, 250207), the Orion-Farmos Research Foundation, the Finnish Foundation for Cardiovascular Research, and the Sigrid Jusélius Foundation.
DPS	The DPS has been financially supported by grants from the Academy of Finland (117844 and 40758, 211497, and 118590; The EVO funding of the Kuopio University Hospital from Ministry of Health and Social Affairs (5254), Finnish Funding Agency for Technology and Innovation (40058/07), Nordic Centre of Excellence on Systems biology in controlled dietary interventions and cohort studies, SYSDIET (070014), The Finnish Diabetes Research Foundation, Yrjö Jahnsson Foundation (56358), Sigrid Juselius Foundation, Juho Vainio Foundation and TEKES grants 70103/06 and 40058/07.

Cohort	Acknowledgements (funding, personal, groups, …)
DR's EXTRA	The DR.s EXTRA Study was supported by grants to R. Rauramaa by the Ministry of Education and Culture of Finland (627;2004-2011), Academy of Finland (102318; 123885), Kuopio University Hospital , Finnish Diabetes Association, Finnish Heart Association, Päivikki and Sakari Sohlberg Foundation and by grants from European Commission FP6 Integrated Project (EXGENESIS); LSHM-CT-2004-005272, City of Kuopio and Social Insurance Institution of Finland (4/26/2010).
EAS	The EAS was funded by the British Heart Foundation. Genotyping was funded by the Chief Scientist Office, Scotland, and undertaken at the Wellcome Trust Clinical Research Facility in Edinburgh.
EGCUT	EGCUT received financing by FP7 grants (201413, 245536), also received targeted financing from Estonian Government SF0180142s08 and direct funding from the ministries of research and science and social affairs. EGCUT studies are funded by University of Tartu in the frame of Center of Translational Genomics and by the European Union through the European Regional Development Fund, in the frame of Centre of Excellence in Genomics. We acknowledge EGCUT personelle, especially Ms. M. Hass and Mr V. Soo. EGCUT data analyzes were carried out in part in the High Performance Computing Center of University of Tartu.
Ely	The MRC Ely Study was funded by the Medical Research Council and the Wellcome Trust. We are most grateful to all study participants and to the staff of the St. Mary's Street Surgery, Ely. We thank all the staff who worked on the study.
EPIC	The EPIC Norfolk Study is funded by Cancer Research United Kingdom and the Medical Research Council.
FENLAND	The Fenland Study is funded by the Wellcome Trust and the Medical Research Council, as well as by the Support for Science Funding programme and CamStrad. We are grateful to all the volunteers for their time and help, and to the General Practitioners and practice staff for help with recruitment. We thank the Fenland Study co-ordination team and the Field Epidemiology team of the MRC Epidemiology Unit for recruitment and clinical testing.
FIN-D2D 2007	The FIN-D2D study has been financially supported by the hospital districts of Pirkanmaa, South Ostrobothnia, and Central Finland, the Finnish National Public Health Institute (current National Institute for Health and Welfare), the Finnish Diabetes Association, the Ministry of Social Affairs and Health in Finland, the Academy of Finland (grant number 129293),Commission of the European Communities, Directorate C-Public Health (grant agreement no. 2004310) and Finland's Slottery Machine Association.
FUSION	Support for FUSION was provided by NIH grants R01-DK062370 (to M.B.), R01-DK072193 (to K.L.M.), and intramural project number 1Z01-HG000024 (to F.S.C.). Genome-wide genotyping was conducted by the Johns Hopkins University Genetic Resources Core Facility SNP Center at the Center for Inherited Disease Research (CIDR), with support from CIDR NIH contract no. N01-HG-65403.

Cohort	Acknowledgements (funding, personal, groups, …)
GLACIER	The GLACIER Study was funded by grants from the Swedish Diabetes Association, Swedish Heart-Lung Foundation, Swedish Research Council, Medical Research Foundation of Umeå University, and Novo Nordisk (all to PWF).<br>We thank the participants for there outstanding contributions to the GLACIER Study. We also thank the staff of the Umeå Medical Biobank, especially Göran Hallmans, Åsa Agren, John Hutilainen, and Ann-Marie Ahren for data reteival and organisation and Kerstin Enqusit and Tore Johansson for expert assistance with DNA extraction and plating. The GLACIER Study is nested within the Västerbottens Intervention Project (VIP); we thank the staff of the VIP Study for phenotype data collection, particularly Lars Wennehall who leads the VIP Study.<br>Inês Barroso acknowledges funding from the Wellcome Trust grant 098051, United Kingdom NIHR Cambridge Biomedical Research Centre and the MRC Centre for Obesity and Related Metabolic Diseases.<br>We would like to thank Emma Gray, Douglas Simpkin, Sarah Hunt and staff of the WTSI Sample Logistics, Genotyping and Variation Informatics Facilities.
GO-DARTS	We acknowledge the support of the Health Informatics Centre, University of Dundee for managing and supplying the anonymised data and NHS Tayside, the original data owner. We are grateful to all the participants who took part in the Go-DARTS study, to the general practitioners, to the Scottish School of Primary Care for their help in recruiting the participants, and to the whole team, which includes interviewers, computer and laboratory technicians, clerical workers, research scientists, volunteers, managers, receptionists, and nurses.<br>Financial Disclosure:<br>The Wellcome Trust provides support for Wellcome Trust United Kingdom Type 2 Diabetes Case Control Collection (Go-DARTS) and the Scottish Health Informatics Programme. Further informatics support is provided by the Chief Scientist Office of Scotland. This work was also supported by the UK Medical Research Council (G0601261)
HNR	We thank the Heinz Nixdorf Foundation, Germany, for the generous support of this study. We acknowledge the support of the Sarstedt AG & Co. (Nümbrecht, Germany) concerning laboratory equipment. We are grateful to Prof. Karl-Heinz Joeckel (Institute for Medical Informatics, Biometry and Epidemiology (IMIBE), University Hospital of Essen, University of Duisburg-Essen, Essen, Germany) and Prof. Dirk Schadendorf (Clinic Department of Dermatology, University Hospital Essen, University Duisburg-Essen, Essen, Germany) for funding this study. We are also thankful to Dr. Thomas W Mühleisen (Institute of Human Genetics, University of Bonn, Bonn, Germany) for carrying out the genotyping.
HUNT2	The Nord-Trøndelag Health Study (The HUNT Study) is a collaboration between HUNT Research Centre (Faculty of Medicine, Norwegian University of Science and Technology NTNU), Nord-Trøndelag County Council, Central Norway Health Authority, and the Norwegian Institute of Public Health.
IMPROVE	The IMPROVE study was supported by the European Commission (Contract number: QLG1-CT-2002-00896), the Swedish Heart-Lung Foundation, the Swedish Research Council (projects 8691 and 0593), the Knut and Alice Wallenberg Foundation, the Torsten and Ragnar Söderberg Foundation, the Foundation for Strategic Research, the Stockholm County Council (project 562183), the Strategic Cardiovascular and Diabetes Programmes of Karolinska Institutet and Stockholm County Council, Academy of Finland (Grant #110413) the British Heart Foundation (RG2008/08 and RG2008/014) and the Italian Ministry of Health (Ricerca Corrente).

Cohort	Acknowledgements (funding, personal, groups, …)
KORA	The **MONICA/KORA Augsburg studies** were financed by the Helmholtz Zentrum München, German Research Center for Environmental Health, Neuherberg, Germany, and supported by grants from the German Federal Ministry of Education and Research (BMBF). Part of this work was financed by the German National Genome Research Network (NGFNPlus, project number 01GS0834, 01GS0823) and through additional funds from the University of Ulm. Furthermore, the research was supported within the Munich Center of Health Sciences (MC Health) as part of LMU innovative. The KORA analysts in Regensburg were supported by the Bundesministerium für Bildung und Forschung/BMBF (01ER1206).
LURIC	LURIC received funding through the 6th Framework Program (integrated project Bloodomics, grant LSHM-CT-2004-503485) and 7th of Framework Program (integrated project AtheroRemo, Grant Agreement number 201668) of the European Union. The authors extend appreciation to the participants of the LURIC study without their collaboration this article would not have been written. We thank the LURIC study team either temporarily or permanently involved in patient recruitment, sample and data handling, and the laboratory staff at the Ludwigshafen General Hospital and the Universities of Freiburg and Ulm, Germany.
METSIM	The METSIM study was funded by the Academy of Finland (grants no. 77299 and 124243).

Cohort	Acknowledgements (funding, personal, groups, …)
MORGAM	The MORGAM study was part funded through the European Community's Sixth Framework Programme Cardiogenics project, grant agreement LSHM-CT-2006-037593 and Seventh Framework Programme ENGAGE project, grant agreement HEALTH-F4-2007-201413.<br><br>Sites and key personnel of contributing MORGAM Centres:<br><br>Finland<br>FINRISK, National Institute for Health and Welfare, Helsinki: V. Salomaa (principal investigator), A. Juolevi, E. Vartiainen, P. Jousilahti; ATBC, National Institute for Health and Welfare, Helsinki: J. Virtamo (principal investigator), H. Kilpeläinen; MORGAM Data Centre, National Institute for Health and Welfare, Helsinki: K. Kuulasmaa (responsible person), Z. Cepaitis, A. Haukijärvi, B. Joseph, J. Karvanen, S. Kulathinal, M. Niemelä, O. Saarela; MORGAM Central Laboratory, National Institute for Health and Welfare, Helsinki: M. Perola (responsible person), P. Laiho, M. Sauramo.<br><br>France<br>National Coordinating Centre, National Institute of Health and Medical Research (U258), Paris: P. Ducimetière (national coordinator), A. Bingham; PRIME/Strasbourg, Department of Epidemiology and Public Health, EA 3430, University of Strasbourg, Faculty of Medicine, Strasbourg: D. Arveiler (principal investigator), B. Haas, A. Wagner; PRIME/Toulouse, Department of Epidemiology, Toulouse University School of Medicine, Toulouse: J. Ferrières (Principal Investigator), J-B. Ruidavets, V. Bongard, D. Deckers, C. Saulet, S. Barrere; PRIME/Lille, Department of Epidemiology and Public Health, INSERM U744-Université Lille Nord de France – Institut Pasteur de Lille: P. Amouyel (principal investigator), M. Montaye, B. Lemaire, S. Beauchant, D. Cottel, C. Graux, N. Marecaux, C. Steclebout, S. Szeremeta; MORGAM Laboratory, INSERM U937, Paris: F. Cambien (responsible person), L. Tiret, V. Nicaud.<br><br>Italy<br>Centro Ricerche EPIMED - Epidemiologia e Medicina Preventiva, Dipartimento di Medicina Clinica e Sperimentale. Università degli Studi dell'Insubria, Varese: M. Ferrario (principal investigator), G. Veronesi and Dipartimento di Medicina Sperimentale. Università degli Studi Milano-Bicocca, Monza: G. Cesana, P Brambilla.<br><br>United Kingdom<br>PRIME/Belfast, Queen's University Belfast, Belfast, Northern Ireland: F. Kee (principal investigator) A. Evans (former principal investigator), J. Yarnell, E. Gardner; MORGAM Coordinating Centre, Queen's University Belfast, Belfast, Northern Ireland: A. Evans (MORGAM coordinator), S. Cashman, F Kee.<br><br>MORGAM Management Group: A. Evans (chair, Belfast, UK), S. Blankenberg (Hamburg, Germany), F. Cambien (Paris, France), M. Ferrario (Varese, Italy), K. Kuulasmaa (Helsinki, Finland), A. Palotie (Cambridge, UK), M. Perola (Helsinki, Finland), A. Peters (Neuherberg, Germany), V. Salomaa (Helsinki, Finland), H. Tunstall-Pedoe (Dundee, Scotland), P.G. Wiklund (Umeå, Sweden); Previous members: K. Asplund (Stockholm, Sweden), L. Peltonen (Helsinki, Finland), D. Shields (Dublin, Ireland), B. Stegmayr (Umeå, Sweden).

Cohort	Acknowledgements (funding, personal, groups, …)
NSHD	This work was funded by the Medical Research Council. We are very grateful to the members of this birth cohort for their continuing interest and participation in the study.
PIVUS	Genotyping was performed by the SNP&SEQ Technology Platform in Uppsala (www.genotyping.se). We thank Tomas Axelsson, Ann-Christine Wiman and Caisa Pöntinen for their excellent assistance with genotyping. The SNP Technology Platform is supported by Uppsala University, Uppsala University Hospital and the Swedish Research Council for Infrastructures. E.I. is supported by grants from the Swedish Research Council, the Swedish Heart-Lung Foundation, the Swedish Foundation for Strategic Research, and the Royal Swedish Academy of Science.
SCARFSHEEP	The SCARFSHEEP study was supported by the Swedish Heart-Lung Foundation, the Swedish Research Council (projects 8691 and 0593), the Knut and Alice Wallenberg Foundation, the Torsten and Ragnar Söderberg Foundation, the Foundation for Strategic Research, the Stockholm County Council (project 562183) and the Strategic Cardiovascular and Diabetes Programmes of Karolinska Institutet and Stockholm County Council.
STR	This work was supported by grants from the National Institutes of Health (AG028555, AG08724, AG04563, AG10175, AG08861), the Swedish Research Council, the Swedish Heart-Lung Foundation, the Swedish Foundation for Strategic Research, the Royal Swedish Academy of Science, and ENGAGE (within the European Union Seventh Framework Programme, HEALTH-F4-2007-201413). Genotyping was performed by the SNP&SEQ Technology Platform in Uppsala (www.genotyping.se). We thank Tomas Axelsson, Ann-Christine Wiman and Caisa Pöntinen for their excellent assistance with genotyping. The SNP Technology Platform is supported by Uppsala University, Uppsala University Hospital and the Swedish Research Council for Infrastructures.
T2D-WTCCC	Research funding for sample collection, genotyping and data analysis for the T2D-WTCCC and other cohorts for which the Oxford group had responsibility came from the British Diabetes Association, BDA Research, Diabetes UK, Oxford NIHR Biomedical Research Centre, European Commission (ENGAGE: HEALTH-F4-2007-201413; EURODIA: LSHG-CT-2004-518153, Wellcome Trust (072960, 076113/B/04/Z, 076113/K/04/Z, 083270, 085301, 079557, 081682, 075491) UK Medical Research Council (G0000649, G0601261) and NIDDK (R01-DK-073490). In addition, Cecilia Lindgren is funded by WT086596/Z/08/Z (Wellcome Trust Research Career Development Fellowship); Reedik Mägi is funded by European Commission under the Marie Curie Intra-European Fellowship; and Mark McCarthy receivespersonal funding from the Oxford NIHR Biomedical Research Centre.
THISEAS	Recruitment for THISEAS was partially funded by a research grant (PENED 2003) from the Greek General Secretary of Research and Technology; we thank all the dieticians and clinicians for their contribution to the project.

Cohort	Acknowledgements (funding, personal, groups, …)
Tromsø	The Tromsø study has been supported by the University of Tromsø and the Norwegian Research Council (project number 185764).
ULSAM	Genotyping was performed by the SNP&SEQ Technology Platform in Uppsala (www.genotyping.se). We thank Tomas Axelsson, Ann-Christine Wiman and Caisa Pöntinen for their excellent assistance with genotyping. The SNP Technology Platform is supported by Uppsala University, Uppsala University Hospital and the Swedish Research Council for Infrastructures. E.I. is supported by grants from the Swedish Research Council, the Swedish Heart-Lung Foundation, the Swedish Foundation for Strategic Research, and the Royal Swedish Academy of Science.
Whitehall II	The WHII study has been supported by grants from the Medical Research Council; Economic and Social Research Council; BHF; Health and Safety Executive; Department of Health; National Heart Lung and Blood Institute (HL36310), US, NIH: National Institute on Aging (AG13196), US, NIH; Agency for Health Care Policy Research (HS06516); and the John D and Catherine T MacArthur Foundation Research Networks on Successful Midlife Development and Socioeconomic Status and Health. Genotyping in WHII was supported by BHF grant PG/07/133/24260. Whitehall II genotyping was in part supported by a MRC-GSK pilot programme grant (ID 85374).
